# Variation of the Emission Efficiency and Wavelength from Fluorescent Zinc Salen Complexes upon Systematic Structural Modifications

**DOI:** 10.1021/acsomega.2c04714

**Published:** 2022-08-17

**Authors:** Takuya Kurahashi

**Affiliations:** Department of Nutrition Science, Faculty of Nursing and Nutrition, University of Nagasaki, Siebold, Manabino, Nagayo, Nagasaki 851-2195, Japan

## Abstract

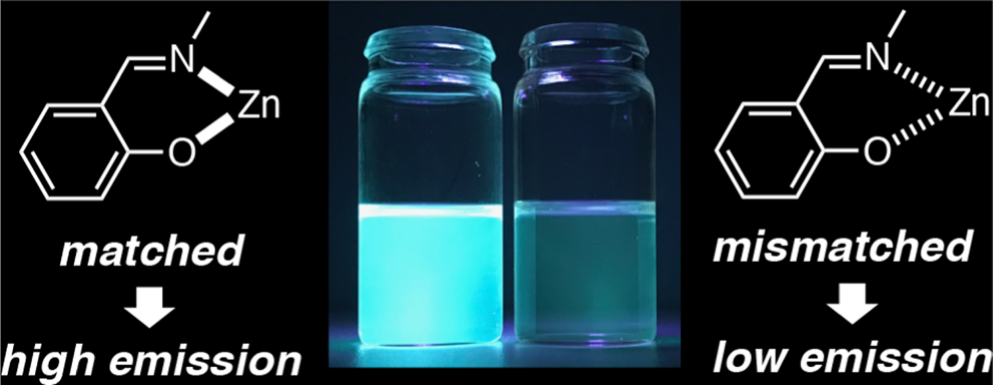

Understanding the photophysical properties of metal salen complexes is not straightforward because the emission efficiency is altered irregularly upon structural modifications. The present study prepared zinc salen complexes with systematic structural variations to pinpoint critical factors to determine the emission efficiency. One of the important experimental observations is the regiochemistry of a phenolate substituent affecting emission efficiency from a salicylidene fluorophore, which is nicely assigned as arising from the photoexcited electronic structure of metal salen complexes. Another significant finding is the thermal fluctuation of a salen ligand arising from the mismatched ligand–metal interaction, which has a significant impact on fluorescence lifetime. The present study sheds light on hidden factors that alter photophysical properties of a metal salen complex, which provide valuable insights into designing new photoactive salen ligands.

## Introduction

Metal salen complexes are well known as a useful framework for catalysts and materials.^1,2^ Metal salen complexes are readily prepared by mixing two independently prepared organic components (salicylaldehyde and amine) and metal ions, generating a wide variety of stereochemically and electronically distinct complexes. Such synthetic modularity is the key for their utility.

**Chart 1:**
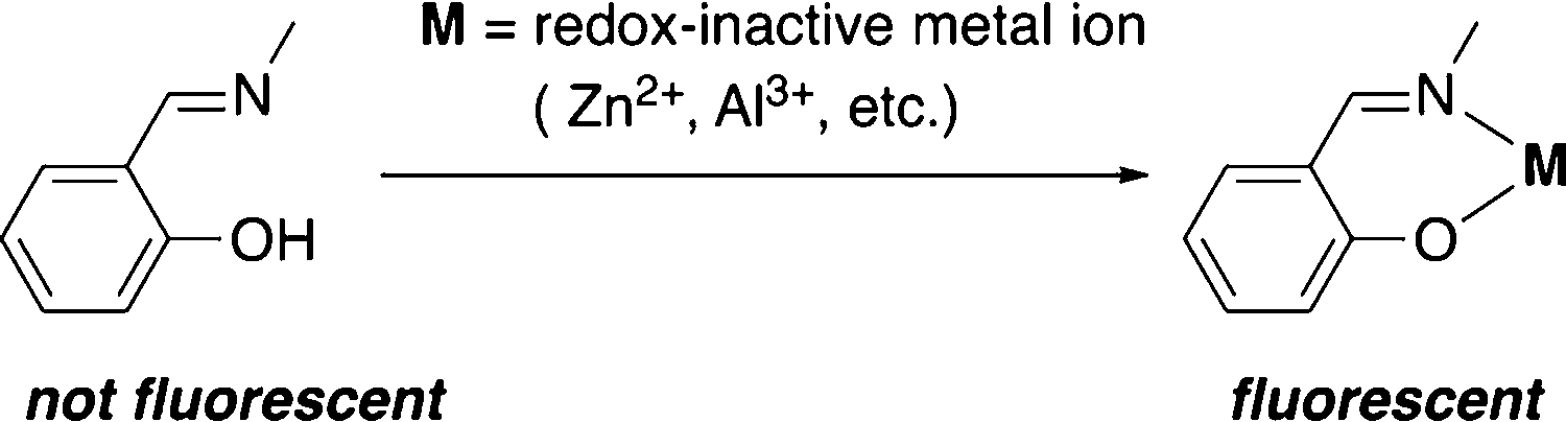
Salicylidene ring as a minimum fluorescence unit.

Some of metal salen complexes show moderate fluorescence without any apparent photoactive group, which is attractive from the viewpoint to expand the utility of metal salen complexes as photoactive catalysts and materials. According to the early studies,^3,4^ the minimum fluorescence unit is the salicylidene ring consisting of the phenolate and the azomethine groups, but the complexation with redox inactive metal ion such as zinc and aluminum is required to attain fluorescence^5,6^ (Chart 1).

The fluorescence from metal salen complexes has already found applications as organic light-emitting diodes^7^ and sensors for amines,^8^ nitro compounds,^9^ phosphates,^10,11^ fluorides,^12^ and metal ions.^13−21^ Mechanistic details have also been of interest, and fluorescence properties such as emission wavelength and emission efficiency have been investigated for a variety of metal salen complexes.^22−37^ Previous studies have shown that the emission wavelength could be modulated by incorporating substituents of different electron-donating ability such as dimethylamino and ester groups to the phenolate moiety of metal salen complexes, which may be reasonably explained by density functional theory-calculated highest occupied molecular orbital (HOMO) and lowest unoccupied molecular orbital (LUMO) levels.^26,27,37^ However, the drastic difference in fluorescence quantum yields ranging from 0.2 to 14.4%^26^ is apparently difficult to rationalize by HOMO and LUMO considerations.

The present study investigates fluorescence quantum yields and fluorescence lifetimes of zinc salen complexes upon systematic structural modifications to clarify critical structural factors. One of the interesting findings is the positive effect of substituents at the 3- and 5- positions of the phenolates, which is identified as the very characteristic feature for the photoexcitation of a salen complex, leading to a new mechanistic proposal. Another important finding is that the ligand moiety of a metal salen complex is thermally fluctuating to significantly different degree depending on the ligand structure. The present study shows that the ligand fluctuation is a dominant factor for the emission efficiency in the case of a metal salen complex. These two findings, which are not evident from the previous experimental and theoretical studies, are thus of great value in understanding photophysical properties of a metal salen complex.

## Results and Discussion

### Characterization of Zinc Complexes

**Chart 2:**
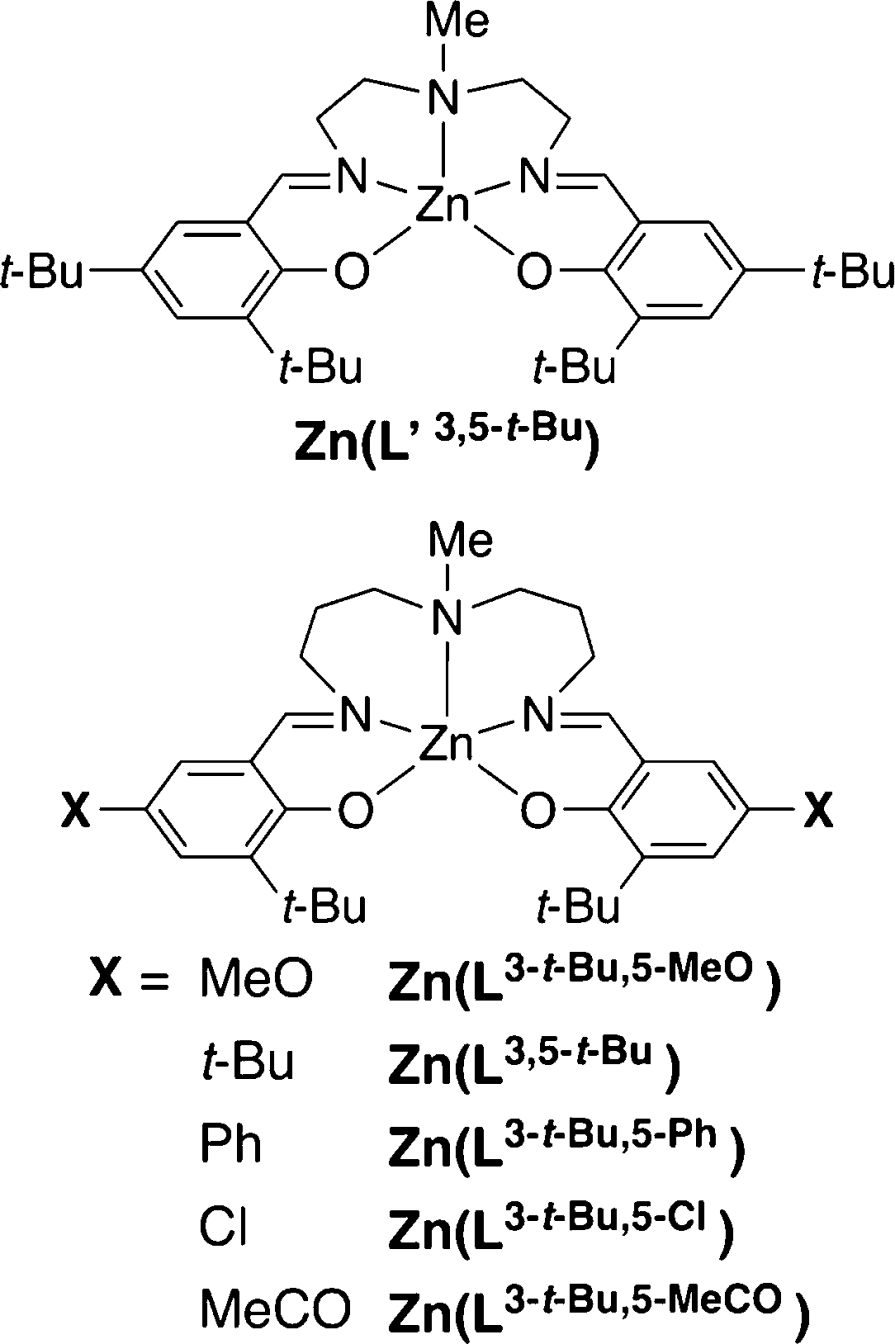
Zinc complexes with systematic structural variations.

**Chart 3:**
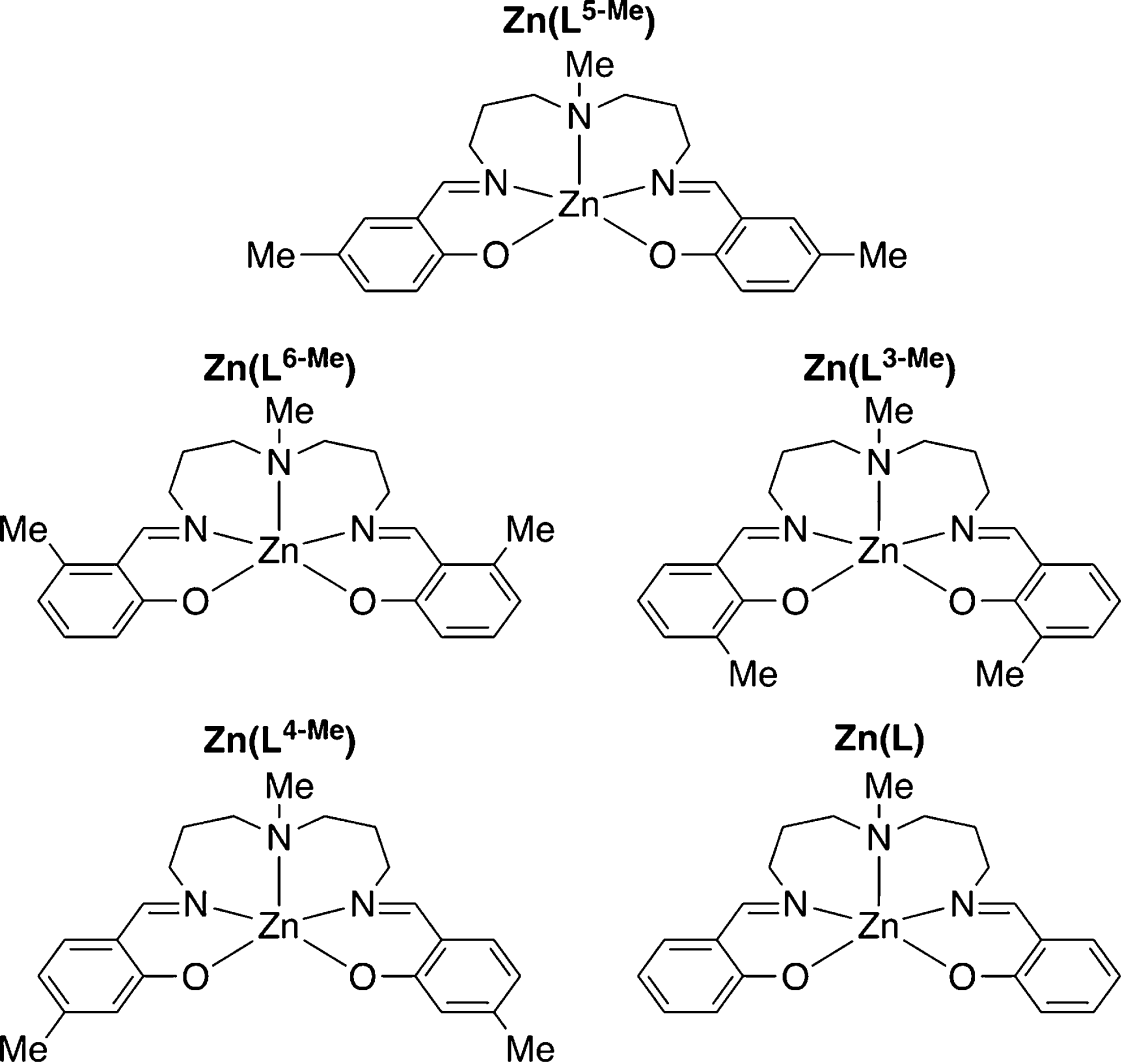
Zinc complexes to investigate the regiochemical effect on the emission efficiency.

**Chart 4:**
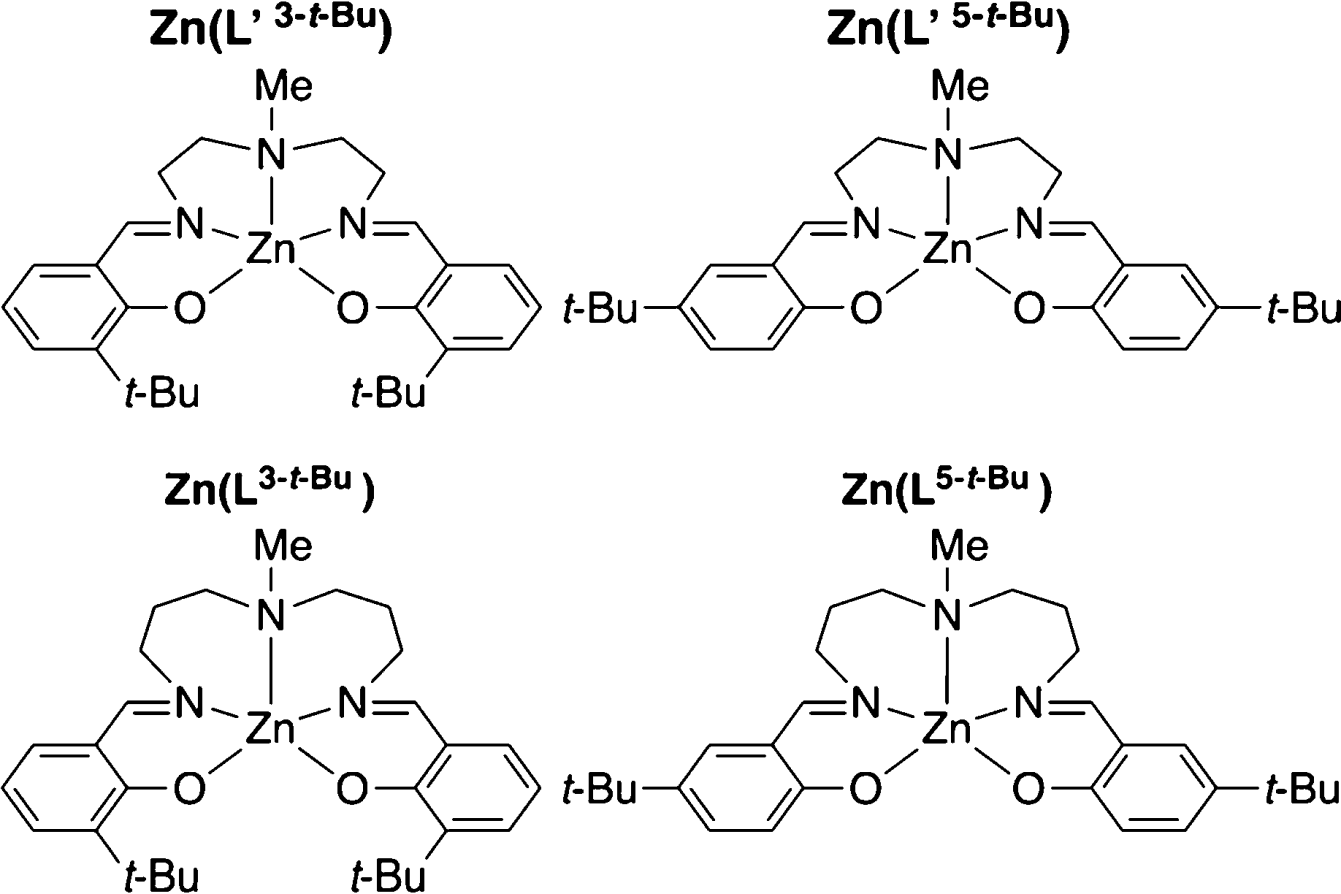
Zinc complexes to investigate the steric effect.

Familiar four-coordinate salen complexes with zinc are insoluble in common organic solvents, and then, the present study employs five-coordinate zinc complexes, as shown in Chart 2 and those in Charts 3 and 4 (vide infra), which were characterized with ^1^H, ^13^C NMR spectroscopy, mass spectrometry, and elemental analysis. According to the previous study,^38^ the Zn(L^3,5-*t*-Bu^) complex has a monomeric Zn center coordinated by three nitrogen ligands and two oxygen ligands. The other zinc complexes utilized in this study are also monomeric, as indicated by mass spectrometry (Figure S1–S13).

### Electrochemical Properties and the Emission Wavelength

**Figure 1:**
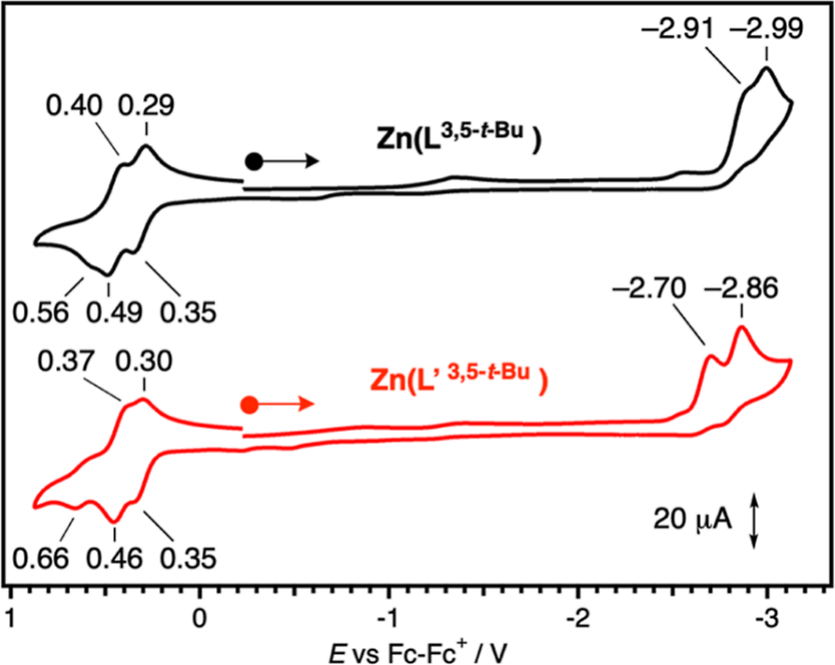
Cyclic voltammograms of Zn(L^3,5-*t*-Bu^) (black line) and Zn(L′ ^3,5-*t*-Bu^) (red line) in acetonitrile containing 0.1 M of Bu_4_NOTf at 298 K under an argon atmosphere. The starting point of scans are indicated by the circles, and the direction of scans are indicated by the arrows.

HOMO and LUMO energy levels of the present fluorophores are estimated by measuring redox potentials. Figure 1 shows cyclic voltammograms of Zn(L^3,5-*t*-Bu^) and Zn(L′ ^3,5-*t*-Bu^) complexes, which have a propylene and ethylene linker, respectively. Both Zn(L^3,5-*t*-Bu^) and Zn(L′ ^3,5-*t*-Bu^) show the first oxidation wave at the same oxidation potential of 0.35 V, which comes from the one-electron oxidation of the phenolate moiety of a common structure. On the other hand, the reduction waves of Zn(L^3,5-*t*-Bu^) and Zn(L′ ^3,5-*t*-Bu^) appear at different potentials of −2.91 and −2.70 V, respectively. The difference in the reduction waves possibly arise from the one-electron reduction of the imino group that is adjacent to the propylene or ethylene linker as the only structural difference in Zn(L^3,5-*t*-Bu^) and Zn(L′ ^3,5-*t*-Bu^).

**Figure 2:**
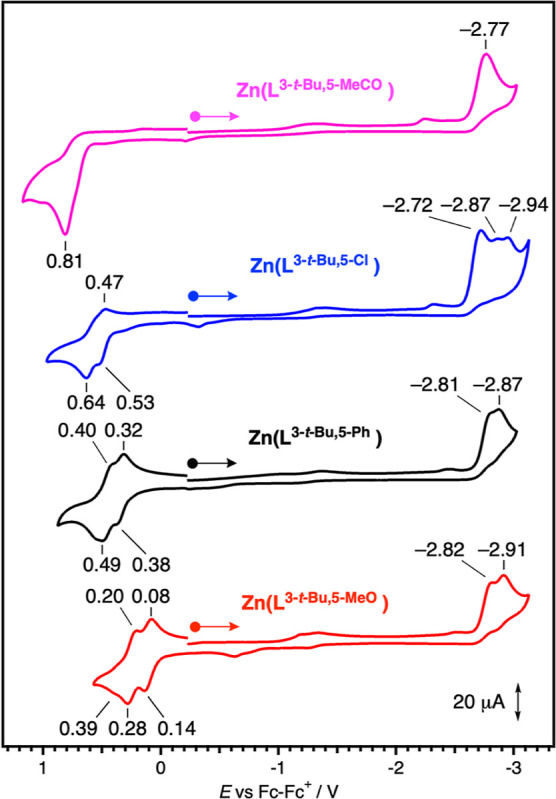
Cyclic voltammograms of zinc complexes in acetonitrile containing 0.1 M of Bu_4_NOTf at 298 K under an argon atmosphere. The starting point of scans are indicated by the circles, and the direction of scans are indicated by the arrows.

Effects of substituted phenolates were investigated. As shown in Figure 2, an oxidation potential is significantly increased in the order of MeO (0.14 V) < *t*-Bu (0.35 V) < Ph (0.38 V) < Cl (0.53 V) < MeCO (0.81 V) as a substituent at the 5-positions, indicating that the HOMO energy level is increased in this order. In contrast, reduction waves, which correspond to the LUMO energy level, are observed in a narrower range (−2.72 to −2.91 V) irrespective of a substituent on the phenolates. The relatively large difference in oxidation potentials upon exchanging substituents on the phenolate is reasonable because the one-electron oxidation of the salen-type metal complex occurs at the phenolate moiety.^39^

**Figure 3:**
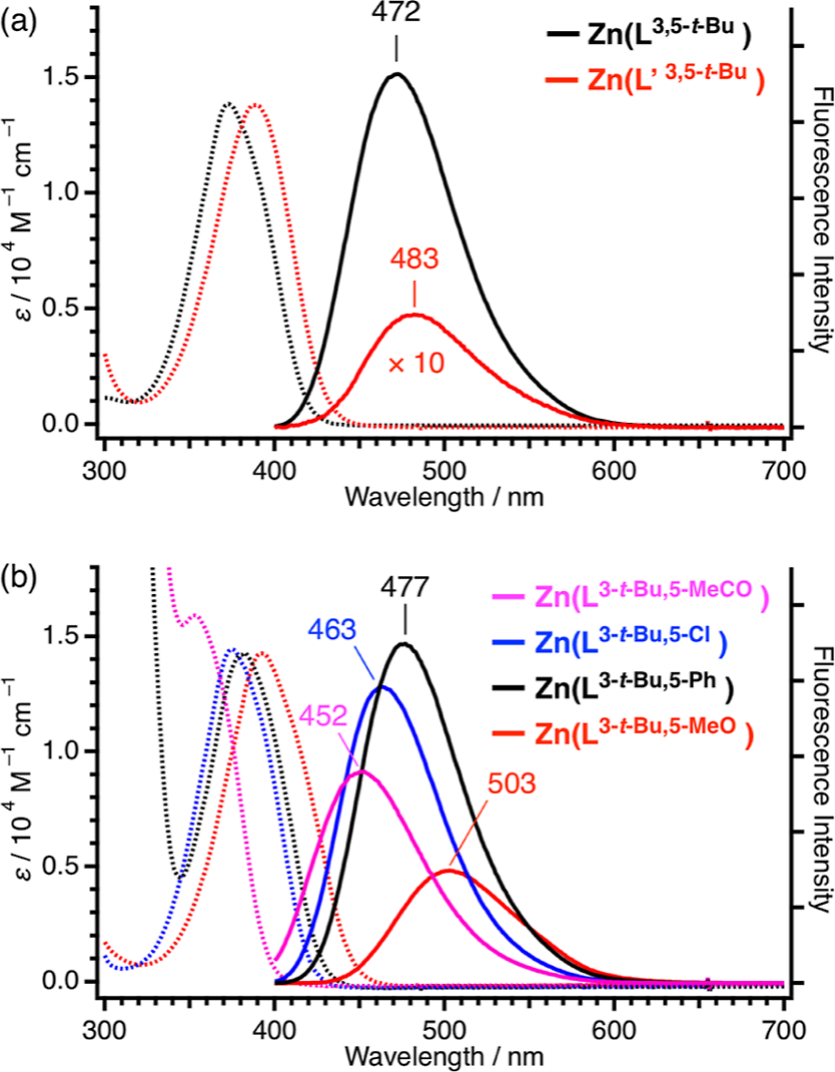
Absorption (dotted line) and fluorescence spectra (solid line) of (a) Zn(L^3,5-*t*-Bu^), Zn(L′ ^3,5-*t*-Bu^) and (b) Zn(L^3-*t*-Bu,5-MeCO^), Zn(L^3-*t*-Bu,5-Cl^), Zn(L^3-*t*-Bu,5-Ph^), and Zn(L^3-*t*-Bu,5-MeO^) in pyridine at 298 K. The fluorescence spectrum of Zn(L′ ^3,5-*t*-Bu^) is magnified 10-folds.

Figure 3a shows absorption and emission spectra of the Zn(L^3,5-*t*-Bu^) and Zn(L′ ^3,5-*t*-Bu^) complexes. Among several solvents tested (see Figure 7), pyridine was utilized to systematically compare the photophysical properties of the present zinc complexes. An emission wavelength maximum is shifted from 472 nm for Zn(L^3,5-*t*-Bu^) to 483 nm for Zn(L′ ^3,5-*t*-Bu^). The observed shift to a longer wavelength is consistent with the HOMO–LUMO band gaps, as estimated from CV measurements in which the difference between the oxidation and the reduction peak potentials of Zn(L^3,5-*t*-Bu^) (3.26 V) is larger than that of Zn(L′ ^3,5-*t*-Bu^) (3.05 V). Replacing the linkers from propylene in Zn(L^3,5-*t*-Bu^) to ethylene in Zn(L′ ^3,5-*t*-Bu^) shifts the reduction peak potential from −2.91 to −2.70 V without changing the oxidation peak potential, which is identified as a key factor for the difference in an emission wavelength maximum between Zn(L^3,5-*t*-Bu^) and Zn(L′ ^3,5-*t*-Bu^).

**Figure 4:**
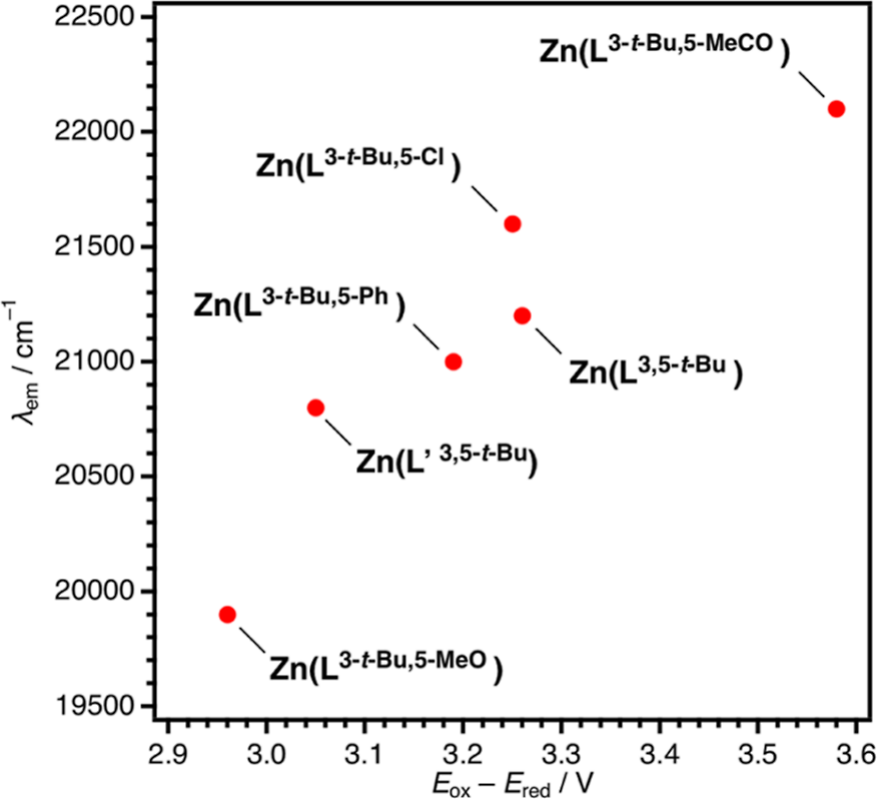
Plots of λ_em_ (cm^–1^) vs *E*_ox_ – *E*_red_ (V).

Figure 3b shows absorption and emission spectra of zinc complexes having a different substituent on phenolates. The shifts of an emission wavelength maximum are also in good agreement with the difference between the oxidation and the reduction peak potentials or the HOMO–LUMO band gaps. As shown in Figure 4, the emission wavelength maxima in cm^–1^ are almost proportional to the *E*_ox_ (oxidation peak potential) – *E*_red_ (reduction peak potential) values in V.

### Factors to Determine the Fluorescence Efficiency and Fluorescence Lifetime

Quantum yields of the present zinc complexes are significantly different, 1.1% for Zn(L′ ^3,5-*t*-Bu^) to 21% for Zn(L^3,5-*t*-Bu^), as summarized in Table 1. In contrast to the emission wavelength, the trend of quantum yields is not explained by the HOMO–LUMO energy levels or the redox potentials. Then, the correlation between a structural feature and emission efficiency was investigated in more detail.

**Table 1:** Photophysical and Electrochemical Data of Zinc Complexes^a^

	λ_max_ (nm)^b^	ε (M^–1^ cm^–1^)^b^	λ_em_ (nm)^c^	ϕ (%)^d^	τ (ns)^e^	*k*_r_ (ns^–1^)^f^	*k*_nr_ (ns^–1^)^f^	*E*_ox_ (V)^g^	*E*_red_ (V)^g^
Zn(L′^3,5-*t*-Bu^)	389	1.38 × 10^4^	480	1.1	1.06	0.010	0.93	0.35	–2.70
Zn(L^3,5-*t*-Bu^)	373	1.39 × 10^4^	472	21	5.06	0.042	0.16	0.35	–2.91
Zn(L^3-*t*-Bu,5-MeO^)	392	1.41 × 10^4^	503	7.6	2.55	0.030	0.36	0.14	–2.82
Zn(L^3-*t*-Bu,5-Ph^)	382	1.35 × 10^4^	477	20	3.98	0.050	0.20	0.38	–2.81
Zn(L^3-*t*-Bu,5-Cl^)	375	1.42 × 10^4^	463	17	5.06	0.034	0.16	0.53	–2.72
Zn(L^3-*t*-Bu,5-MeCO^)	354	1.59 × 10^4^	450	13	1.47	0.088	0.59	0.81	–2.77
Zn(L^3-*t*-Bu^)	366	1.31 × 10^4^	456	16	3.51	0.046	0.24		
Zn(L)	363	1.41 × 10^4^	443	4.6	1.78	0.026	0.54		
Zn(L^3-Me^)	367	1.41 × 10^4^	457	13	3.22	0.040	0.27		
Zn(L^4-Me^)	362	1.36 × 10^4^	443	5.3	1.46	0.036	0.65		
Zn(L^5-Me^)	375	1.31 × 10^4^	464	13	4.33	0.030	0.20		
Zn(L^6-Me^)	372	1.25 × 10^4^	455	3.3	1.03	0.032	0.94		

a Structures of zinc complexes are shown in Charts 2, 3, and 4.

b Absorption spectra were measured in pyridine.

c Fluorescence spectra were measured in deoxygenated pyridine with excitation at λ_ex_ = 390 nm.

d Fluorescence quantum yields using 9,10-diphenylanthracene as a standard (ϕ = 100%).

e Fluorescence lifetimes measured in deoxygenated pyridine with λ_ex_ = 375 nm.

f The radiative (*k*_r_) and non-radiative (*k*_nr_) decay constants are calculated as follows; *k*_r_ = ϕ/τ, *k*_nr_ = (1 – ϕ)/τ.

g Anodic oxidation peak potentials (*E*_ox_) and cathodic reduction peak potentials (*E*_red_) measured in deoxygenated acetonitrile, which are reported against the Fc/Fc^+^ couple.

**Figure 5:**
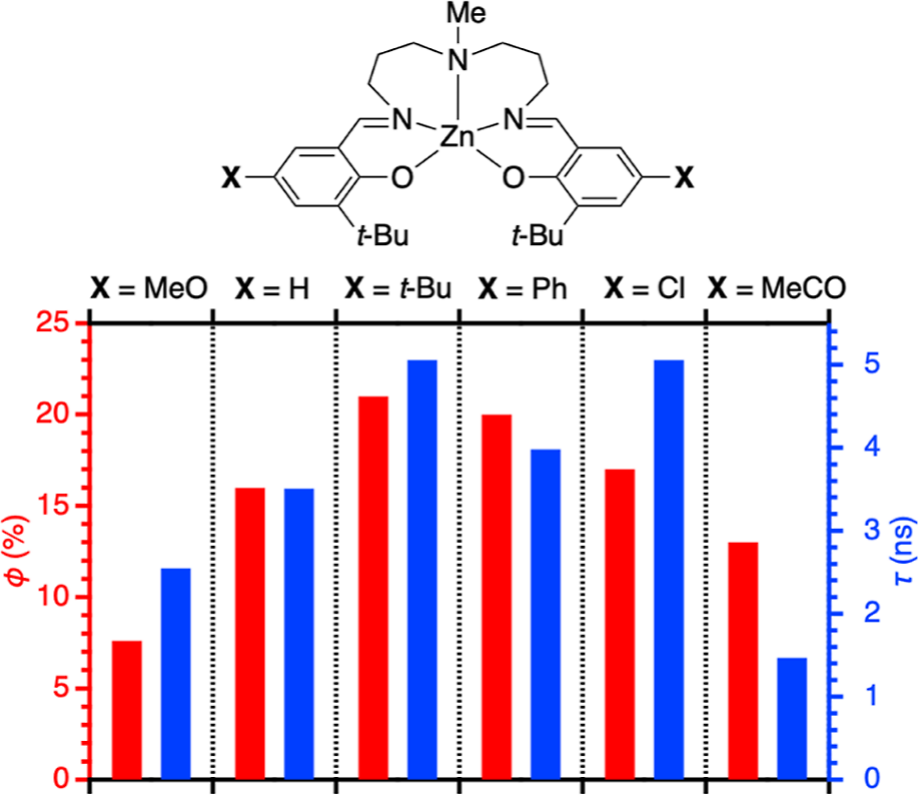
Electronic properties of substituents and fluorescence outcomes, as summarized in Table 1. The red bars show fluorescence quantum yields ϕ (%) relative to the left axis shown in red. The blue bars show fluorescence lifetimes τ (ns) relative to the right axis shown in blue.

One of the interesting observations is electronic properties of a phenolate substituents, which alter emission efficiency in a moderate manner (red bars, as shown in Figure 5). As compared to the hydrogen atom in Zn(L^3-*t*-Bu^), the incorporation of the *tert*-butyl group in Zn(L^3,5-*t*-Bu^) enhances the emission efficiency. The same is true for the phenyl group in Zn(L^3-*t*-Bu,5-Ph^), suggesting that aliphatic and aromatic substituents have positive effect on the emission efficiency. The Zn(L^3,5-*t*-Bu^) and Zn(L^3-*t*-Bu,5-Ph^) complexes give the best result regarding an emission efficiency. Upon the incorporation of a Cl substituent in Zn(L^3-*t*-Bu,5-Cl^), the emission efficiency is only slightly increased as compared to the Zn(L^3-*t*-Bu^) complex. The incorporation of the MeO and MeCO substituents into Zn(L^3-*t*-Bu,5-MeO^) and Zn(L^3-*t*-Bu,5-MeCO^) rather decreases the emission efficiency.

Figure 5 also shows fluorescence lifetimes (blue bars), which almost parallels fluorescence quantum yields (red bars). The *t*-Bu and Ph substituents yield a longer fluorescence lifetime as compared to the Zn(L^3-*t*-Bu^) complex, and the MeO and MeCO substituents give a negative effect on the fluorescence lifetime. This observation indicates that the emission efficiency is mainly determined by the fluorescence lifetime in the present system. As elucidated from the electrochemical parameters listed in Table 1, the MeO and MeCO substituents are electron-donating and electron-withdrawing groups (*E*_ox_ = 0.14 and 0.81 V, respectively), both of which induce the relaxation of the photoexcited state, resulting in a shorter fluorescence lifetime and a lower emission efficiency. In contrast, the *t*-Bu, Ph, and Cl substituents with the *E*_ox_ range between 0.35 and 0.53 V apparently contribute to a longer lifetime of the photoexcited state.

**Figure 6:**
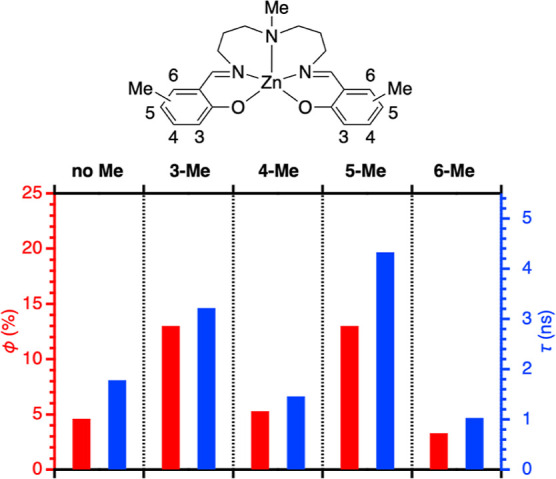
Positions of the methyl groups and fluorescence outcomes, as summarized in Table 1. The red bars show fluorescence quantum yields ϕ (%) relative to the left axis shown in red. The blue bars show fluorescence lifetimes τ (ns) relative to the right axis shown in blue.

To further investigate the role of the substituents on the photoexcited state, the regiochemistry of the substituents on the salicylidene rings was investigated using zinc complexes having a methyl group at a different position (Chart 3). The photophysical data are summarized in Table 1. As shown in Figure 6, the methyl groups at the 3- and 5-positions improve the fluorescence lifetime and the fluorescence quantum yield, as compared to the non-substituted complex. However, the methyl groups at the 4- and 6-positions have no positive effect. This observation indicates that the photoexcited state of the present system has a unique electronic structure possessing a relaxation pathway through the 3- and 5-postions of the salicylidene ring. Interestingly, the relaxation pathway could be interrupted by incorporating a proper substituent such as alkyl, aryl, and chloro groups. It was confirmed that repeated fluorescence measurements did not change fluorescence intensity of the Zn(L) complex, indicating that a non-substituted phenolate moiety in Zn(L) is not photochemically modified under the measurement conditions.

Table 1 also lists radiative (*k*_r_) and non-radiative (*k*_nr_) decay constants that are calculated from quantum yield (ϕ) and fluorescence lifetime (τ) values. The Zn(L^3-*t*-Bu,5-MeO^) complex shows a larger *k*_r_ value among others, suggesting that the MeCO substituent at the 5-position gives an unfavorable effect on emission. Regarding the non-radiative pathway, the Zn(L′^3,5-*t*-Bu^) and Zn(L^6-Me^) complexes show larger *k*_nr_ values. The methyl group at the 6-position in Zn(L^6-Me^) generates severe steric hindrance with the neighboring azomethine group to give a negative impact on the salicylidene chromophore, which might be a reason for the faster non-radiative decay. The low emission efficiency of Zn(L′^3,5-*t*-Bu^) is investigated in more detail in the next section.

**Figure 7:**
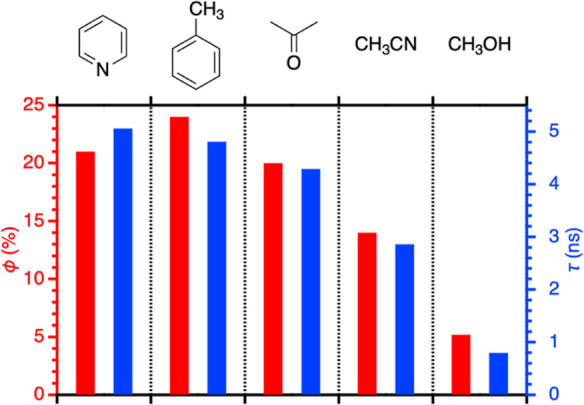
Solvent effects on the fluorescence properties, as summarized in Table S1 (Supporting Information). The red bars show fluorescence quantum yields ϕ (%) relative to the left axis shown in red. The blue bars show fluorescence lifetimes τ (ns) relative to the right axis shown in blue.

Solvent effects were investigated, and the results are shown in Figure 7 (the data are included in Table S1). The fluorescence properties in pyridine, toluene, and acetone are almost the same, indicating that the difference in solvent polarity between toluene and acetone has no impact in the present system. The present study employs Lewis basic pyridine as a measurement solvent to avoid any unexpected effect from the Lewis acidic Zn center,^40^ but the results shown in Figure 7 indicates that the Lewis basicity of pyridine is not an important factor. The use of the protic methanol solvent significantly decreases the emission efficiency.

### Another Aspect Related to the Emission Efficiency

The previous section systematically investigates a variation of the substituents on the light-absorbing salicylidene ring to clarify the structural factors that affect the emission efficiency. However, the structure of a light-absorbing moiety is not the only factor. The most remarkable example is largely different emission efficiency from Zn(L′ ^3,5-*t*-Bu^) and Zn(L^3,5-*t*-Bu^) (1.1 and 21%, respectively, as shown in Table 1), although the light-absorbing π-conjugated system is exactly the same (Chart 2). The difference in the emission efficiency could not be ascribed to the HOMO–LUMO band gap because the electrochemical properties of Zn(L′ ^3,5-*t*-Bu^) and Zn(L^3,5-*t*-Bu^), which show excellent correlation with emission wavelength, cannot explain largely different emission efficiency.

**Figure 8:**
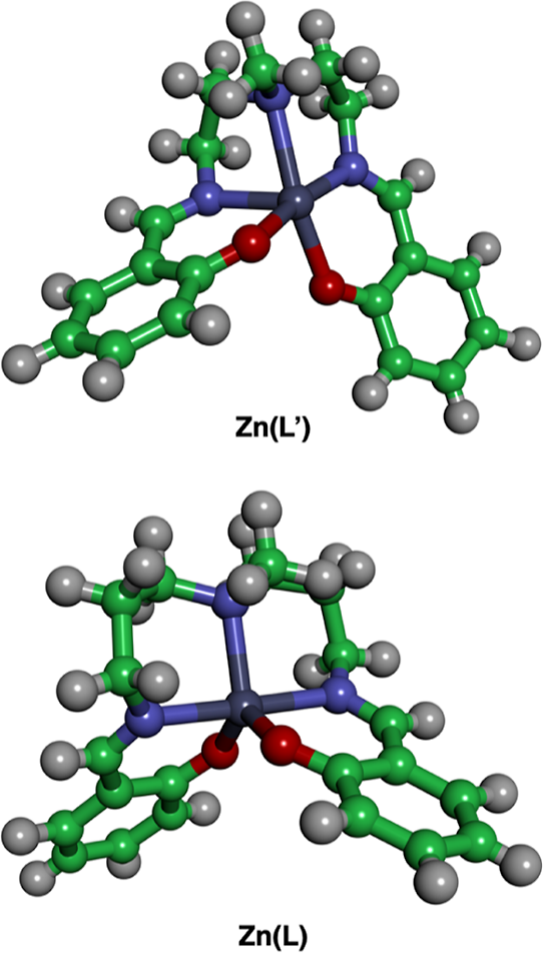
Coordination geometries of Zn(L′) and Zn(L), which have ethylene and propylene linkers, respectively.

**Figure 9:**
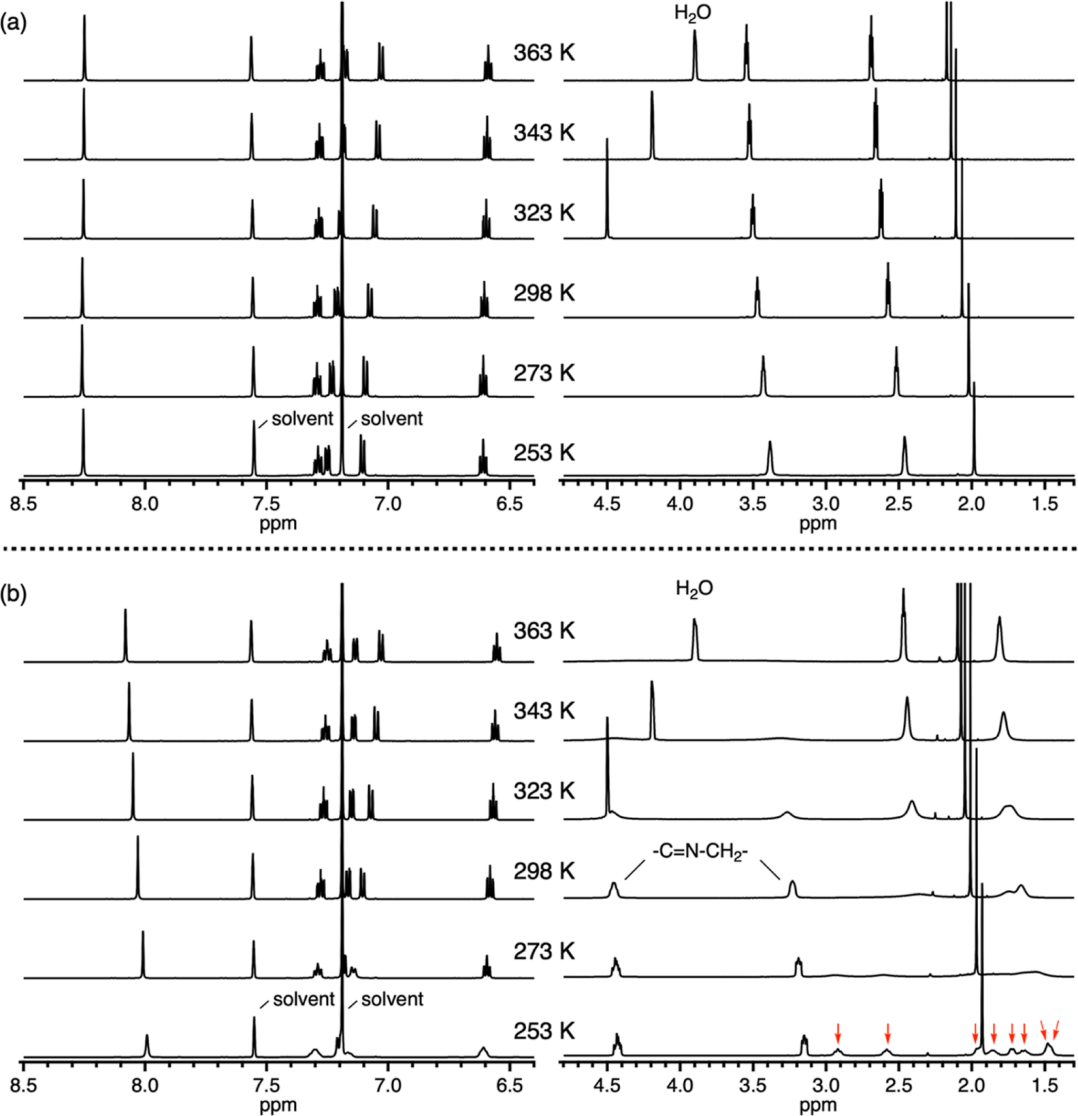
Variable-temperature ^1^H NMR spectra of (a) non-substituted Zn(L′) having ethylene linkers and (b) non-substituted Zn(L) having propylene linkers (10 mM, pyridine-*d*_5_).

The Zn(L′ ^3,5-*t*-Bu^) and Zn(L^3,5-*t*-Bu^) complexes only differ in a length of the methylene bridges in a triamine moiety. Then, the ethylene and propylene bridges in a triamine moiety were investigated with ^1^H NMR spectroscopy. Figure 8 shows structural models for Zn(L′) and Zn(L), which are reproduced from the previous X-ray structures.^38,40^ The coordination geometry of Zn(L′) is square-pyramidal, while the Zn(L) complex adopts a less strained trigonal bipyramidal structure. The metal-free L′ and L ligands are symmetric but lose the symmetry upon complexation with the zinc ion. The left and right halves of the ligand in both Zn(L′) and Zn(L) are positioned in a different environment. Figure 9a shows variable-temperature ^1^H NMR spectra of the non-substituted Zn(L′) complex having ethylene linkers. The Zn(L′) complex gives two triplet signals at 3.47 and 2.57 ppm (298 K), which are readily assigned as arising from the ethylene linkers. These signals are slightly shifted downfield upon raising the temperature from 253 to 363 K. The left and right halves of the linker moiety are not resolved, and the ^1^H NMR signals are averaged in the NMR timescale.

The ^1^H NMR spectra of the Zn(L) complex having propylene linkers are strikingly different (Figure 9b). At 298 K, the only observable feature in the aliphatic region is two signals at 4.46 and 3.23 ppm, which are significantly broadened upon increasing the temperature. These two signals are assigned as arising from −CH_2_– adjacent to the −C̅N– moiety using correlation spectroscopy (COSY) and nuclear Overhauser effect spectroscopy (NOESY) experiments (Figure S14–S16). Remarkably, the −CH_2_– signals adjacent to the −C̅N– moiety are separately observed in Zn(L) below 323 K, which is in clear contrast to the averaged single signal in Zn(L′) over the entire temperature range.

The temperature dependence of the ^1^H NMR signals in the region from 1.4 to 3.0 ppm is more informative. As shown in Figure 9b, the ^1^H NMR spectrum at 253 K shows eight resolved signals, as indicated with red arrows, which are assigned as arising from four methylenes. These signals are broadened out to disappear at 273 K and are finally merged into only two averaged signals at 2.47 and 1.81 ppm above 323 K. According to these observations, the Zn(L) complex forms a more rigid conformation than the Zn(L′) complex. The left and right halves of Zn(L), which are completely fixed in a different environment upon cooling to 253 K, are exchanging around zinc ion upon heating to 363 K giving averaged signals. In contrast, the Zn(L′) complex is thermally fluctuating even at 253 K without heating. The difference between Zn(L) and Zn(L′) in solution probably comes from a less strained and a strained coordination geometries for Zn(L) and Zn(L′), respectively.

**Figure 10:**
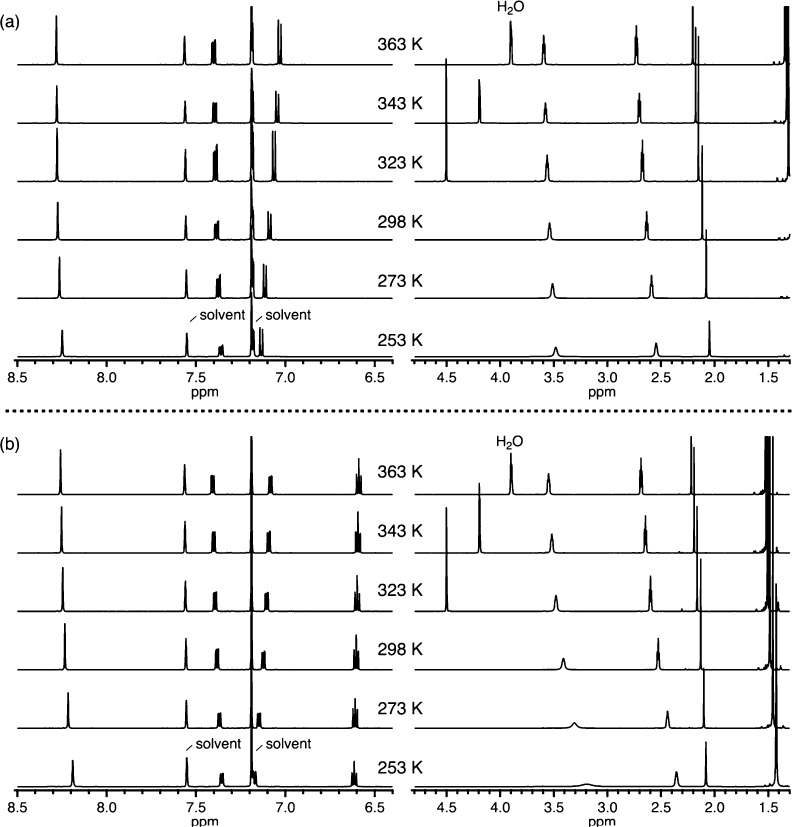
Variable-temperature ^1^H NMR spectra of (a) Zn(L′ ^5-*t*-Bu^) and (b) Zn(L′ ^3-*t*-Bu^) (10 mM, pyridine-*d*_5_).

To confirm the interpretation described above, zinc complexes having different steric bulk, as shown in Chart 4, are investigated with variable-temperature ^1^H NMR spectroscopy. According to the structural models in Figure 8, the incorporation of the bulky *tert*-butyl groups at the 3-positions would disturb the interconversion of the left and right halves of Zn(L) and Zn(L′), while the *tert*-butyl groups at the 5-positions would not have such a steric effect. The Zn(L′ ^5-*t*-Bu^) complex shows a variable-temperature behavior that is identical to that of non-substituted Zn(L′) (Figure 10a). However, the Zn(L′ ^3-*t*-Bu^) complex shows different variable-temperature ^1^H NMR spectra, in which one of the methylene signals is significantly broadened upon cooling (Figure 10b). The *tert*-butyl groups at the 3-positions indeed alter thermal properties of Zn(L′) having ethylene linkers, as expected from the structural model.

**Figure 11:**
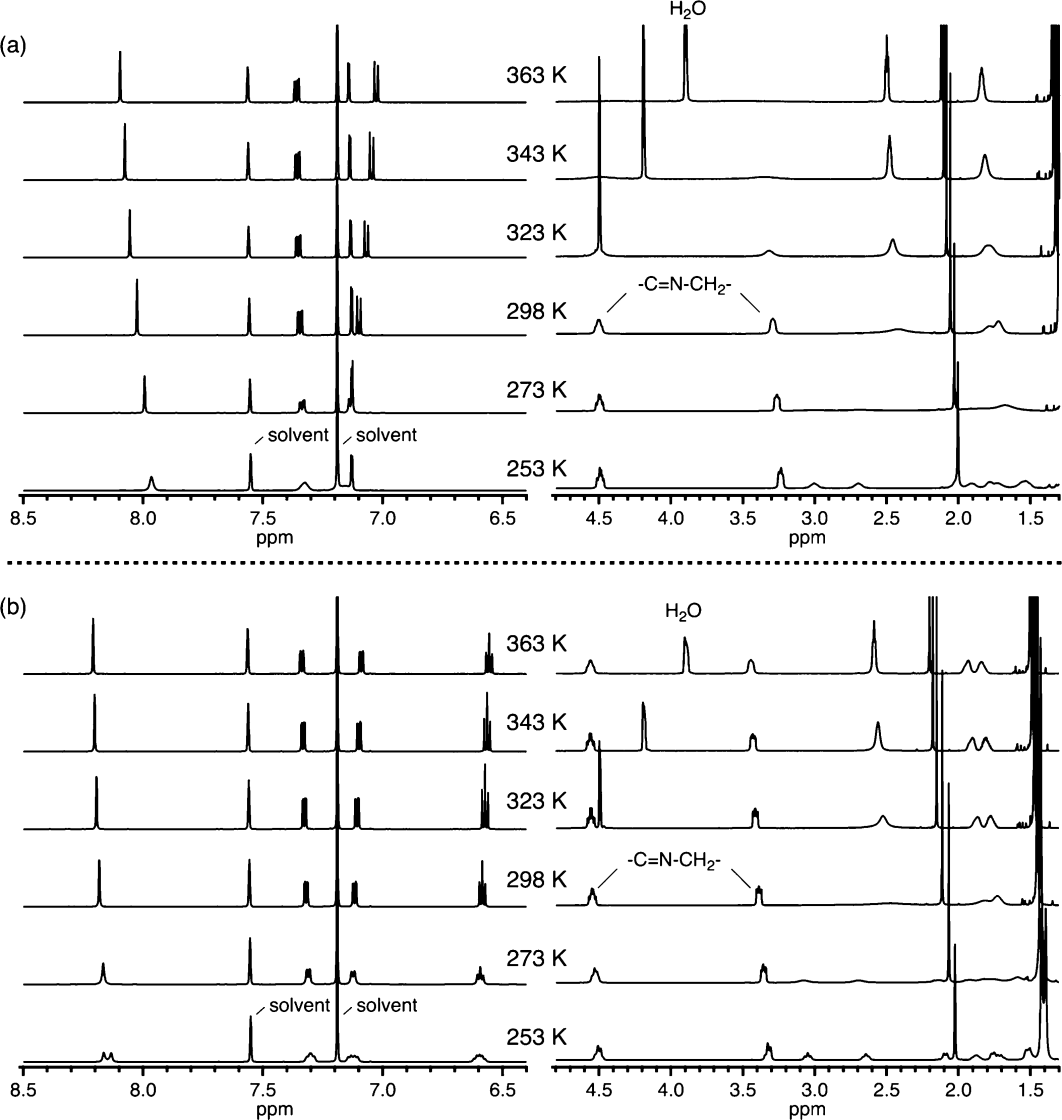
Variable-temperature ^1^H NMR spectra of (a) Zn(L^5-*t*-Bu^) and (b) Zn(L^3-*t*-Bu^) (10 mM, pyridine-*d*_5_).

Figure 11 shows variable-temperature ^1^H NMR spectra of Zn(L^5-*t*-Bu^) and Zn(L^3-*t*-Bu^). While the variable-temperature behavior of Zn(L) and Zn(L^5-*t*-Bu^) is the same (Figure 11a), the Zn(L^3-*t*-Bu^) complex shows significant difference (Figure 11b). The methylene signals adjacent to the azomethine group (−C̅N–CH_2_−), which are broadened out at 323 K in Zn(L^5-*t*-Bu^), are still observed upon heating to 363 K for Zn(L^3-*t*-Bu^). The *tert*-butyl groups at the 3-positions also alter thermal properties of Zn(L) having propylene linkers.

Sterically demanding *tert*-butyl groups at the 3-position suppress thermal fluctuation of a ligand in both Zn(L) and Zn(L′). However, the steric bulk on the phenolate is not so effective as to change the difference in thermal properties between Zn(L) and Zn(L′). Even with the aid of sterically demanding *tert*-butyl groups, the Zn(L′ ^3-*t*-Bu^) complex having ethylene linkers are still fluctuating actively at low temperature of 253 K, which is comparable to the thermal fluctuation of the Zn(L) complex having propylene linkers without any steric bulk at high temperature of 363 K, as indicated from variable-temperature ^1^H NMR spectra in Figures 10b and 9b. This observation shows that the ligand framework (ethylene vs propylene linkers) is of prime importance for thermal fluctuation of a ligand, which determines fluorescence efficiency of flexible π-conjugated ligands like salen ligands.

**Figure 12:**
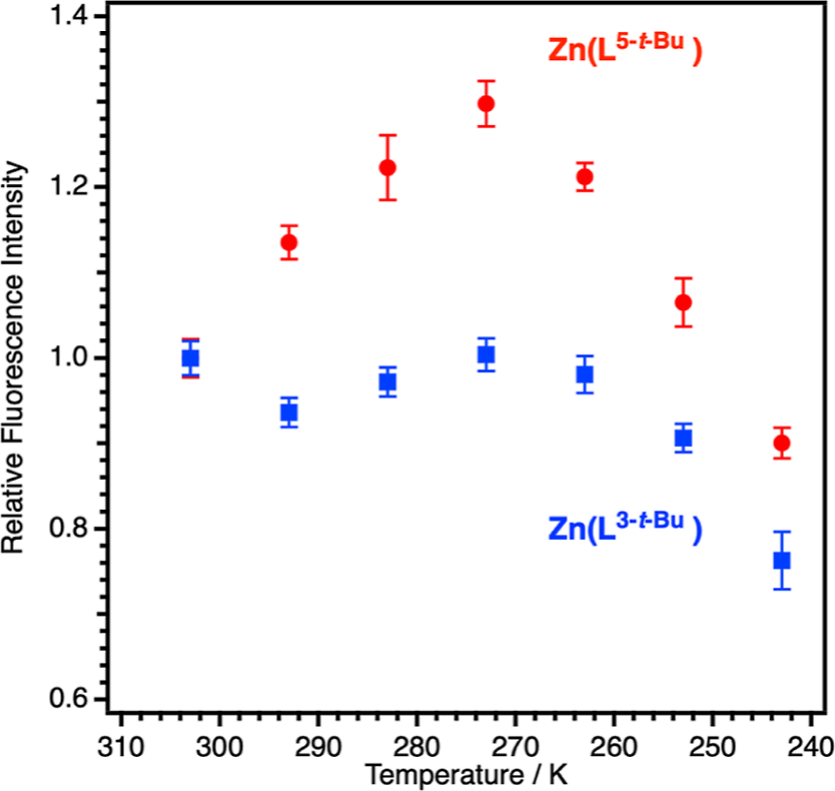
Temperature dependence of fluorescence intensity from thermally fluctuating Zn(L^5-*t*-Bu^) and less-fluctuating Zn(L^3-*t*-Bu^). Each fluorescence measurement was repeated three times to obtain averaged values and standard deviations.

In order to investigate the effect of thermal fluctuation of a ligand on the emission efficiency, variable-temperature fluorescence measurements were carried out for Zn(L^5-*t*-Bu^) and Zn(L^3-*t*-Bu^) bearing *tert*-butyl groups at 5- or 3-positions, respectively, on otherwise the same salen platform. As indicated from variable-temperature ^1^H NMR measurements in Figure 11, the Zn(L^5-*t*-Bu^) complex is thermally more fluctuating than the Zn(L^3-*t*-Bu^). As shown in Figure 12, upon lowering the temperature from 303 K to 273 K, the fluorescence from thermally fluctuating Zn(L^5-*t*-Bu^) is increased by 1.3, while the fluorescence from less-fluctuating Zn(L^3-*t*-Bu^) is not altered in the same temperature range. The observed difference in the temperature dependence of the emission efficiency possibly originates from the difference in thermal fluctuation between Zn(L^5-*t*-Bu^) and Zn(L^3-*t*-Bu^). In the temperature range from 273 to 243 K, the fluorescence intensity is decreased upon cooling for both Zn(L^5-*t*-Bu^) and Zn(L^3-*t*-Bu^) in common.

### Mechanistic Considerations

**Figure 13:**
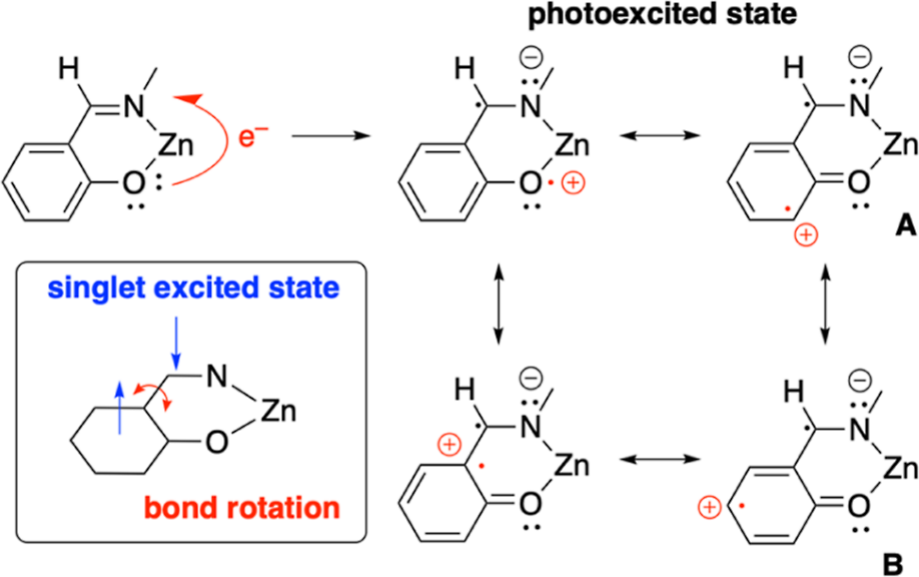
Mechanistic proposal for the photoexcitation of a salen complex.

The present study shows a clear correlation between *E*_ox_ – *E*_red_ and λ_em_, as shown in Figure 4. The *E*_ox_ of salen complexes comes from the one-electron oxidation of the phenolate moiety, as indicated from the detailed studies on phenoxyl radicals from salen complexes by us^39^ and others.^41−44^ The *E*_red_ of salen complexes bearing redox-inactive metal is assigned as arising from one-electron reduction of the azomethine moiety, which is indeed not redox-innocent.^45,46^ Then, the most probable photoexcitation pathway is a transition from the phenolate moiety as an electron donor to the azomethine moiety as an electron acceptor, as shown in Figure 13.

This simple picture is useful to explain some puzzling observations on the photophysical properties of a salen complex described above. One is the regiochemistry of substituents on the salicylidene rings. Incorporation of a methyl group into the 3- or 5-position has positive effect on the fluorescence lifetime and the emission efficiency (Figure 6). This observation is explained by the proposed electronic structure of the photoexcited state, in which the transient radical appears at the 3- and 5-positions. The substituents at the 3- and 5-positions could interact with the photoexcited radical to alter photophysical properties of salen ligands. As indicated from the fluorescence lifetime measurements in Figure 5, the interaction of the photoexcited radical with *tert*-butyl, phenyl, and chloro substituents in addition to methyl gives positive effect on the lifetime of the excited radical, in contrast to the negative impact induced by methoxyl and acetyl substituents.

The other is the difference between the ethylene and propylene bridges in a triamine moiety, which is a determinant factor for the emission efficiency although this moiety has no light-absorbing π-conjugation. The present variable-temperature ^1^H NMR studies reveals that the ligand framework gives significant difference in thermal fluctuation of a ligand around metal ion, which is much more suppressed for the propylene bridge than that for the ethylene bridge. According to the proposed singlet excited state shown in the inset of Figure 13, the bond rotation between the phenolate and the azomethine group is accelerated by thermal fluctuation, resulting in faster quenching of photoexcited radicals that are distributed on the phenolate and the azomethine.

The present result on zinc salen complexes is compared with fluorescence from related compounds. One of the examples is an aluminum salophene complex,^29^ which has an *o*-phenylene diamine linker instead of an ethylene or propylene linker in the present case. Aluminum salophene complexes show *E*_ox_ (1.38–1.42 V) and *E*_red_ (−1.50––1.75 V), which are quite different from the *E*_ox_ (0.14–0.81 V) and *E*_red_ (−2.70––2.91 V) values for the present zinc complexes. This is probably because aluminum salophene complexes with Al^3+^ are cationic in contrast to neutral Zn^2+^ salen complexes. However, the *E*_ox_ – *E*_red_ values fall within the range from 2.92 to 3.16 V with the λ_em_ values of 20,600–20,800 cm^–1^.^29^ According to the correlation in Figure 4, the *E*_ox_ – *E*_red_ values of 2.92 to 3.16 V correspond to the λ_em_ values of 19,500–21,000 cm^–1^, which are in nice agreement with the experimental λ_em_ values. The same correlation for zinc salen and aluminum salophen complexes indicates that the photoexcitation pathway occurs on the common salicylidene moiety without contribution from π-conjugate *o*-phenylene linkers. The suggestion from the present study is that the rigid *o*-phenylene linkers may contribute to high emission efficiency of aluminum salophene complexes by suppressing the thermal fluctuation of a salicylidene chromophore.

Other interesting examples are indium^37^ or aluminum^26^ salen complexes bearing different substituents at the 5-position of the phenolate with *t*-Bu at the 3-position in common. The emission wavelength is altered in the order H < *t*-Bu < Ph < OMe in both indium and aluminum salen complexes, which is the same trend as the present zinc complexes. Indium and aluminum salen complexes bearing *t*-Bu at the 3- and 5-positions show similar emission wavelengths at 484 and 480 nm, as compared with 472 nm for the present zinc complex, although the solvent utilized for the fluorescence measurement is different. According to these observations, the salicylidene moiety determines the emission wavelength, and the difference in metal ions has negligibly small impact. In contrast, the emission efficiency is affected by the choice of metal ions; 36 and 13% for indium and aluminum complexes bearing the same salicylidene chromophore, respectively. The hypothesis from the present study is that the indium ion has some beneficial properties (size or biding affinity) to suppress the thermal fluctuation of a salicylidene chromophore in the salen framework.

## Conclusions

Salicylidene chromophores from metal salen complexes show significantly different emission efficiency, which is not readily predictable from the ligand structure. The present study investigates hidden factors that affect the photophysical properties of the salen framework. One of the new findings is the position of a substituent on the salicylidene ring that affects the fluorescence lifetime in a manner indicative of the photoexcited phenoxyl radical. The most critical factor is thermal fluctuation of a salen ligand arising from the mismatched ligand–metal interaction, which has devastatingly negative impact on the emission efficiency of a salicylidene chromophore.

## Experimental Section

### Instrumentation

^1^H and ^13^C NMR spectra of zinc complexes were measured in a borosilicate glass tube (5 mm OD) on a JNM-ECS400 400 MHz spectrometer (JEOL) or JNM-ECA600 600 MHz NMR spectrometer (JEOL). Variable-temperature ^1^H NMR measurements were carried out using the JNM-ECA600 600 MHz NMR spectrometer (JEOL). ^1^H and ^13^C NMR chemical shifts in CDCl_3_ were referenced to CHCl_3_ (7.240 ppm) and ^13^CDCl_3_ (77.0 ppm). The ^1^H NMR spectrum in pyridine-*d*_5_ were referenced to the residual pyridine signal (7.190 ppm). Cyclic voltammograms were measured with a model 2325 electrochemical analyzer (BAS) using an Ag/Ag^+^ reference electrode, a glassy-carbon working electrode, and a platinum-wire counter electrode. Measurements were carried out for the 0.5 mM solution in electrochemical-grade CH_3_CN containing 0.1 M Bu_4_NOTf at a scan rate of 50 mV s^–1^ at 298 K under an Ar atmosphere unless otherwise noted. The sample solutions were deoxygenated by bubbling Ar gas for 10 min. The *E* values were referenced to the *E*_1/2_ value of ferrocene, which was measured under identical conditions after each measurement. Absorption spectra were recorded in spectroscopy-grade pyridine using a quartz cell (*l* = 0.1 cm) on an Agilent 8453 spectrometer (Agilent Technologies) equipped with a temperature-control cell unit. Absorption spectra were measured for 0.5 mM solutions. Fluorescence spectra were measured in spectroscopy-grade pyridine using a quartz cell (*l* = 1.0 cm) on a Shimazu RF-5300PC spectrofluorometer. Low-temperature fluorescence measurements were carried out using an USP-203 low-temperature chamber (UNISOKU) that is installed into the sample room of the Shimazu RF-5300PC spectrofluorometer. The details are shown in Figure S23. Fluorescence lifetimes were measured using an EasyLife lifetime fluorescence spectrometer (Horiba). The sample solutions for fluorescence measurements were deoxygenated by bubbling Ar gas for 15 min. Fluorescence spectra were measured at 298 K with excitation at λ_ex_ = 390 nm for deoxygenated pyridine solutions with absorbance = 0.01 at 390 nm. Fluorescence lifetimes were measured at 298 K with excitation at λ_ex_ = 375 nm using a 400 nm filter. Fluorescence measurements were carried out three times independently to obtain averaged values and standard deviations. Fluorescence quantum yields were determined using 9,10-diphenylanthracene as a standard (ϕ = 100%^47^). Fluorescence lifetime measurements of anthracene as a standard compound in cyclohexane using the present experimentation gave a value of 5.18 ns, which is in good agreement with the authorized value of 5.3 ns.^48^ Elemental analyses were conducted on a MicroCorder JM10 (J-SCIENCE LAB).

### Materials

Spectroscopy-grade pyridine, toluene, acetone, acetonitrile, and methanol for fluorescence measurements were purchased from Kanto Chemical. Anhydrous solvents were purchased from Kanto Chemical. 2,2-Diamino-*N*-methyldiethylamine and 3,3-diamino-*N*-methyldipropylamine were purchased from Tokyo Chemical Industry. 3,5-Di-*tert*-butylsalicylaldehyde was purchased from Fujifilm Wako Chemical. 3-*tert*-Butylsalicylaldehyde was purchased from Aldrich. Preparations of 3-*tert*-butyl-5-methoxysalicylaldehyde, 3-*tert*-butyl-5-phenylsalicylaldehyde, and 3-*tert*-butyl-5-chlorosalicylaldehyde were described previously.^39^ 3-, 4-, and 5-Methylsalicylaldehydes and salicylaldehyde were purchased from Tokyo Chemical Industry. Preparation of 6-methylsalicylaldehyde was described elsewhere.^44^ Diethylzinc (1.0 M in toluene) was purchased from Tokyo Chemical Industry. Zinc acetate dihydrate was purchased from Kanto Chemical. Other reagents were purchased from Fujifilm Wako Chemical or Kanto Chemical. Membrane filters (Millex-FG, 0.20 μm, 25 mm, hydrophobic PTFE) were purchased from Merck.

### Preparation of 3-*tert*-Butyl-5-acetylsalicylaldehyde

3-*tert*-Butylsalicylaldehyde (5.00 g, 28.1 mmol) was added carefully to the slurry of 1.3 equiv of acetic anhydride (3.50 mL, 37.0 mmol) and AlCl_3_ (11.97 g, 89.8 mmol) at room temperature. After stirring at room temperature for 2 h, the resulting mixture was poured into 1.0 M HCl/H_2_O (100 mL) containing cold ice. The aqueous solution was extracted with CH_2_Cl_2_ (100 mL × 3). The combined organic layer was washed with brine (100 mL) and was then dried over MgSO_4_. After evaporation of the solvent and drying in vacuo, concentrated HCl (5 mL) in acetone was added to the solid, and the resulting solution was refluxed for 3 h. After extracting with CH_2_Cl_2_ (100 mL × 3), the combined organic layer was washed with brine (100 mL) and was dried over MgSO_4_. The title compound was obtained as pale yellow solid (2.52 g, 11.4 mmol) after precipitation from methanol at −20 °C. ^1^H NMR (CDCl_3_, 400 MHz): δ 12.226 (s, 1H), 9.919 (s, 1H), 8.136 (s, 1H), 8.033 (s, 1H), 2.571 (s, 3H), 1.410 (s, 9H).

^13^C NMR (100 MHz, CDCl_3_): δ 196.951, 195.800, 164.915, 138.950, 133.537, 133.339, 128.831, 119.789, 35.009, 29.003, 26.148.

### Preparation of Zn(L′ ^3,5-*t*-Bu^)

3,5-Di-*tert*-butylsalicylaldehyde (4.395 g, 18.8 mmol) was added to the solution of 2,2-diamino-*N*-methyldiethylamine (1.099 g, 9.38 mmol) in EtOH (20 mL). The resulting solution was stirred at 110 °C for 1 h. The solvent was removed by evaporation, and the residue was dried in vacuo at 80 °C for 6 h. The resulting yellow solid was dissolved in anhydrous toluene (40 mL). Then, the toluene solution of Et_2_Zn (1.0 M, 11.3 mL, 11.3 mmol) was carefully added under an Ar atmosphere to the yellow solution. The resulting solution was stirred at 30 °C for 12 h. Then, the reaction solution was poured to saturated NaHCO_3_ aqueous solution (100 mL), and the organic layer was washed with saturated NaHCO_3_ aqueous solution (50 mL). After drying over MgSO_4_, the toluene solution was passed through a pad of celite and then a membrane filter (0.20 μm pore size). The yellow solid after evaporation and drying in vacuo was purified by refluxing in EtOH (80 mL) for 30 min. Filtration of hot EtOH solution gave the title compound (3.259 g, 5.315 mmol) after drying in vacuo at 80 °C for 12 h.

^1^H NMR (CDCl_3_, 600 MHz): δ 8.172 (s, 2H), 7.398 (d, *J* = 2.4 Hz, 2H), 6.927 (d, *J* = 2.4 Hz, 2H), 3.915 (m, 2H), 3.318 (m, 2H), 2.975 (m, 2H), 2.605 (m, 2H), 2.289 (s, 3H), 1.343 (s, 18H), 1.294 (s, 18H).

^13^C NMR (150 MHz, CDCl_3_): δ 171.522, 168.927, 141.524, 134.554, 129.326, 129.010, 117.042, 60.704, 58.885, 42.933, 35.532, 33.799, 31.395, 29.231.

Anal. calcd for C_35_H_53_N_3_O_2_Zn: C, 68.56; H, 8.71; N, 6.85; found: C, 68.51; H, 8.81; N, 7.09.

HRMS calcd for C_35_H_53_N_3_O_2_Zn H^+^*m*/*z* 612.3507; found, 612.3490. The low-resolution mass spectrum in a wide *m*/*z* range is shown in Figure S1.

### Preparations of Zn(L^3,5-*t*-Bu^)

3,5-Di-*tert*-butylsalicylaldehyde (798 mg, 3.40 mmol) was added to the solution of 3,3-diamino-*N*-methyldipropylamine (247 mg, 1.70 mmol) in EtOH (8 mL). The resulting solution was stirred at 110 °C for 30 min. Then, triethylamine (2.37 mL) and zinc acetate dihydrate (746 mg, 3.40 mmol) were added to the solution. The resulting solution was heated at 110 °C for 1 h. After cooling, the solvent was removed by evaporation, and the residue was dried in vacuo. The residue dissolved in CH_2_Cl_2_ (50 mL) was washed with saturated NaHCO_3_ (50 mL × 3). After drying over MgSO_4_, the solution was passed through a pad of celite and then a membrane filter (Millex-FG, 0.20 μm, 25 mm, hydrophobic PTFE). The solvent was removed by evaporation, and the residue was dried in vacuo. The title compound (922 mg, 1.44 mmol) was obtained by precipitation from hot ethanol.

^1^H NMR (CDCl_3_, 400 MHz): δ 8.059 (s, 2H), 7.268 (d, *J* = 2.8 Hz, 2H), 6.849 (d, *J* = 2.8 Hz, 2H), 4.576 (m, 2H), 3.395 (m, 2H), 2.688 (broad), 2.266 (s, 3H), 2.006 (broad), 1.909 (broad), 1.365 (s, 18H), 1.251 (s, 18H).

^13^C NMR (100 MHz, CDCl_3_): δ 169.406, 167.661, 140.440, 133.095, 128.214, 127.755, 117.518, 38.486, 35.334, 33.697, 31.478, 29.412.

Anal. calcd for C_37_H_57_N_3_O_2_Zn: C, 69.30; H, 8.96; N, 6.55; found: C, 69.24; H, 8.89; N, 6.44.

HRMS calcd for C_37_H_57_N_3_O_2_Zn H^+^*m*/*z* 640.3820; found, 640.3801. The low-resolution mass spectrum in a wide *m*/*z* range is shown in Figure S3.

### Preparations of Zn(L ^3-*t*-Bu,5-MeO^)

The title compound was prepared according to the procedure as described for Zn(L^3,5-*t*-Bu^). The title compound (44.4 mg, 0.075 mmol) was obtained from 3-*tert*-butyl-5-methoxysalicylaldehyde (461 mg, 2.21 mmol) and 3,3-diamino-*N*-methyldipropylamine (160 mg, 1.10 mmol), after precipitation from diethyl ether at −20 °C.

^1^H NMR (CDCl_3_, 400 MHz): δ 8.016 (s, 2H), 6.911 (d, *J* = 3.2 Hz, 2H), 6.398 (d, *J* = 3.2 Hz, 2H), 4.549 (m, 2H), 3.701 (s, 6H), 3.400 (m, 2H), 2.703 (broad), 2.269 (s, 3H), 2.015 (broad), 1.922 (broad), 1.337 (s, 18H).

^13^C NMR (100 MHz, CDCl_3_): δ 167.085, 166.982, 146.559, 142.822, 120.479, 116.776, 113.016, 56.140, 38.466, 35.258, 29.175.

Anal. calcd for C_31_H_45_N_3_O_4_Zn: C, 63.21; H, 7.70; N, 7.13; found: C, 63.13; H, 7.69; N, 7.10.

HRMS calcd for C_31_H_45_N_3_O_4_Zn H^+^*m*/*z* 588.2780; found, 588.2761. The low-resolution mass spectrum in a wide *m*/*z* range is shown in Figure S2.

### Preparations of Zn(L ^3-*t*-Bu,5-Ph^)

The title compound was prepared according to the procedure as described for Zn(L^3,5-*t*-Bu^). The title compound (22.1 mg, 0.0324 mmol) was obtained from 3-*tert*-butyl-5-phenylsalicylaldehyde (216.8 mg, 0.852 mmol) and 3,3-diamino-*N*-methyldipropylamine (61.9 mg, 0.426 mmol), after precipitation from diethyl ether at −20 °C.

^1^H NMR (CDCl_3_, 400 MHz): δ 8.162 (s, 2H), 7.506 (d, *J* = 7.3 Hz, 4H), 7.482 (d, *J* = 2.8 Hz, 4H), 7.352 (dd, *J* = 7.3, 7.3 Hz, 4H), 7.204 (d, *J* = 7.3 Hz, 2H), 7.179 (d, *J* = 2.8 Hz, 2H), 4.604 (m, 2H), 3.477 (m, 2H), 2.771 (broad), 2.321 (s, 3H), 2.068 (broad), 1.976 (broad), 1.402 (s, 18H).

^13^C NMR (100 MHz, CDCl_3_): δ 171.104, 167.692, 142.062, 141.633, 131.198, 128.979, 128.489, 126.148, 125.353, 124.480, 118.819, 38.502, 35.273, 29.367.

Anal. calcd for C_41_H_49_N_3_O_2_Zn: C, 72.29; H, 7.25; N, 6.17; found: C, 72.27; H, 7.31; N, 6.18.

HRMS calcd for C_41_H_49_N_3_O_2_Zn H^+^*m*/*z* 680.3194; found, 680.3183. The low-resolution mass spectrum in a wide *m*/*z* range is shown in Figure S4.

### Preparations of Zn(L^3-*t*-Bu,5-Cl^)

The title compound was prepared according to the procedure as described for Zn(L^3,5-*t*-Bu^). The title compound (483.0 mg, 0.805 mmol) was obtained from 3-*tert*-butyl-5-chlorosalicylaldehyde (490.8 mg, 2.31 mmol) and 3,3-diamino-*N*-methyldipropylamine (167.6 mg, 1.15 mmol), after purification by sonification of the suspension in diethyl ether.

^1^H NMR (CDCl_3_, 400 MHz): δ 7.962 (s, 2H), 7.079 (d, *J* = 3.0 Hz, 2H), 6.868 (d, *J* = 3.0 Hz, 2H), 4.490 (m, 2H), 3.416 (m, 2H), 2.275 (s, 3H), 1.944 (broad), 1.296 (s, 18H).

^13^C NMR (100 MHz, CDCl_3_): δ 169.865, 166.605, 143.500, 130.984, 129.958, 119.156, 115.805, 38.471, 35.273, 29.030.

Anal. calcd for C_29_H_39_Cl_2_N_3_O_2_Zn (H_2_O)_0.1_: C, 58.08; H, 6.59; N, 7.01; found: C, 57.99; H, 6.61; N, 6.99.

HRMS calcd for C_29_H_39_Cl_2_N_3_O_2_Zn H^+^*m*/*z* 596.1789; found, 596.1772. The low-resolution mass spectrum in a wide *m*/*z* range is shown in Figure S5.

### Preparations of Zn(L ^3-*t*-Bu,5-MeCO^)

The title compound was prepared according to the procedure as described for Zn(L^3,5-*t*-Bu^). The title compound (156.1 mg, 0.252 mmol) was obtained from 3-*tert*-butyl-5-acetylsalicylaldehyde (128.3 mg, 0.582 mmol) and 3,3-diamino-*N*-methyldipropylamine (42.3 mg, 0.291 mmol), after precipitation by sonification of the suspension in diethyl ether.

^1^H NMR (CDCl_3_, 400 MHz): δ 8.138 (s, 2H), 7.840 (d, *J* = 2.3 Hz, 2H), 7.675 (d, *J* = 2.3 Hz, 2H), 4.517 (m, 2H), 3.512 (m, 2H), 3.289 (broad), 3.049 (broad), 2.460 (s, 6H), 2.322 (s, 3H), 1.971 (broad), 1.318 (s, 18H).

^13^C NMR (100 MHz, CDCl_3_): δ 195.984, 175.526, 167.416, 141.343, 136.385, 129.499, 122.262, 118.192, 38.532, 35.105, 29.168, 25.755.

Anal. calcd for C_33_H_45_N_3_O_4_Zn (H_2_O)_0.4_: C, 63.90; H, 7.44; N, 6.77; found: C, 63.98; H, 7.36; N, 6.69.

HRMS calcd for C_33_H_45_N_3_O_4_Zn H^+^*m*/*z* 612.2780; found, 612.2764. The low-resolution mass spectrum in a wide *m*/*z* range is shown in Figure S6.

### Preparations of Zn(L)

The title compound was prepared according to the procedure as described for Zn(L^3,5-*t*-Bu^). The title compound (475.1 mg, 1.11 mmol) was obtained from salicylaldehyde (587.0 mg, 4.80 mmol) and 3,3-diamino-*N*-methyldipropylamine (349.1 mg, 2.40 mmol), after precipitation from dichloromethane (2.0 mL) and hexane (6.0 mL).

^1^H NMR (CDCl_3_, 400 MHz): δ 8.072 (s, 2H), 7.147 (ddd, *J* = 8.7, 7.8, 1.8 Hz, 2H), 7.031 (dd, *J* = 7.8, 1.8 Hz, 2H), 6.734 (d, *J* = 8.7 Hz, 2H), 6.459 (dd, *J* = 8.7, 7.8 Hz, 2H), 4.442 (s, 2H), 3.440 (s, 2H), 2.592 (s, 4H), 2.188 (s, 3H), 1.990 (s, 2H), 1.901 (s, 2H).

^13^C NMR (100 MHz, CDCl_3_): δ 171.747, 168.105, 134.656, 133.340, 123.134, 118.926, 113.004, 58.485, 38.425, 26.582.

Anal. calcd for C_21_H_25_N_3_O_2_Zn (H_2_O)_0.5_: C, 59.23; H, 6.15; N, 9.87; found: C, 59.20; H, 5.97; N, 9.77.

HRMS calcd for C_21_H_25_N_3_O_2_Zn H^+^*m*/*z* 416.1316; found, 416.1302. The low-resolution mass spectrum in a wide *m*/*z* range is shown in Figure S7.

### Preparations of Zn(L^3-Me^)

The title compound was prepared according to the procedure as described for Zn(L^3,5-*t*-Bu^). The title compound (548.6 mg, 1.22 mmol) was obtained from 3-methylsalicylaldehyde (369.3 mg, 2.71 mmol) and 3,3-diamino-*N*-methyldipropylamine (197.0 mg, 1.36 mmol), after precipitation from dichloromethane (8.0 mL) and hexane (30 mL).

^1^H NMR (CDCl_3_, 400 MHz): δ 8.064 (s, 2H), 7.088 (d, *J* = 6.9 Hz, 2H), 6.921 (dd, *J* = 7.8, 1.8 Hz, 2H), 6.369 (dd, *J* = 7.8, 6.9 Hz, 2H), 4.543 (m, 2H), 3.419 (m, 2H), 2.598 (broad), 2.178 (s, 3H), 2.122 (s, 6H), 2.006 (broad), 1.886 (broad).

^13^C NMR (100 MHz, CDCl_3_): δ 170.538, 167.202, 132.942, 132.101, 130.968, 117.641, 111.918, 58.149, 38.257, 26.505, 16.881.

Anal. calcd for C_23_H_29_N_3_O_2_Zn (H_2_O)_0.2_: C, 61.60; H, 6.61; N, 9.37; found: C, 61.53; H, 6.57; N, 9.33.

HRMS calcd for C_23_H_29_N_3_O_2_Zn H^+^*m*/*z* 444.1629; found, 444.1627. The low-resolution mass spectrum in a wide *m*/*z* range is shown in Figure S8.

### Preparations of Zn(L^4-Me^)

The title compound was prepared according to the procedure as described for Zn(L^3,5-*t*-Bu^). The title compound (418.7 mg, 0.941 mmol) was obtained from 4-methylsalicylaldehyde (387.7 mg, 2.85 mmol) and 3,3-diamino-*N*-methyldipropylamine (206.8 mg, 1.42 mmol), after sonification of the suspension in diethyl ether.

^1^H NMR (CDCl_3_, 400 MHz): δ 8.019 (s, 2H), 6.913 (d, *J* = 7.9 Hz, 2H), 6.565 (d, *J* = 1.2 Hz, 2H), 6.286 (dd, *J* = 7.9, 1.2 Hz, 2H), 4.417 (m, 2H), 3.403 (m, 2H), 2.571 (broad), 2.186 (s, 6H), 2.170 (s, 3H), 1.961 (broad), 1.900 (broad).

^13^C NMR (100 MHz, CDCl_3_): δ 171.701, 167.661, 144.036, 134.518, 123.210, 116.723, 114.626, 58.501, 38.379, 26.643, 21.624.

Anal. calcd for C_23_H_29_N_3_O_2_Zn: C, 62.10; H, 6.57; N, 9.45; found: C, 62.05; H, 6.60; N, 9.40.

HRMS calcd for C_23_H_29_N_3_O_2_Zn H^+^*m*/*z* 444.1629; found, 444.1625. The low-resolution mass spectrum in a wide *m*/*z* range is shown in Figure S9.

### Preparations of Zn(L^5-Me^)

The title compound was prepared according to the procedure as described for Zn(L^3,5-*t*-Bu^). The title compound (391.9 mg, 0.881 mmol) was obtained from 5-methylsalicylaldehyde (372.1 mg, 2.73 mmol) and 3,3-diamino-*N*-methyldipropylamine (198.5 mg, 1.37 mmol), after sonification of the suspension in diethyl ether.

^1^H NMR (CDCl_3_, 400 MHz): δ 8.019 (s, 2H), 6.972 (dd, *J* = 8.6, 2.3 Hz, 2H), 6.804 (d, *J* = 2.3 Hz, 2H), 6.664 (d, *J* = 8.6 Hz, 2H), 4.427 (m, 2H), 3.406 (m, 2H), 2.556 (broad), 2.169 (s, 6H), 2.155 (s, 3H), 1.970 (broad), 1.871 (broad).

^13^C NMR (100 MHz, CDCl_3_): δ 169.819, 168.120, 134.687, 134.075, 122.950, 121.451, 118.222, 58.164, 38.379, 26.643, 20.033.

Anal. calcd for C_23_H_29_N_3_O_2_Zn: C, 62.10; H, 6.57; N, 9.45; found: C, 62.07; H, 6.65; N, 9.35.

HRMS calcd for C_23_H_29_N_3_O_2_Zn H^+^*m*/*z* 444.1629; found, 444.1614. The low-resolution mass spectrum in a wide *m*/*z* range is shown in Figure S10.

### Preparations of Zn(L^6-Me^)

The title compound was prepared according to the procedure as described for Zn(L^3,5-*t*-Bu^). The title compound (79.0 mg, 0.177 mmol) was obtained from 6-methylsalicylaldehyde (155.4 mg, 1.14 mmol) and 3,3-diamino-*N*-methyldipropylamine (82.9 mg, 0.57 mmol), after sonification of the suspension in diethyl ether.

^1^H NMR (CDCl_3_, 400 MHz): δ 8.496 (s, 2H), 7.003 (dd, *J* = 8.6, 7.2 Hz, 2H), 6.590 (d, *J* = 8.6 Hz, 2H), 6.230 (dd, *J* = 7.2 Hz, 2H), 4.494 (m, 2H), 3.426 (m, 2H), 2.604 (broad), 2.366 (s, 6H), 2.198 (s, 3H), 1.955 (broad).

^13^C NMR (100 MHz, CDCl_3_): δ 172.695, 164.601, 140.226, 133.233, 122.139, 117.289, 115.330, 58.271, 38.349, 26.811, 20.079.

Anal. calcd for C_23_H_29_N_3_O_2_Zn (H_2_O)_0.1_: C, 61.85; H, 6.59; N, 9.41; found: C, 61.87; H, 6.64; N, 9.33.

HRMS calcd for C_23_H_29_N_3_O_2_Zn H^+^*m*/*z* 444.1629; found, 444.1614. The low-resolution mass spectrum in a wide *m*/*z* range is shown in Figure S11.

### Preparations of Zn(L^3-*t*-Bu^)

The title compound was prepared according to the procedure as described for Zn(L^3,5-*t*-Bu^). The title compound (510.4 mg, 0.945 mmol) was obtained from 3-*tert*-butylsalicylaldehyde (450.3 mg, 2.53 mmol) and 3,3-diamino-*N*-methyldipropylamine (183.5 mg, 1.26 mmol), after precipitation from dichloromethane (5 mL) and hexane (30 mL).

^1^H NMR (CDCl_3_, 400 MHz): δ 8.059 (s, 2H), 7.182 (dd, *J* = 7.4, 1.8 Hz, 2H), 6.923 (dd, *J* = 7.8, 1.8 Hz, 2H), 6.380 (dd, *J* = 7.8, 7.4 Hz, 2H), 4,559 (m, 2H), 3.416 (m, 2H), 2.716 (broad), 2.283 (s, 3H), 2.030 (broad), 1.938 (broad), 1.339 (s, 18H).

^13^C NMR (100 MHz, CDCl_3_): δ 171.386, 167.522, 141.235, 132.956, 129.552, 118.915, 111.532, 38.454, 35.051, 29.267.

Anal. calcd for C_29_H_41_N_3_O_2_Zn (H_2_O)_0.6_: C, 64.52; H, 7.88; N, 7.88; found: C, 64.40; H, 7.65; N, 7.77.

HRMS calcd for C_29_H_41_N_3_O_2_Zn H^+^*m*/*z* 528.2568; found, 528.2575. The low-resolution mass spectrum in a wide *m*/*z* range is shown in Figure S12.

### Preparations of Zn(L^5-*t*-Bu^)

The title compound was prepared according to the procedure as described for Zn(L^3,5-*t*-Bu^). The title compound (216.6 mg, 0.403 mmol) was obtained from 5-*tert*-butylsalicylaldehyde (267.3 mg, 1.50 mmol) and 3,3-diamino-*N*-methyldipropylamine (108.9 mg, 0.750 mmol), after precipitation from dichloromethane (2 mL) and hexane (30 mL).

^1^H NMR (CDCl_3_, 400 MHz): δ 8.074 (s, 2H), 7.182 (dd, *J* = 7.4, 1.8 Hz, 2H), 6.923 (dd, *J* = 7.8, 1.8 Hz, 2H), 6.380 (dd, *J* = 7.8, 7.4 Hz, 2H), 4,559 (m, 2H), 3.416 (m, 2H), 2.716 (broad), 2.283 (s, 3H), 2.030 (broad), 1.938 (broad), 1.339 (s, 18H).

^13^C NMR (100 MHz, CDCl_3_): δ 169.696, 168.419, 135.175, 131.196, 130.322, 122.664, 117.639, 58.268, 38.581, 33.464, 31.417, 26.541.

Anal. calcd for C_29_H_41_N_3_O_2_Zn (H_2_O)_0.5_: C, 64.74; H, 7.87; N, 7.81; found: C, 64.84; H, 7.75; N, 7.82.

HRMS calcd for C_29_H_41_N_3_O_2_Zn H^+^*m*/*z* 528.2568; found, 528.2559. The low-resolution mass spectrum in a wide *m*/*z* range is shown in Figure S13.

## Supporting Information Available

Full range of mass spectra of zinc complexes, photophysical data of Zn(L^3,5-*t*-Bu^) in different solvents, 2D NMR experiments to assign ^1^H NMR signals, details of low-temperature fluorescence measurements, and ^1^H and ^13^C NMR spectra of zinc complexes (PDF)
