# Supplementary material for: Variation of the Emission Efficiency and Wavelength from Fluorescent Zinc Salen Complexes upon Systematic Structural Modifications
Source: ACS Omega. 2022 Aug 17;7(34):30642–54. doi: 10.1021/acsomega.2c04714 (PMC9435038; doi:10.1021/acsomega.2c04714)
Supplement: Supplementary file 1 — ao2c04714_si_001.pdf [file ao2c04714_si_001.pdf]

Supporting Information for

"Variation of the Emission Efficiency and Wavelength from Fluorescent Zinc Salen Complexes upon Systematic Structural Modifications"

Takuya Kurahashi*

Department of Nutrition Science, Faculty of Nursing and Nutrition, University of Nagasaki, Siebold, Manabino, Nagayo-cho, Nishi-Sonogi-gun, Nagasaki 851-2195, Japan

Content:

Full range of mass spectra of zinc complexes (Figure S1 – S13) ································ S2 – S14

Photophysical data of Zn($L^{3,5\text{-}t\text{-}Bu}$) in different solvent (Table S1)······································· S15

2D NMR experiments to assign ^{1}H NMR signals (Figure S14 – S16) ························· S16 – S18

Details of low-temperature fluorescence measurements (Figure S17) ································· S19

^{1}H and ^{13}C NMR spectra of zinc complexes (Figure S18 – S43) ·································S20 – S45

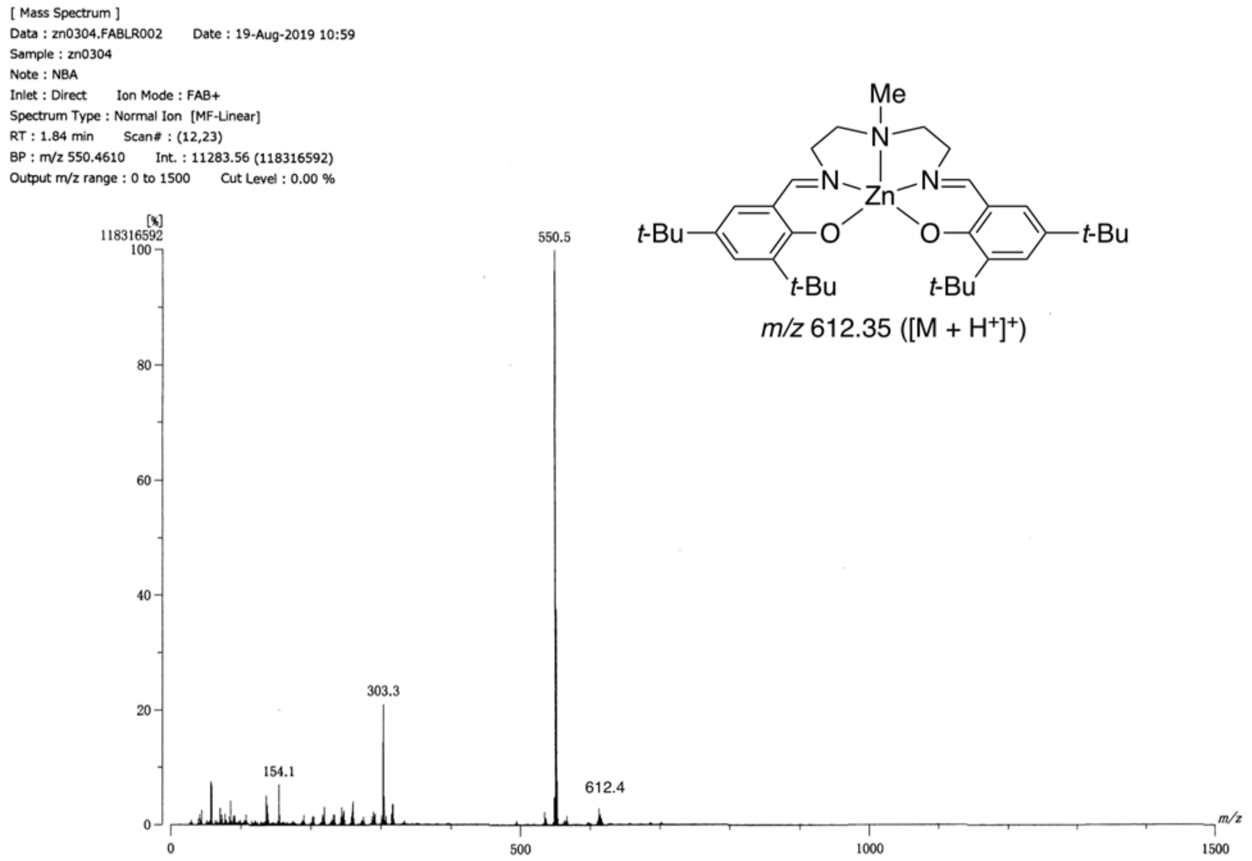

Figure S1. Mass spectrum of Zn(L' $^{3,5\text{-}t\text{-Bu}}$).

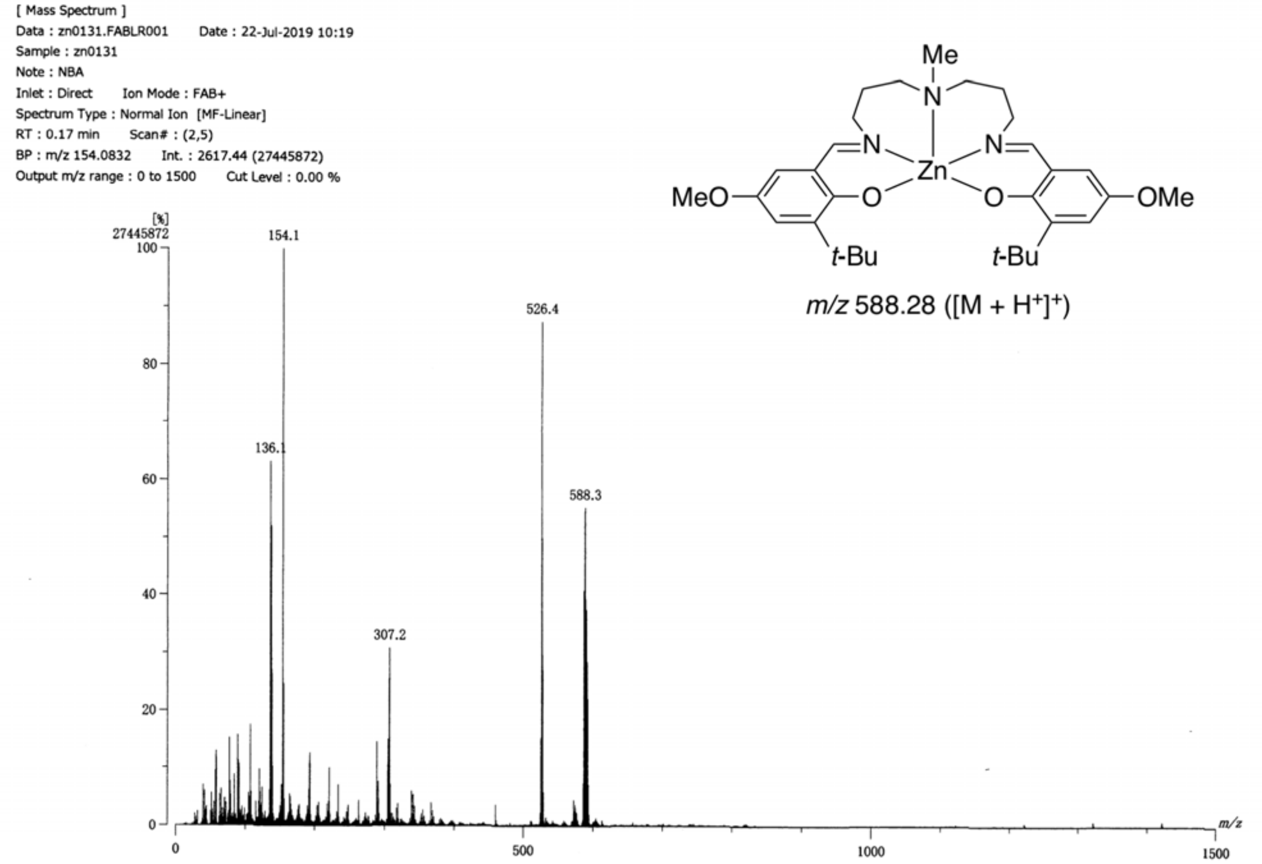

Figure S2. Mass spectrum of Zn($L^{3\text{-}t\text{-Bu},5\text{-MeO}}$).

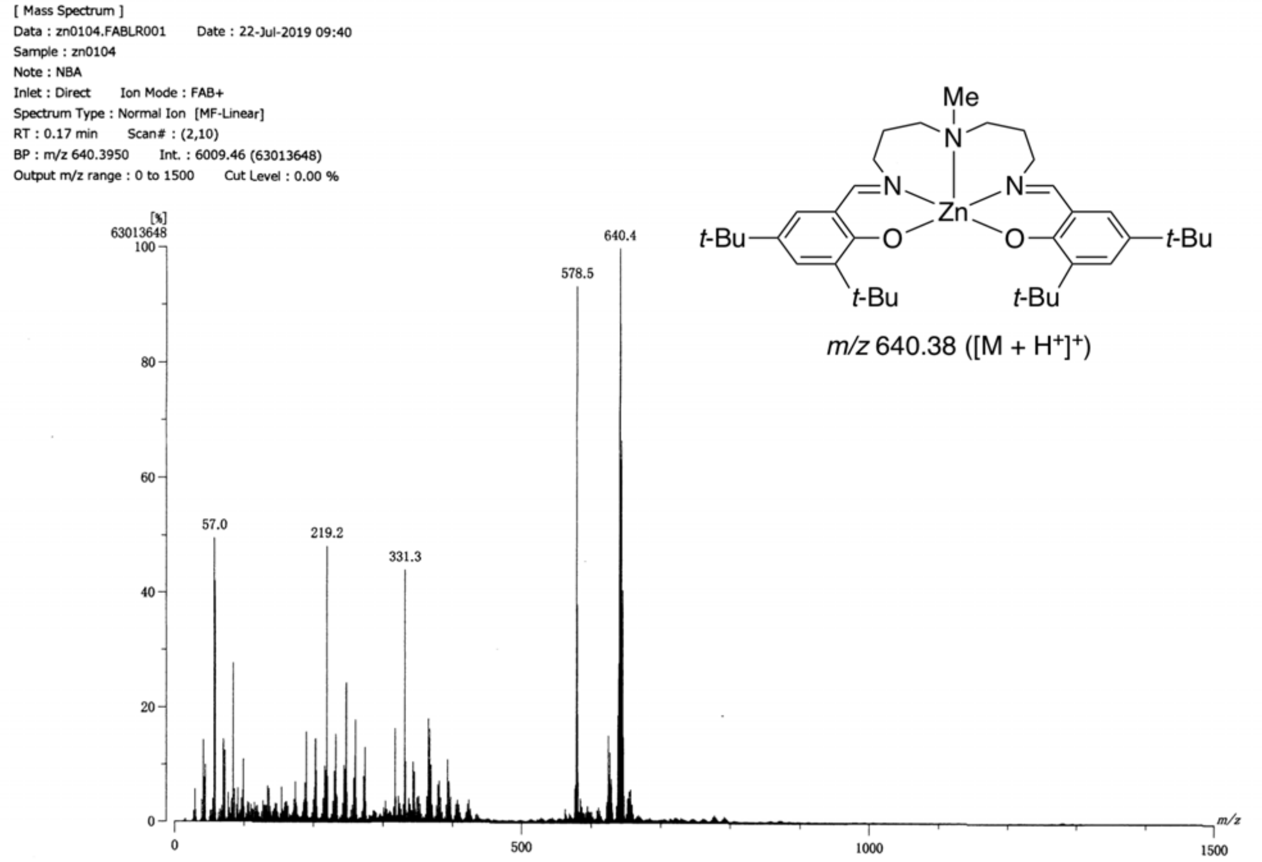

Figure S3. Mass spectrum of Zn($L^{3,5\text{-}t\text{-Bu}}$).

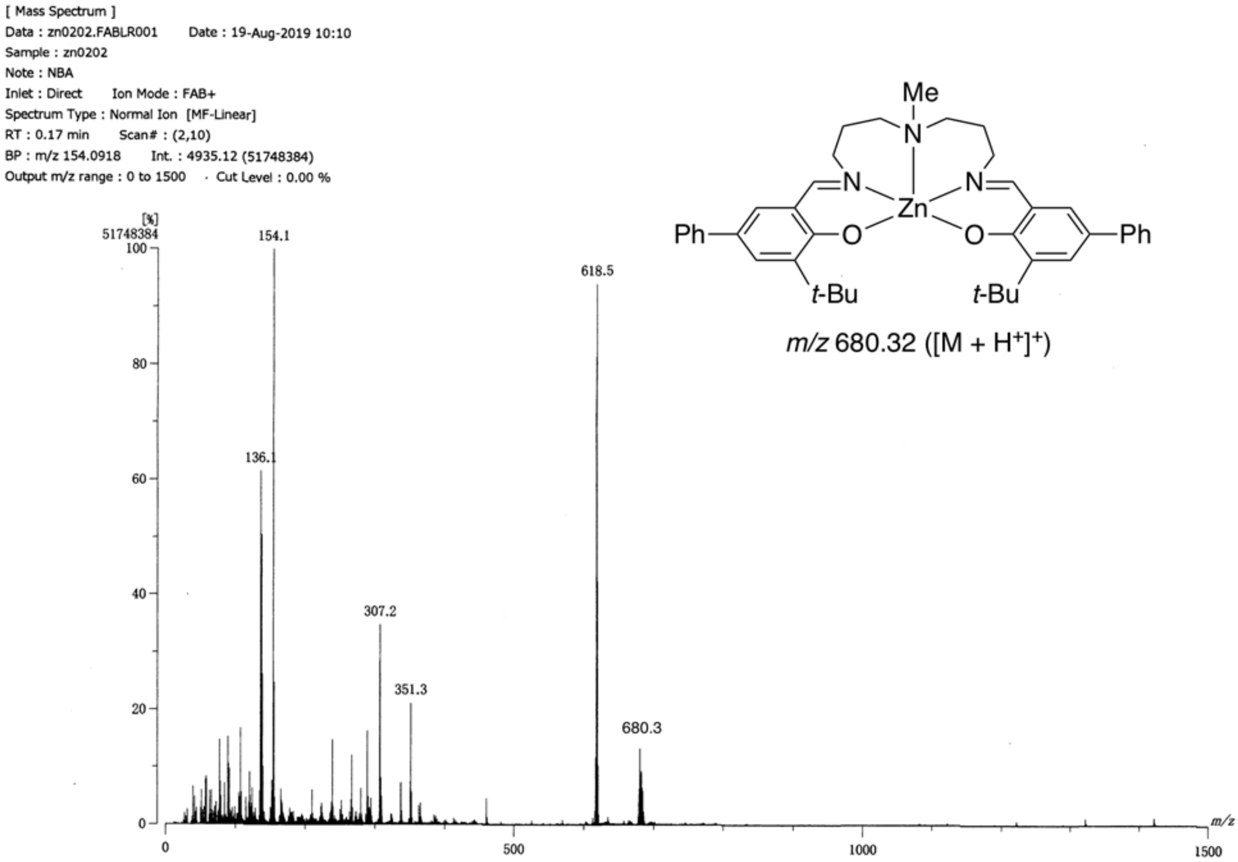

Figure S4. Mass spectrum of Zn($L^{3\text{-}t\text{-Bu},5\text{-Ph}}$).

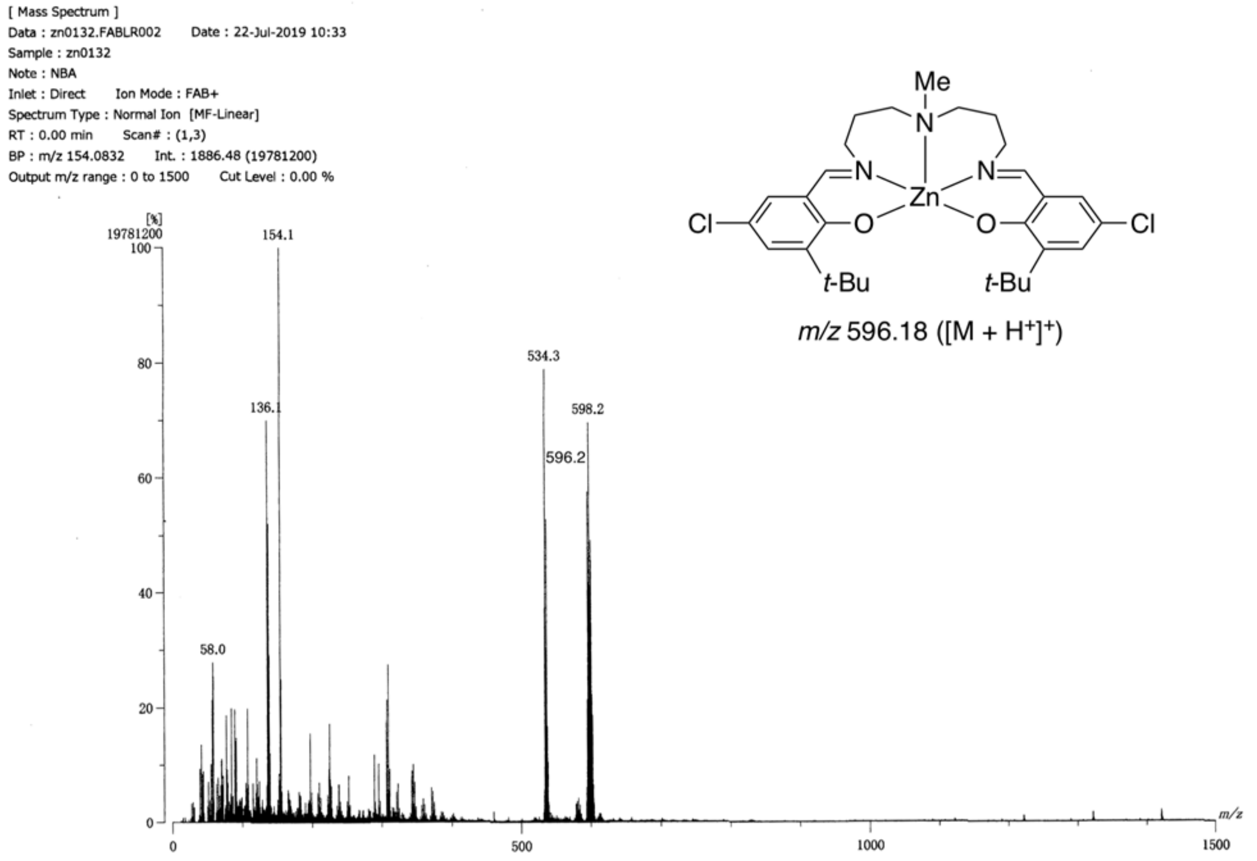

Figure S5. Mass spectrum of Zn($L^{3\text{-}t\text{-Bu},5\text{-Cl}}$).

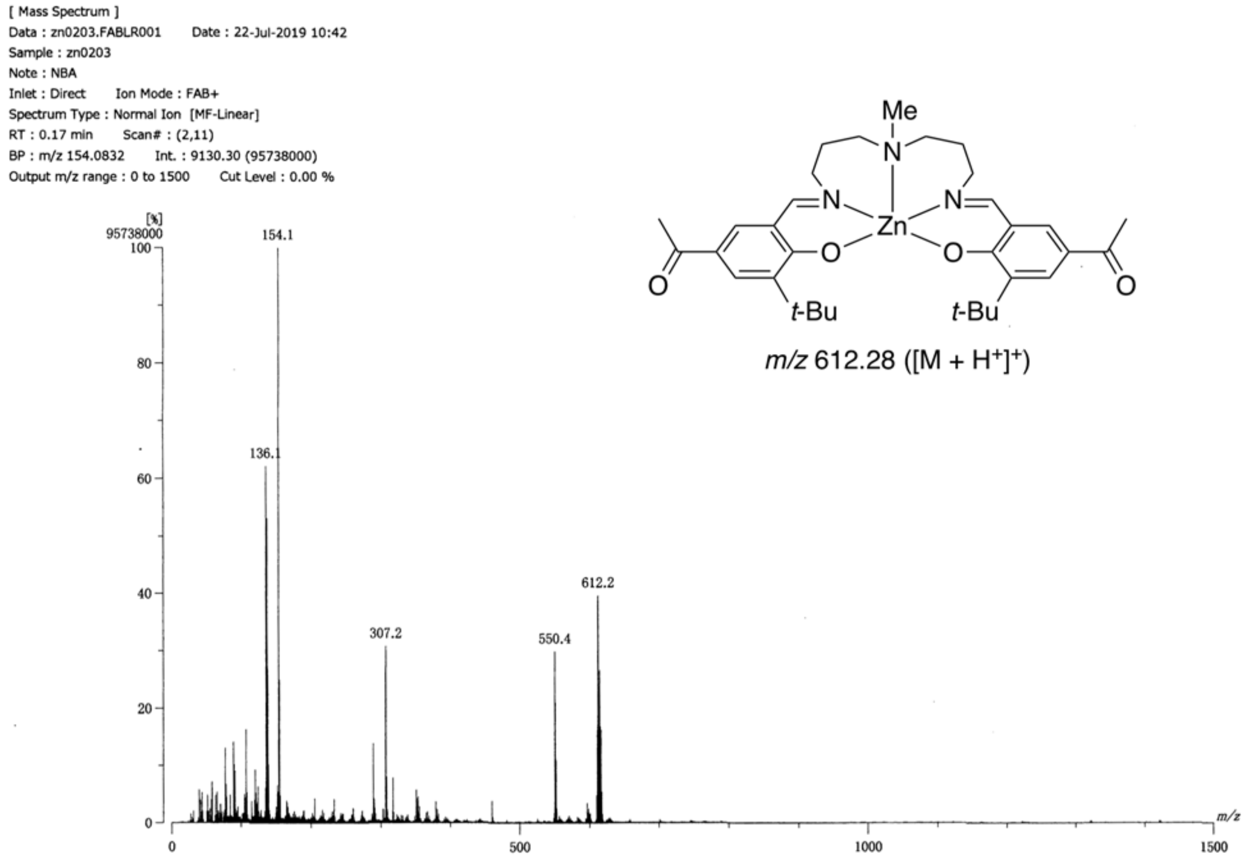

Figure S6. Mass spectrum of Zn($L^{3\text{-}t\text{-Bu},5\text{-MeCO}}$).

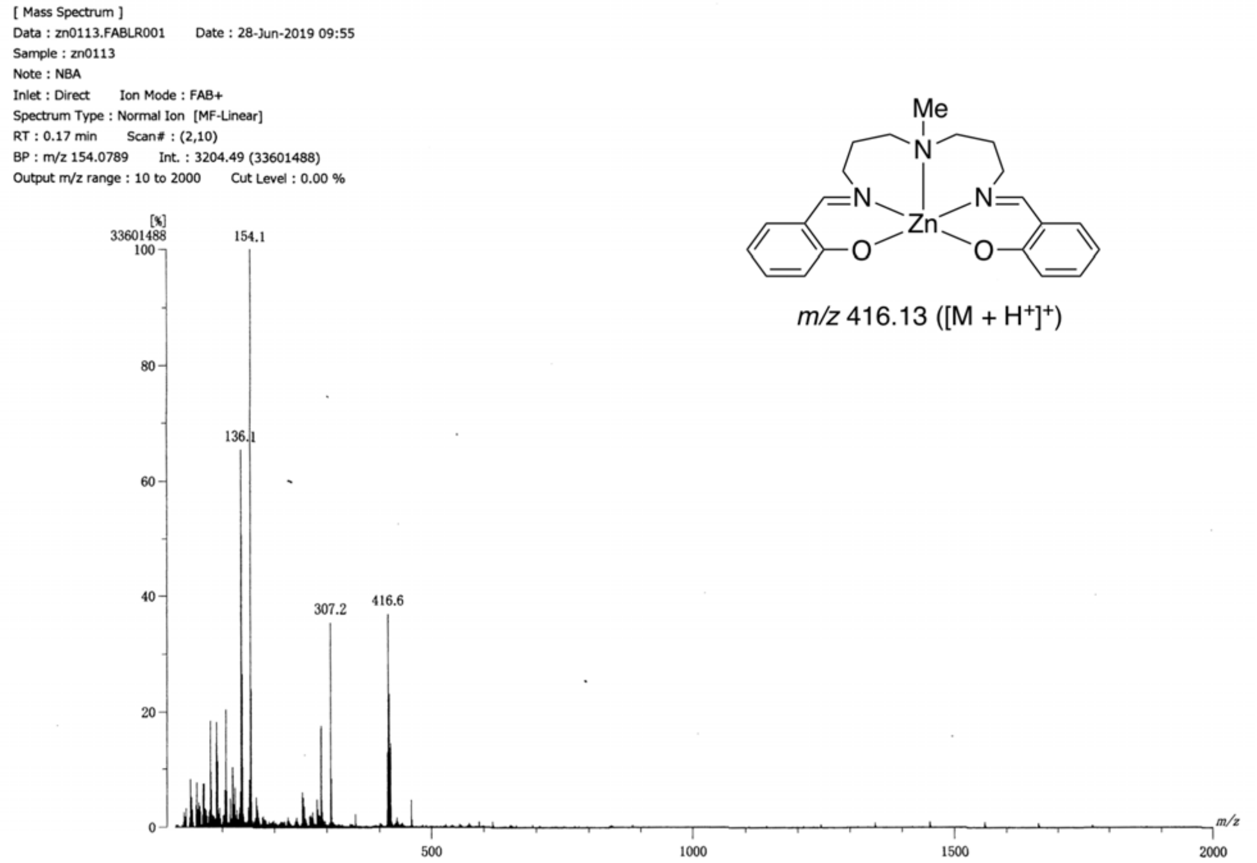

Figure S7. Mass spectrum of Zn(L).

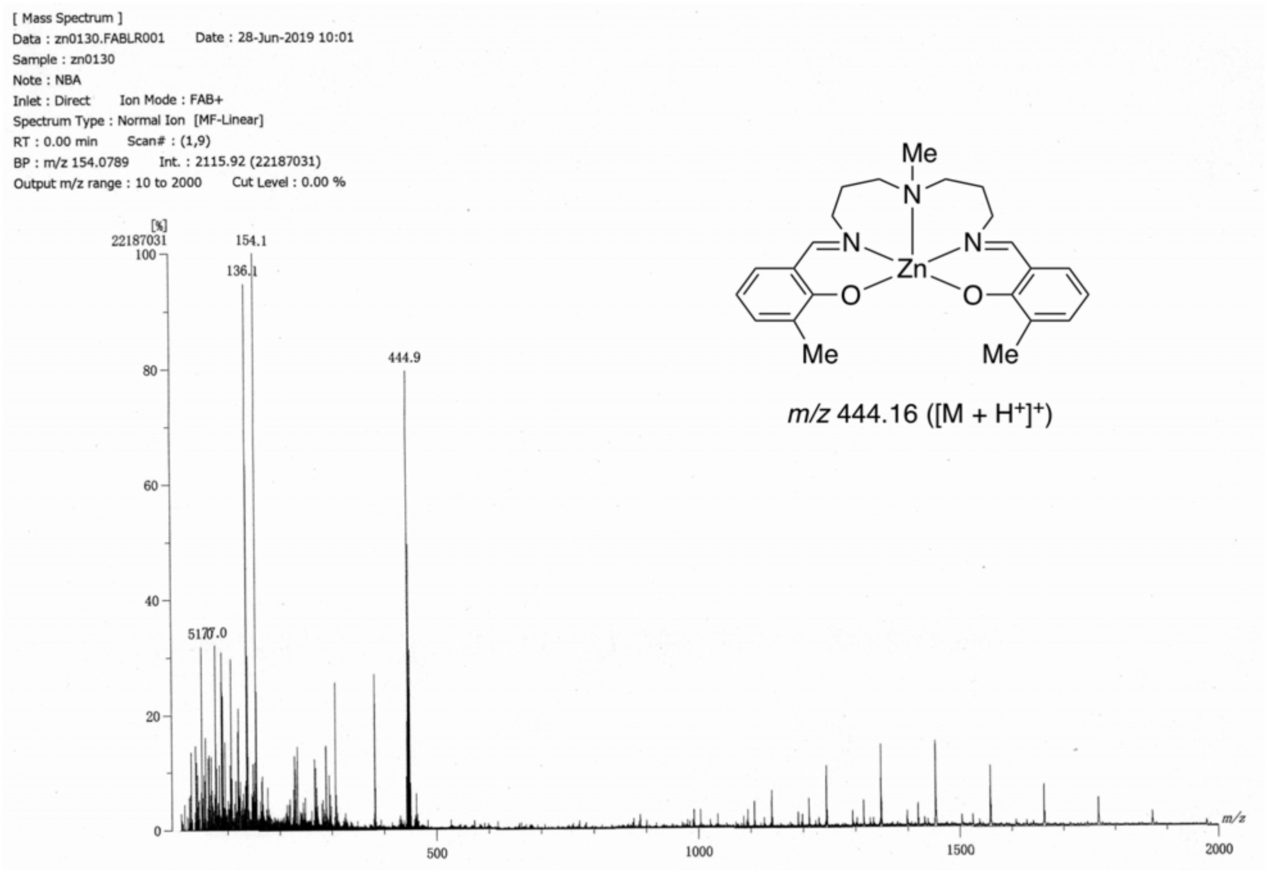

Figure S8. Mass spectrum of Zn($L^{3\text{-}Me}$).

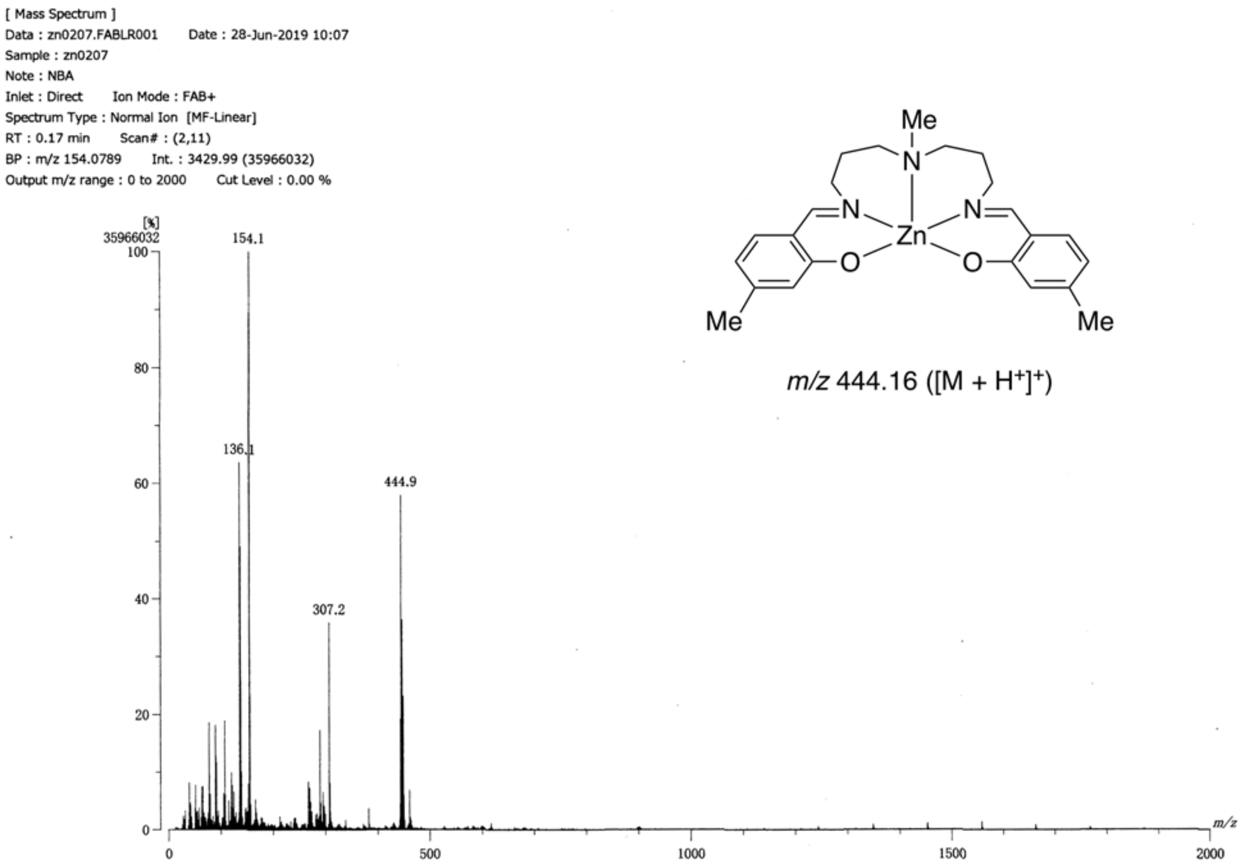

Figure S9. Mass spectrum of Zn($L^{4\text{-}Me}$).

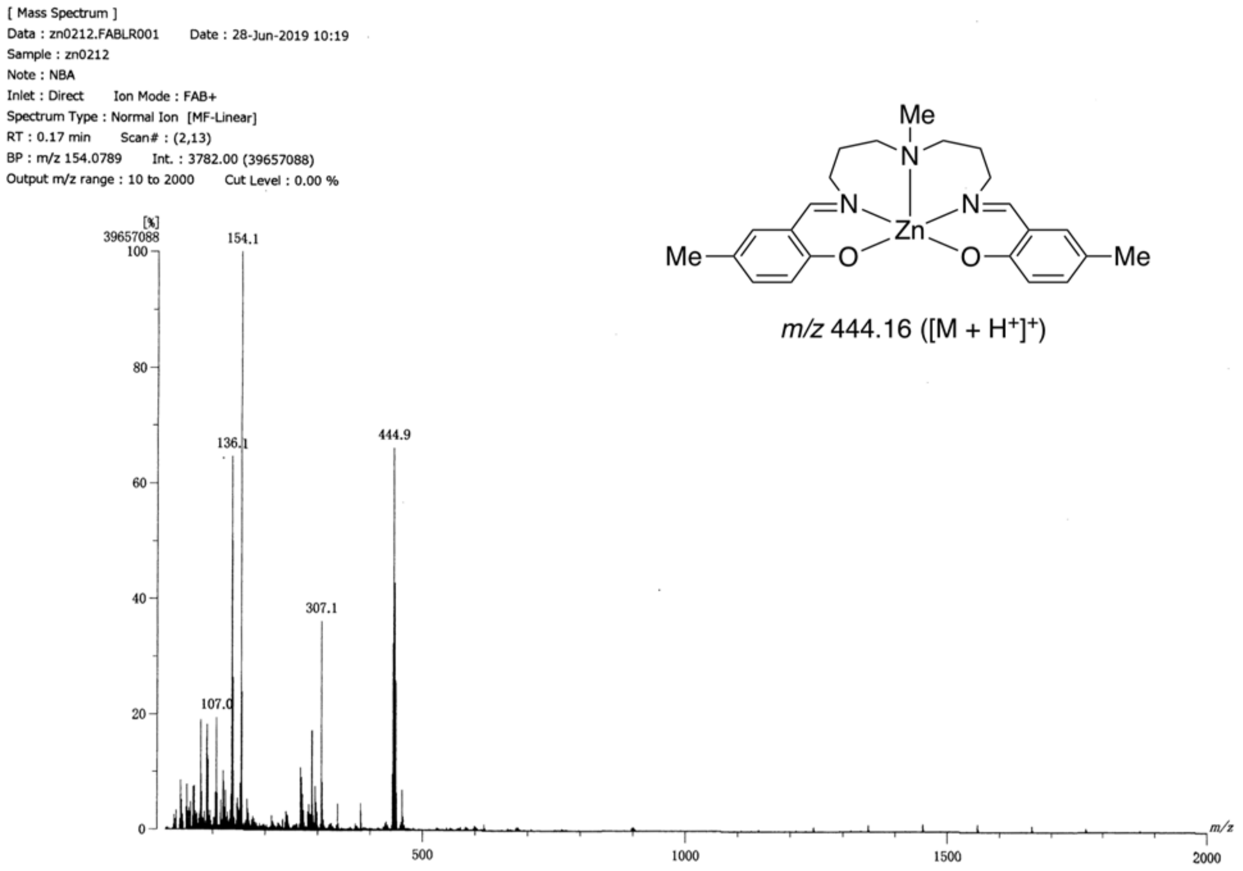

Figure S10. Mass spectrum of Zn($L^{5\text{-}Me}$).

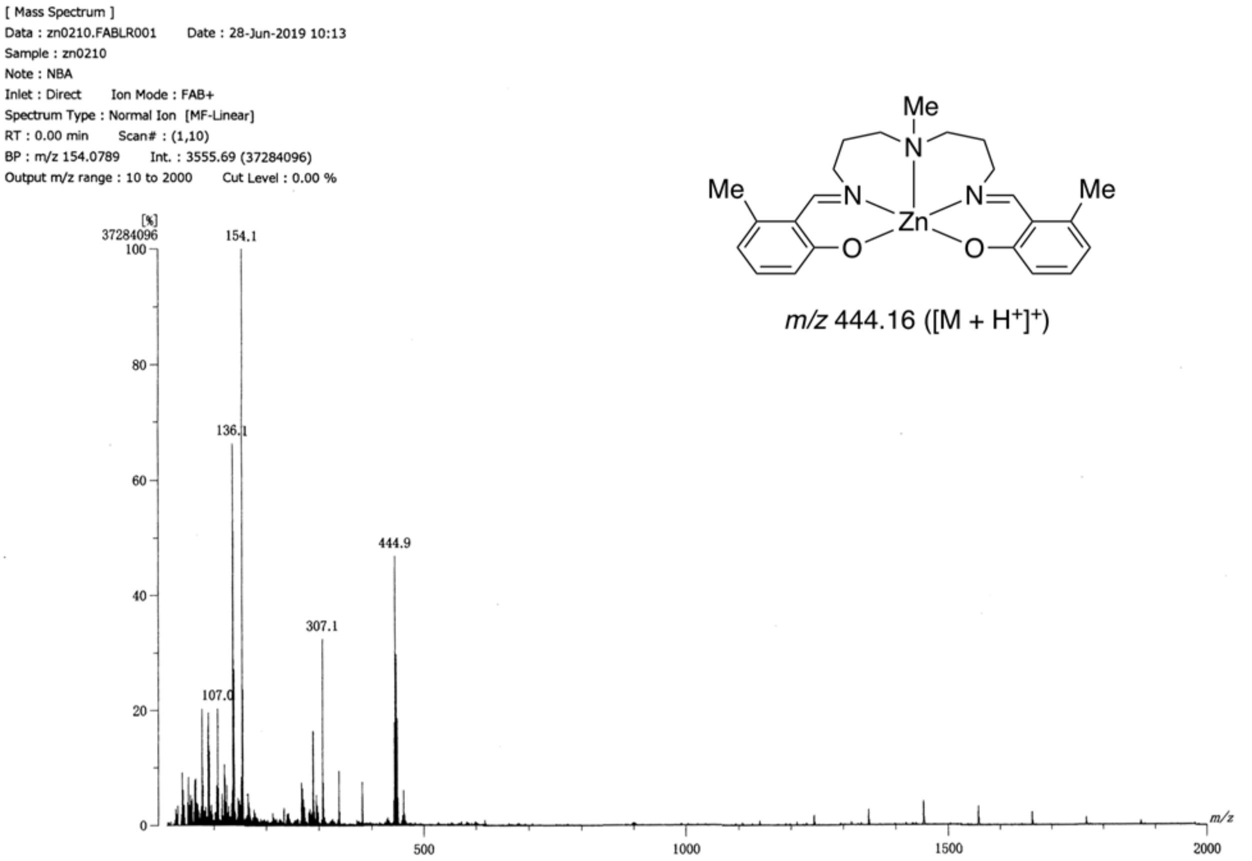

Figure S11. Mass spectrum of Zn($L^{6\text{-}Me}$).

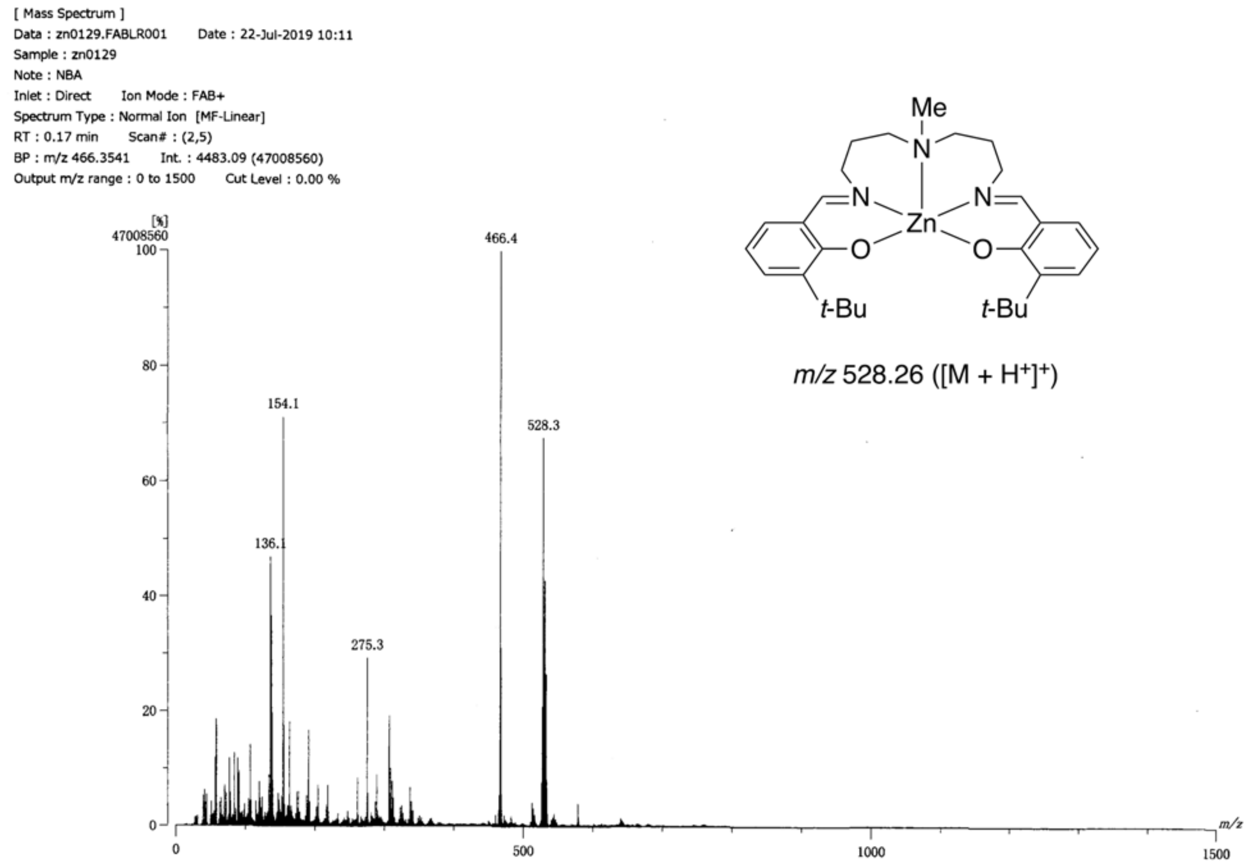

Figure S12. Mass spectrum of Zn(L$^{3\text{-}t\text{-Bu}}$).

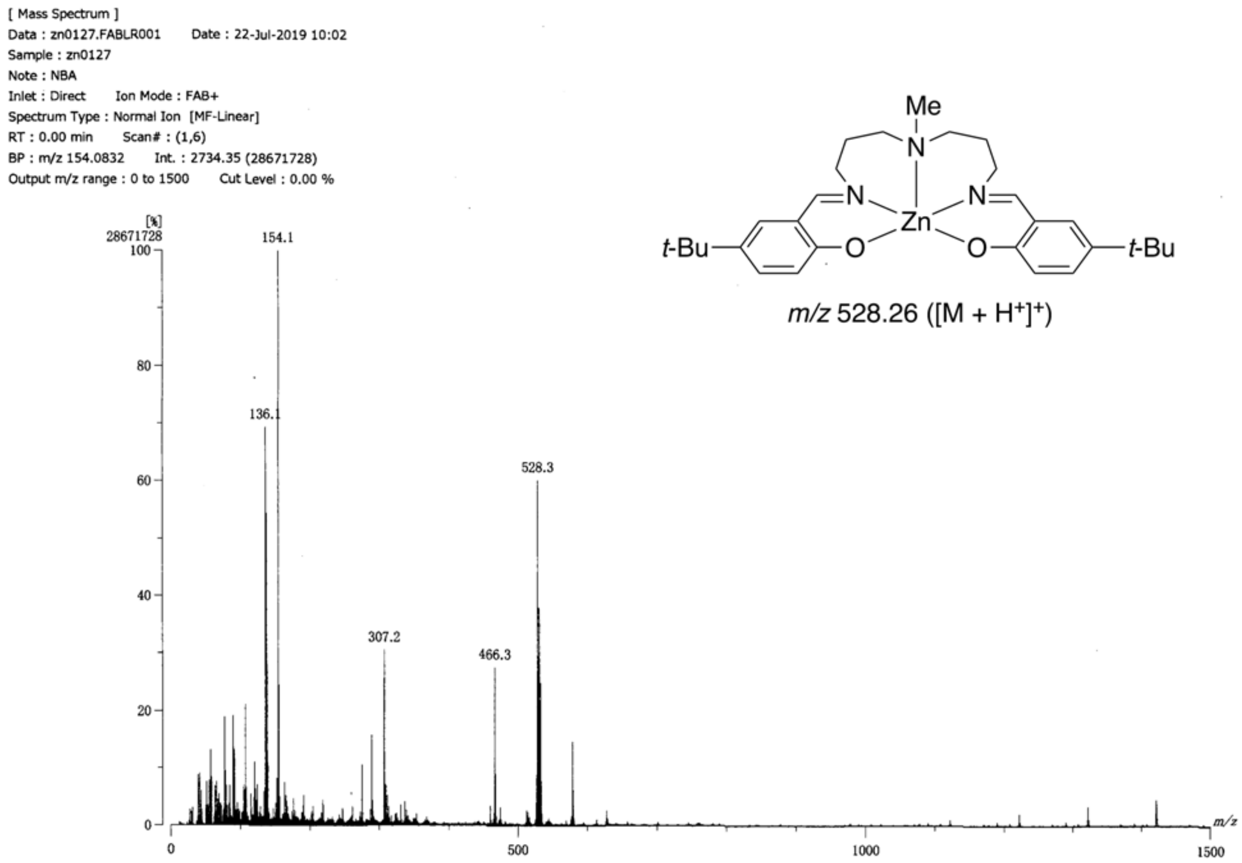

Figure S13. Mass spectrum of Zn($L^{5\text{-}t\text{-}Bu}$).

Table S1. Photophysical Data of Zn($L^{3,5\text{-}t\text{-}Bu}$) in Different Solvent.

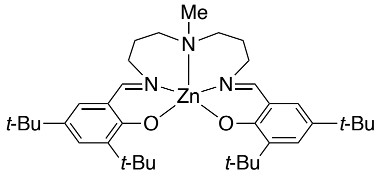

Zn($L^{3,5\text{-}t\text{-}Bu}$)

	λ_{max} (nm) [a]	ε (M^{-1} cm^{-1}) [a]	λ_{em} (nm) [b]	ϕ (%) [c]	τ (ns) [d]
pyridine	373	1.39×10^4	472	21	5.06
toluene	375	1.41×10^4	472	24	4.81
acetone	371	1.26×10^4	472	20	4.29
acetonitrile	370	1.22×10^4	472	14	2.86
methanol	371	1.16×10^4	472	5.2	0.80

[a] Absorption spectra were measured for the 0.5×10^{-3} M solution at 298 K. [b] Fluorescence spectra were measured in deoxygenated solvent (8.5×10^{-7} M) with λ_{ex} = 390 nm at 298 K. [c] Fluorescence quantum yields using 9,10-diphenylanthracene as a standard (ϕ = 100 %). Standard deviations of three independent experiments were less than 5%. [d] Fluorescence life times measured in deoxygenated solvent (1.0×10^{-5} M) with λ_{ex} = 375 nm. Standard deviations of three independent experiments were less than 5%.

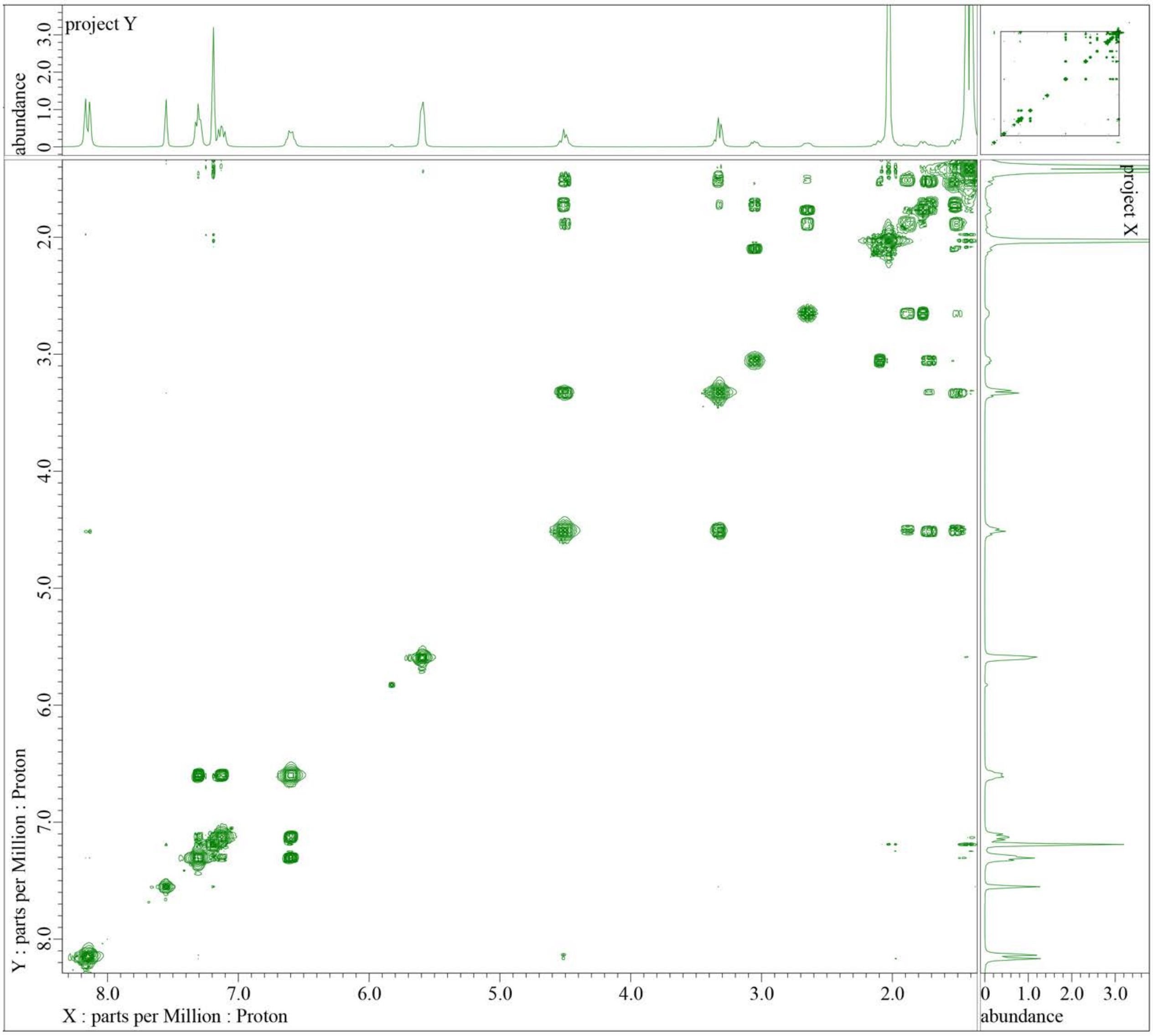

Figure S14. COSY spectrum of Zn(L$^{3\text{-}t\text{-Bu}}$) in pyridine at 253 K (30 mM).

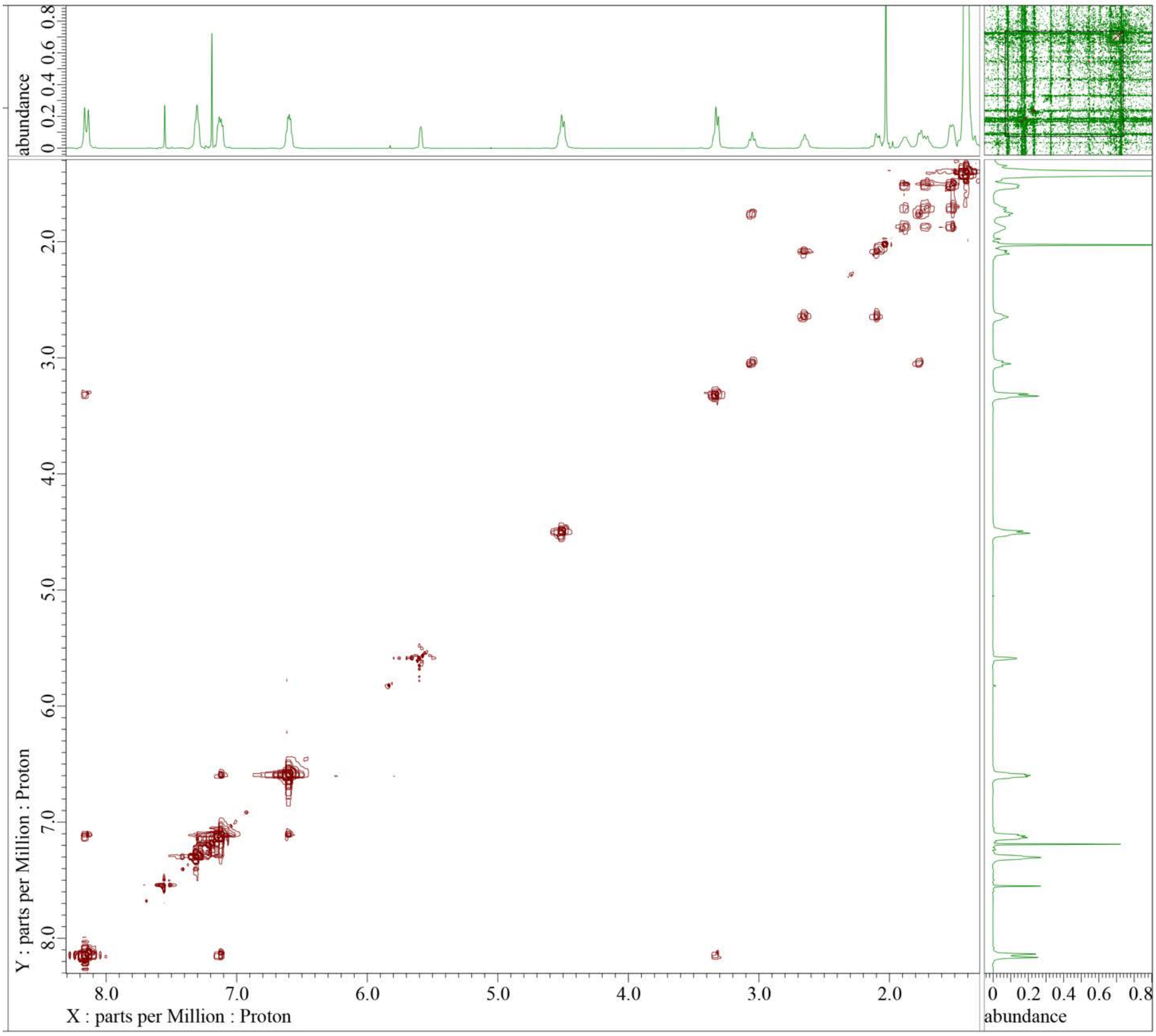

Figure S15. NOESY spectrum of Zn(L$^{3\text{-}t\text{-Bu}}$) in pyridine at 253 K (30 mM).

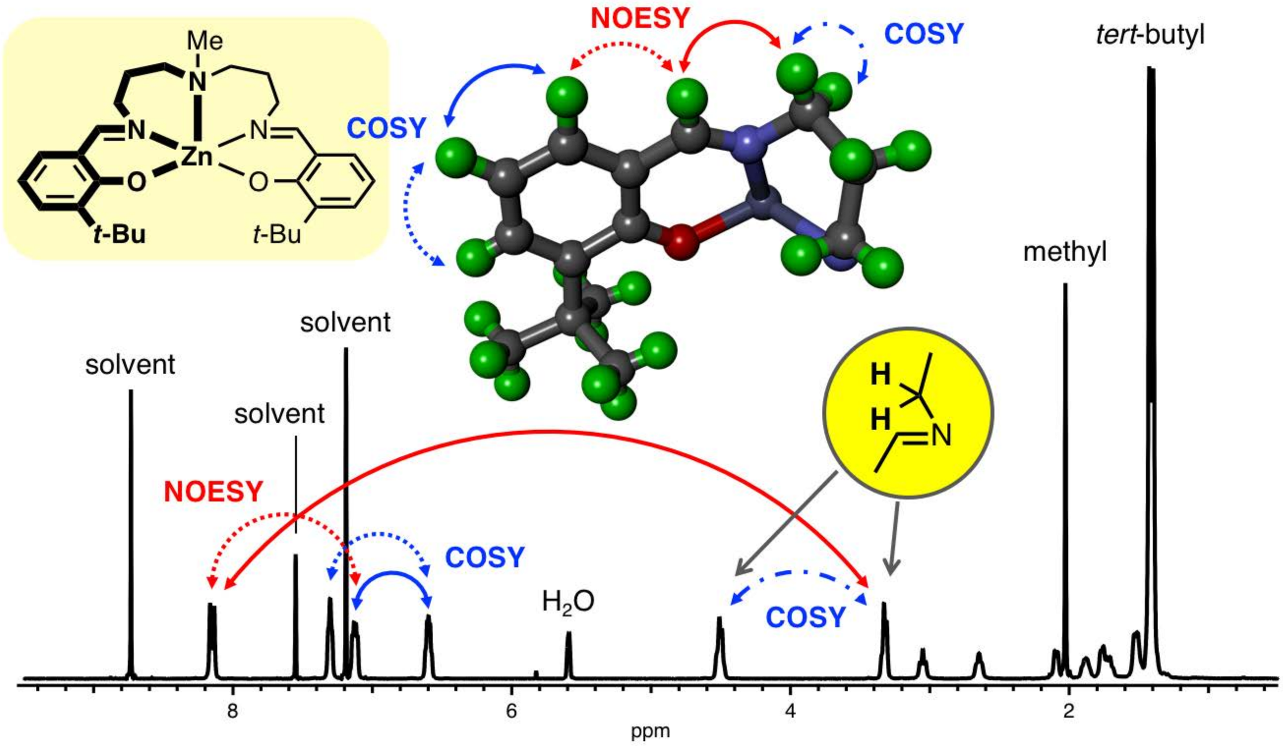

Figure S16. Summary of 2D NMR experiments shown in Figure S14 and S15.

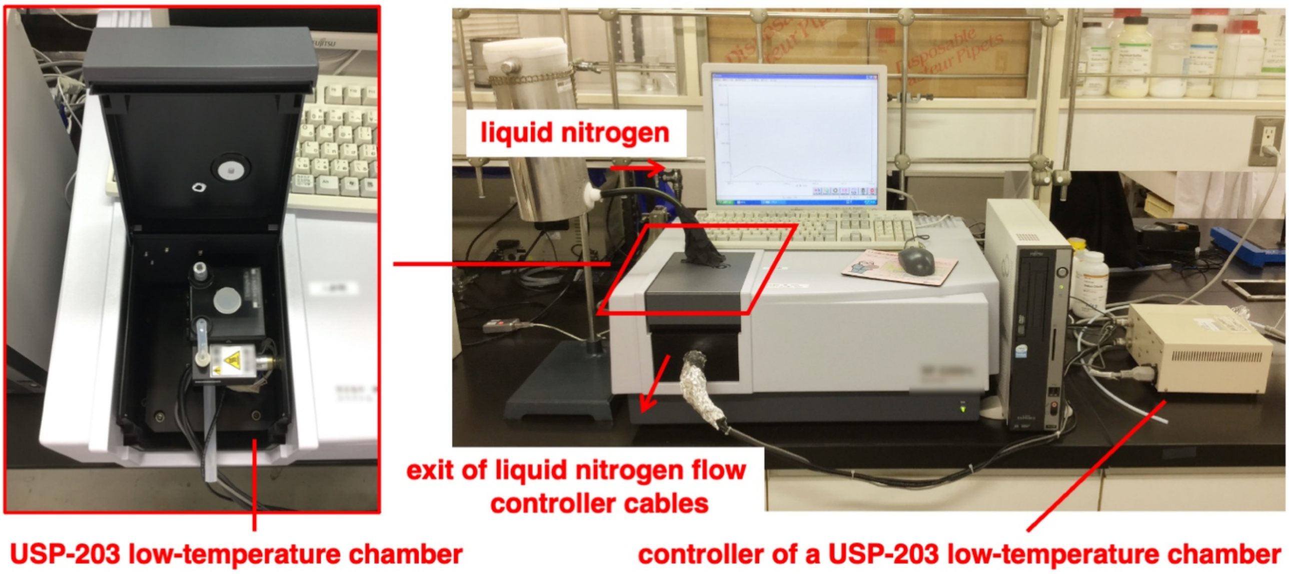

Figure S17. Experimental setups for low-temperature fluorescence measurements.

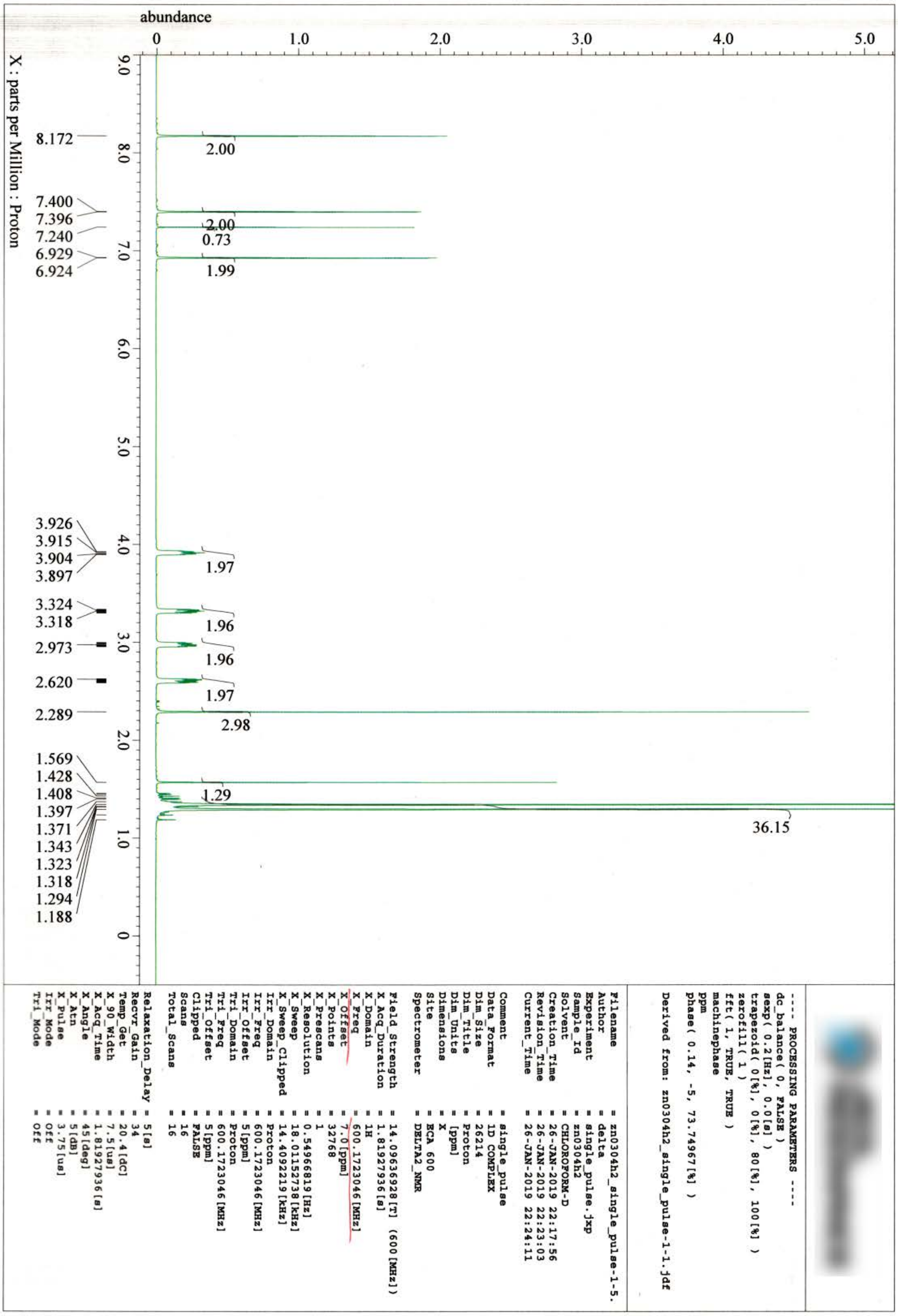

Figure S18. ^{1}H NMR spectrum of Zn(L’ $^{3,5\text{-t-Bu}}$) in $CDCl_3$.

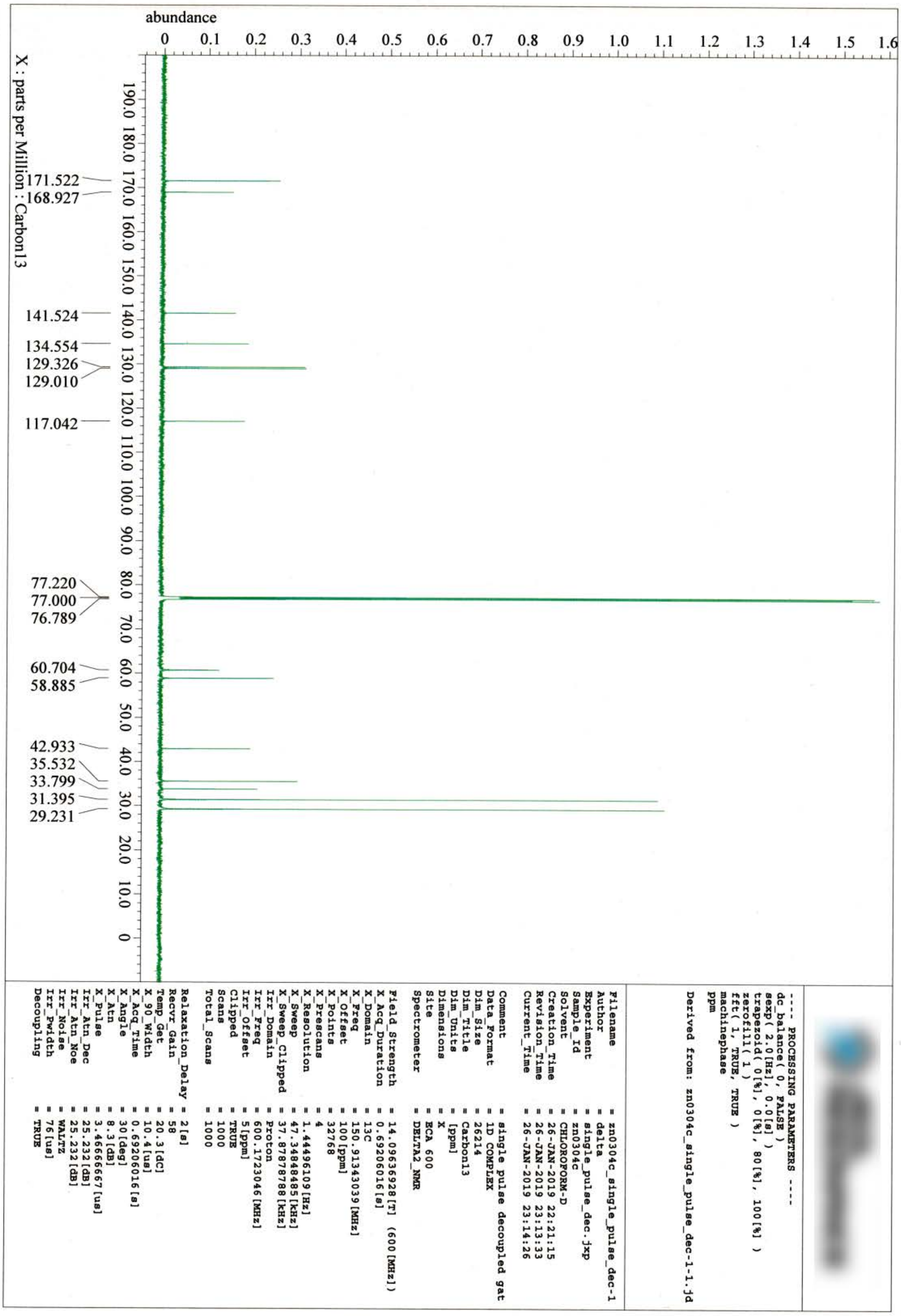

Figure S19. ^{13}C NMR spectrum of Zn(L’ $^{3,5\text{-}t\text{-Bu}}$) in $CDCl_3$.

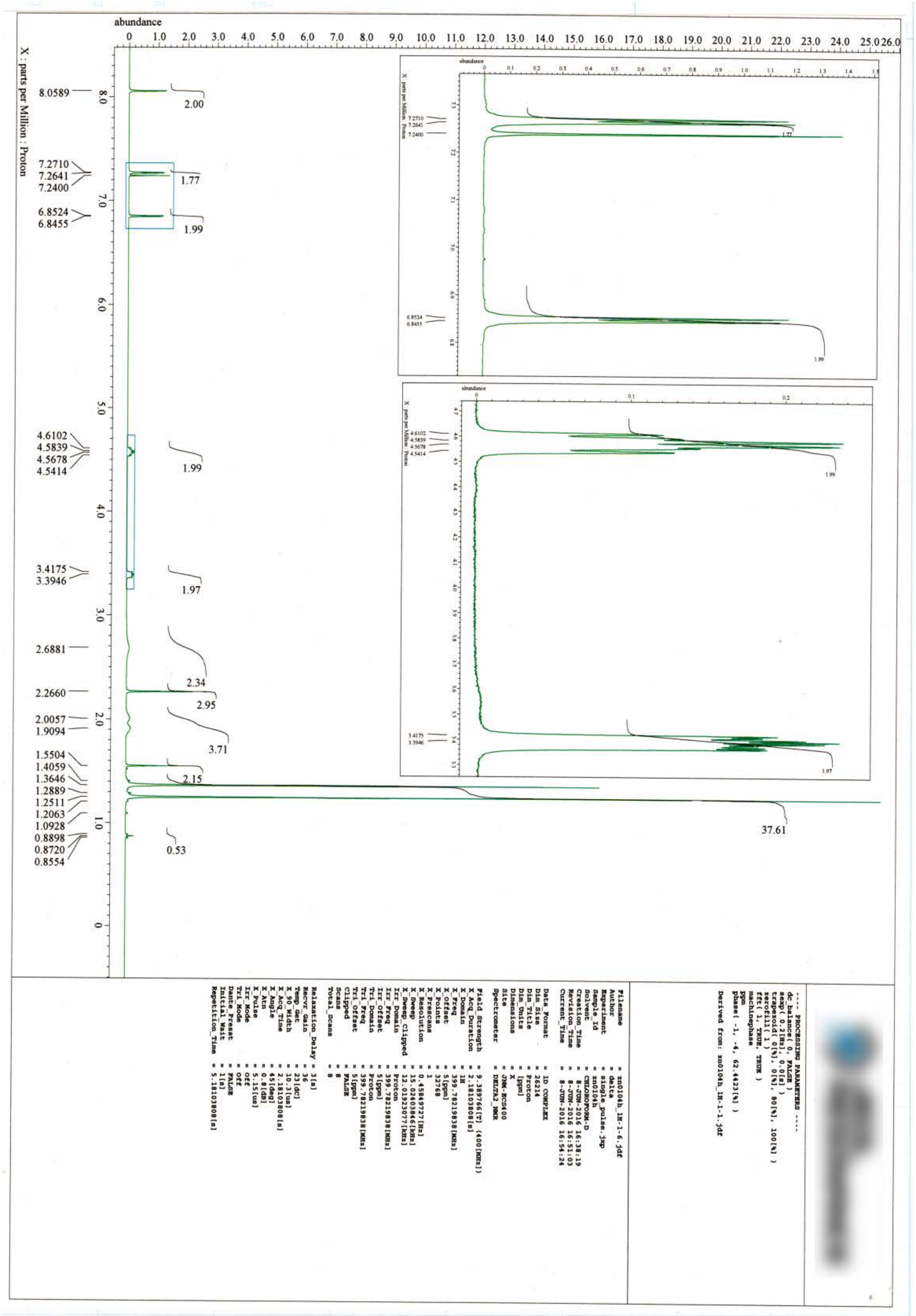

Figure S20. ^{1}H NMR spectrum of Zn($L^{3,5\text{-}t\text{-Bu}}$) in $CDCl_3$.

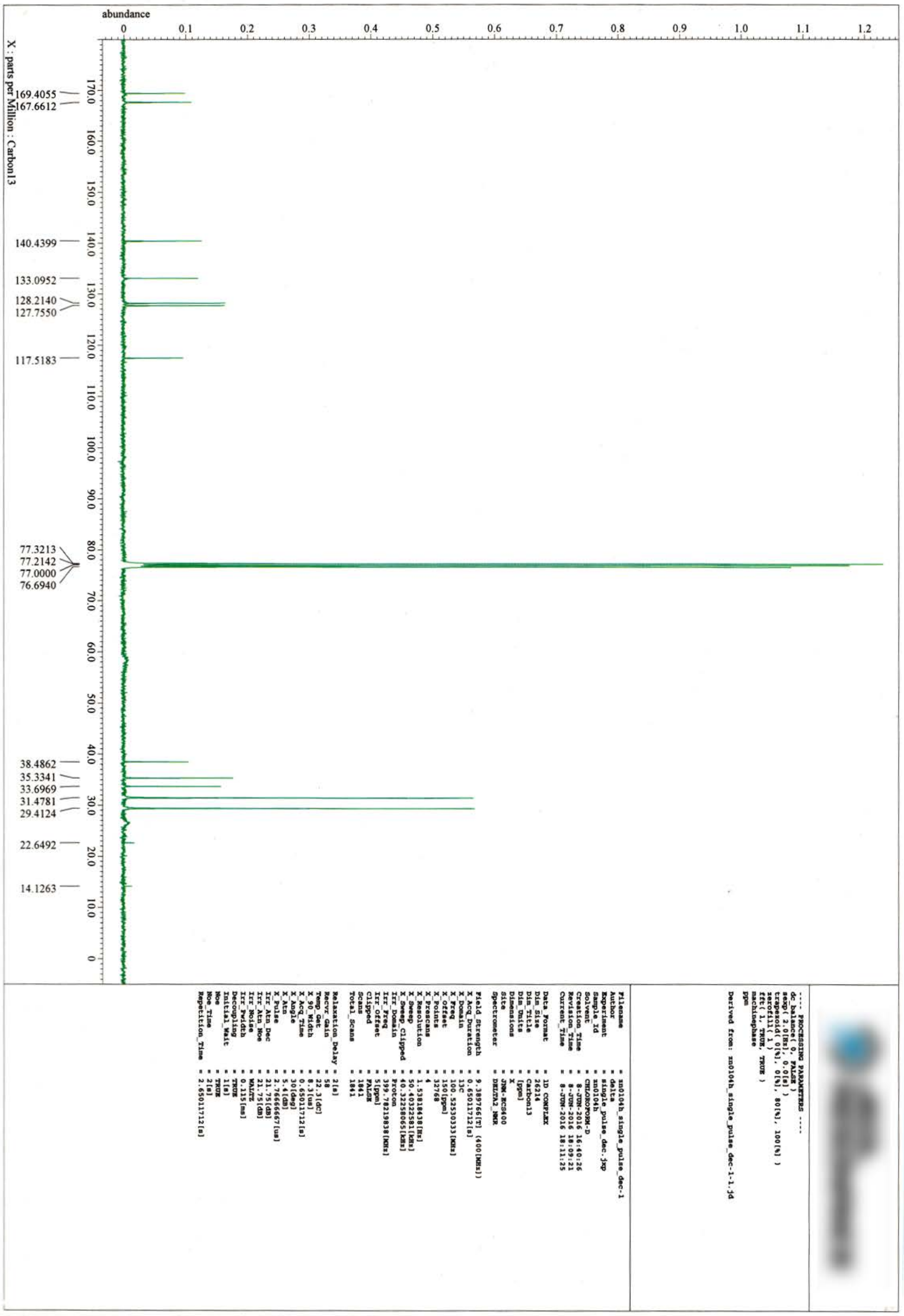

Figure S21. ^{13}C NMR spectrum of Zn(L$^{3,5\text{-}t\text{-Bu}}$) in $CDCl_3$.

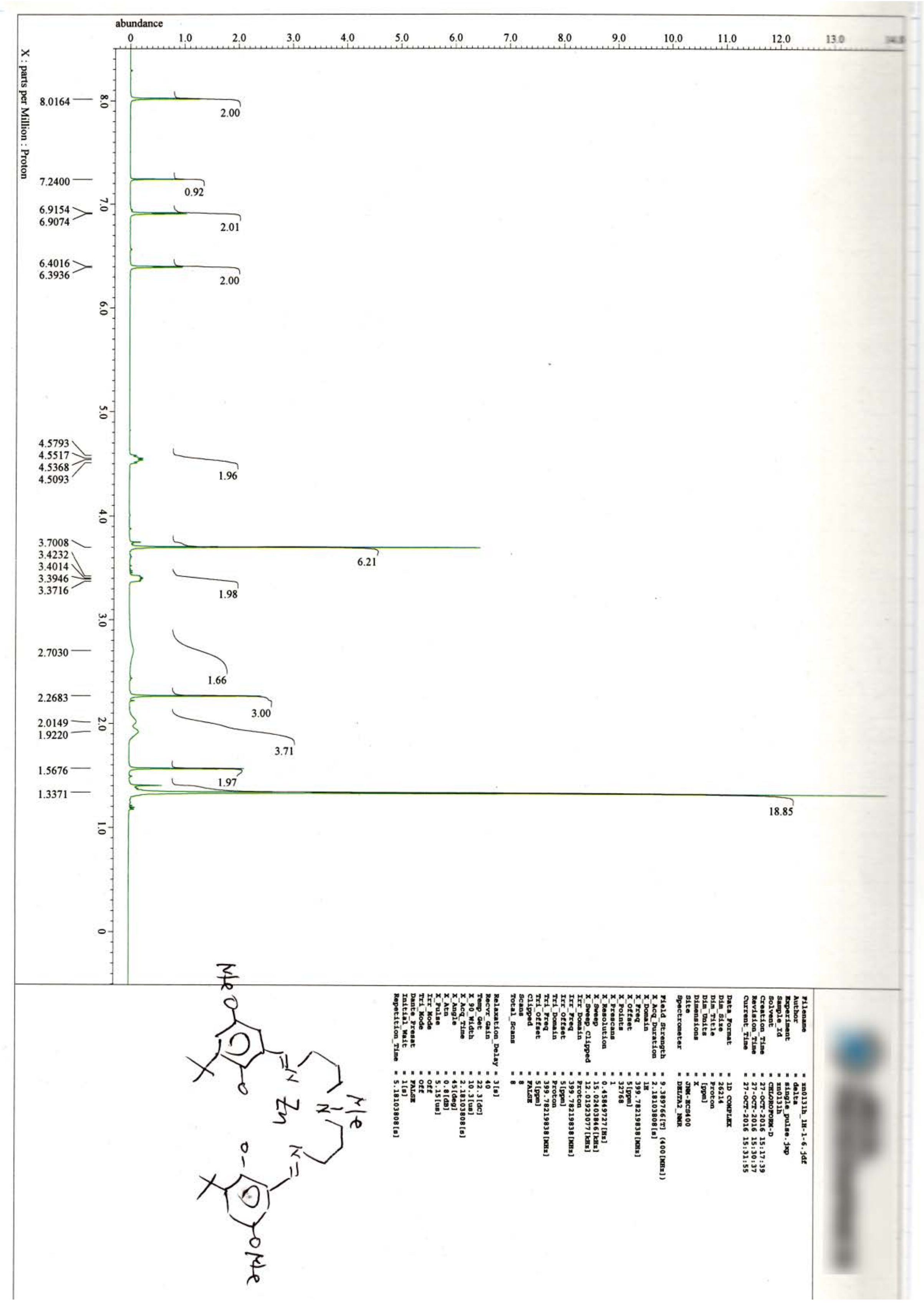

Figure S22. ^{1}H NMR spectrum of Zn($L^{3\text{-}t\text{-Bu},5\text{-MeO}}$) in $CDCl_3$.

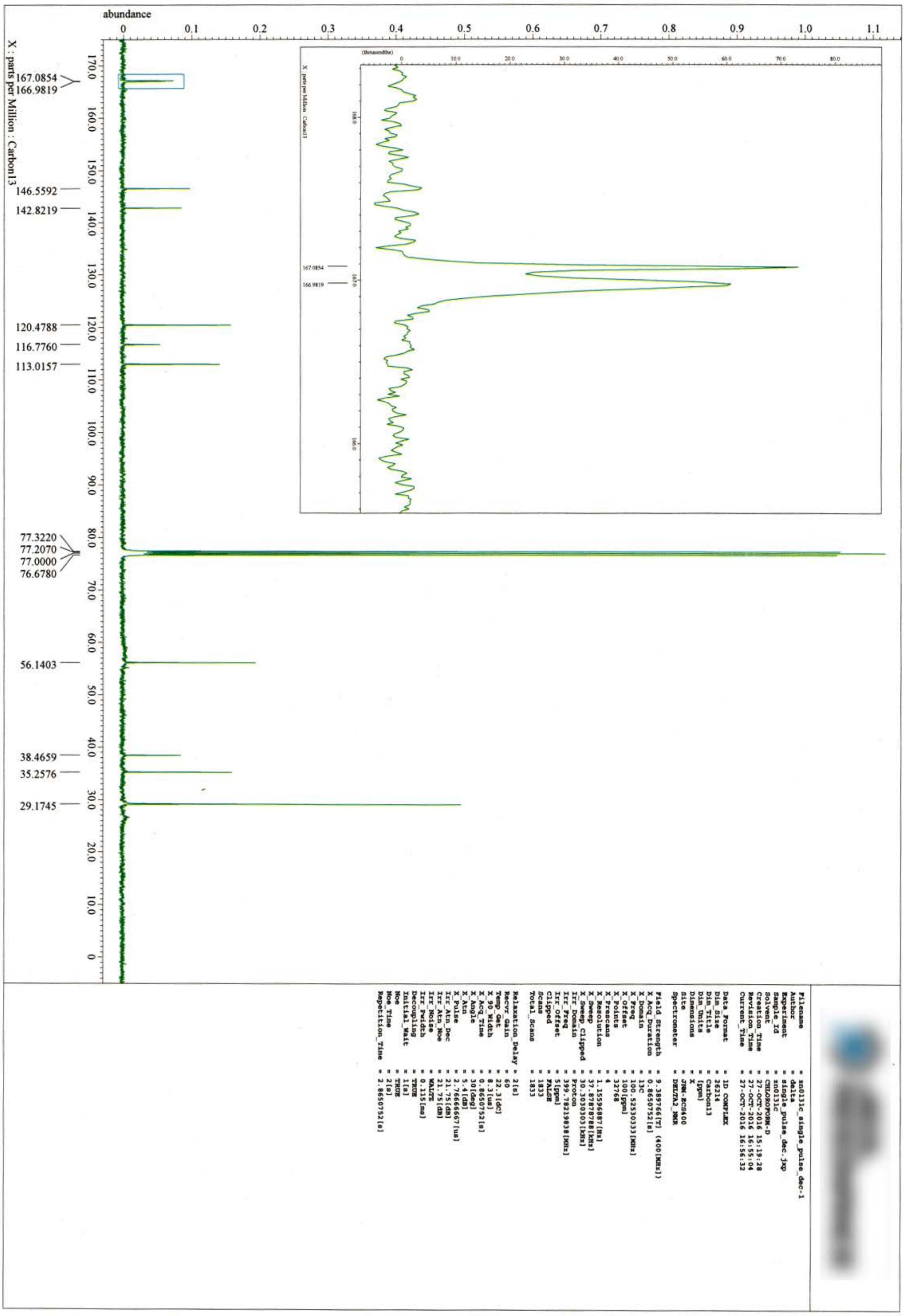

Figure S23. ^{13}C NMR spectrum of Zn($L^{3\text{-}t\text{-Bu},5\text{-MeO}}$) in $CDCl_3$.

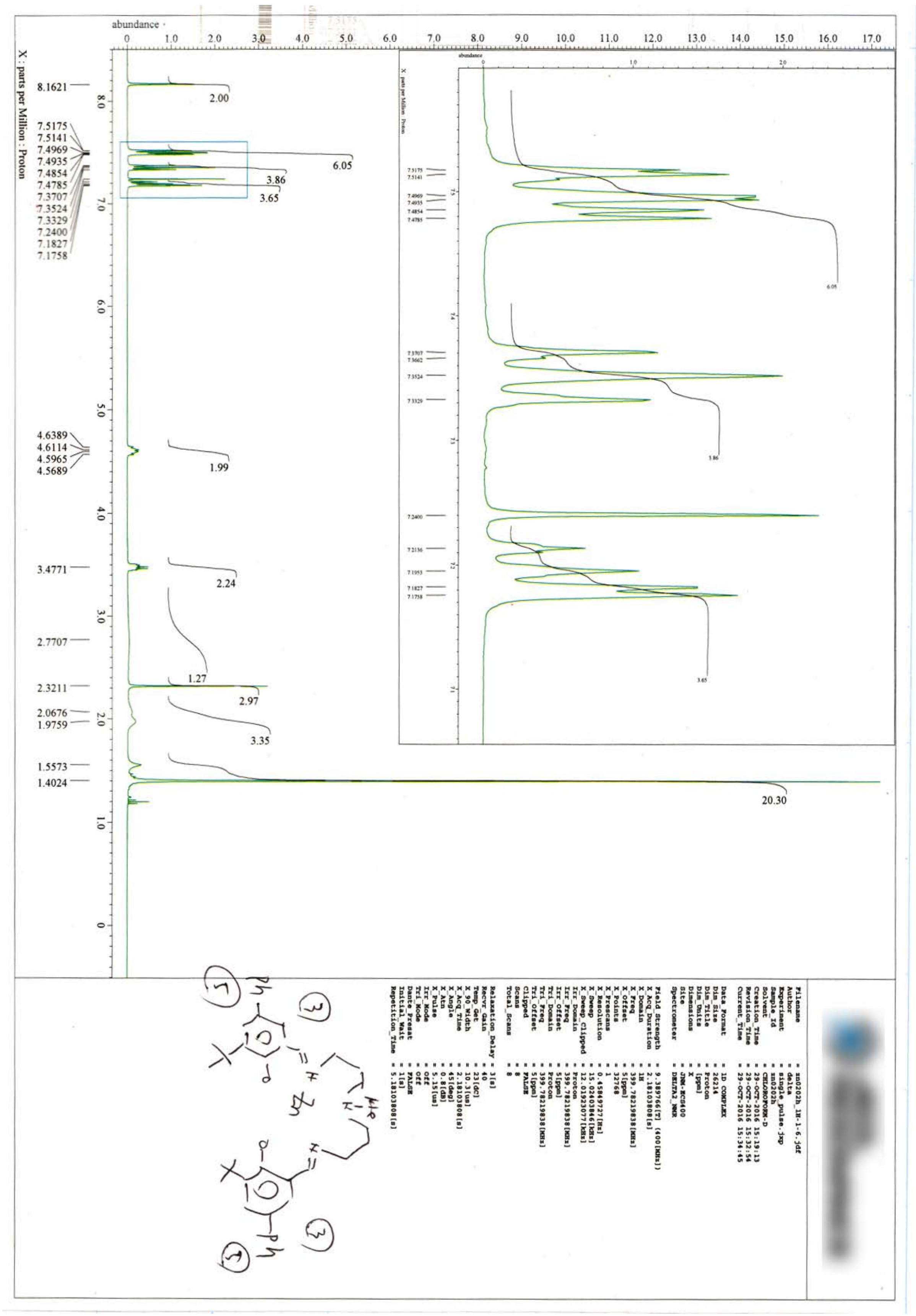

Figure S24. ${}^{1}H$ NMR spectrum of $Zn(L^{3\text{-}t\text{-}Bu,5\text{-}Ph})$ in $CDCl_3$.

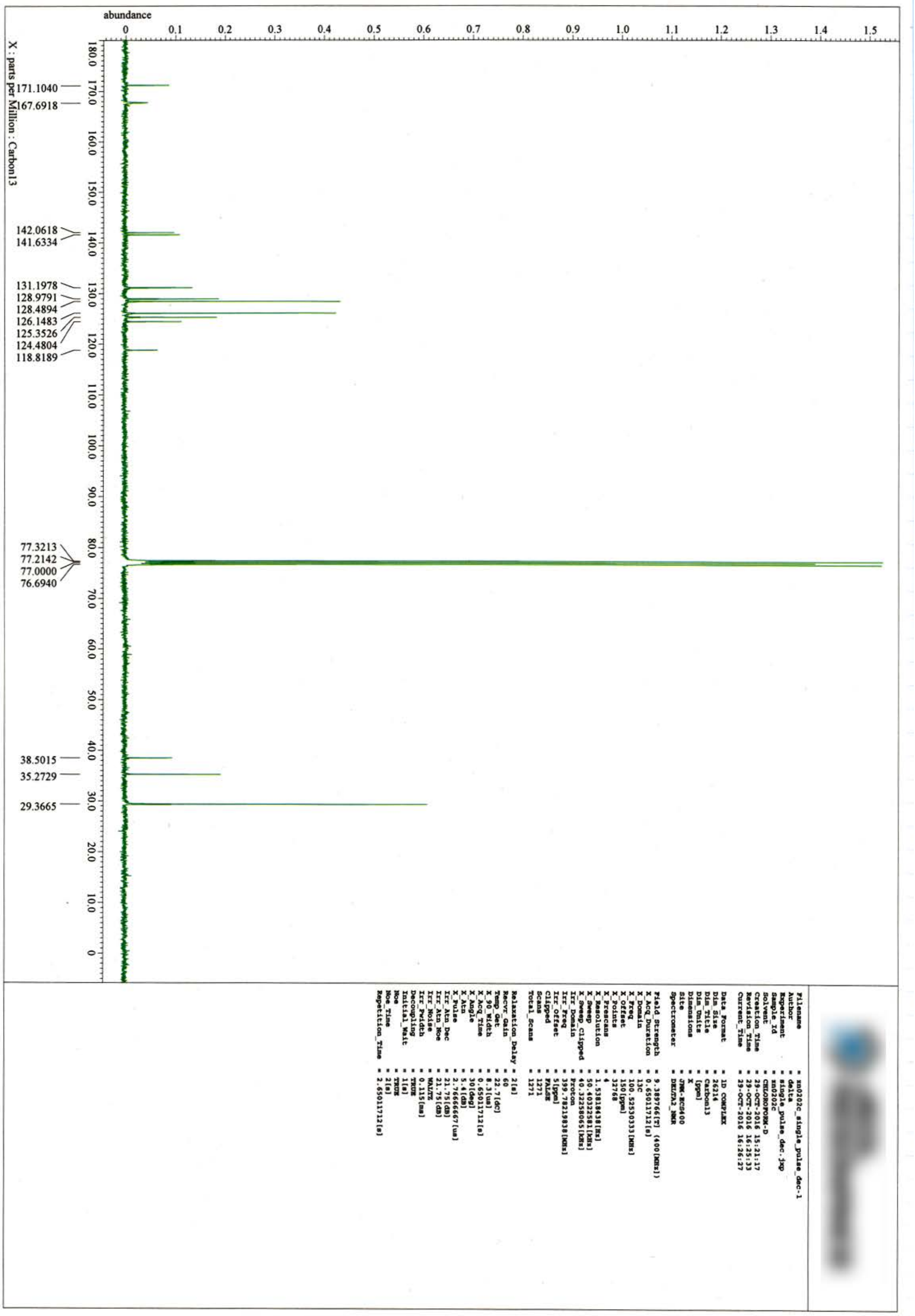

Figure S25. ^{13}C NMR spectrum of Zn($L^{3\text{-}t\text{-Bu},5\text{-Ph}}$) in $CDCl_3$.

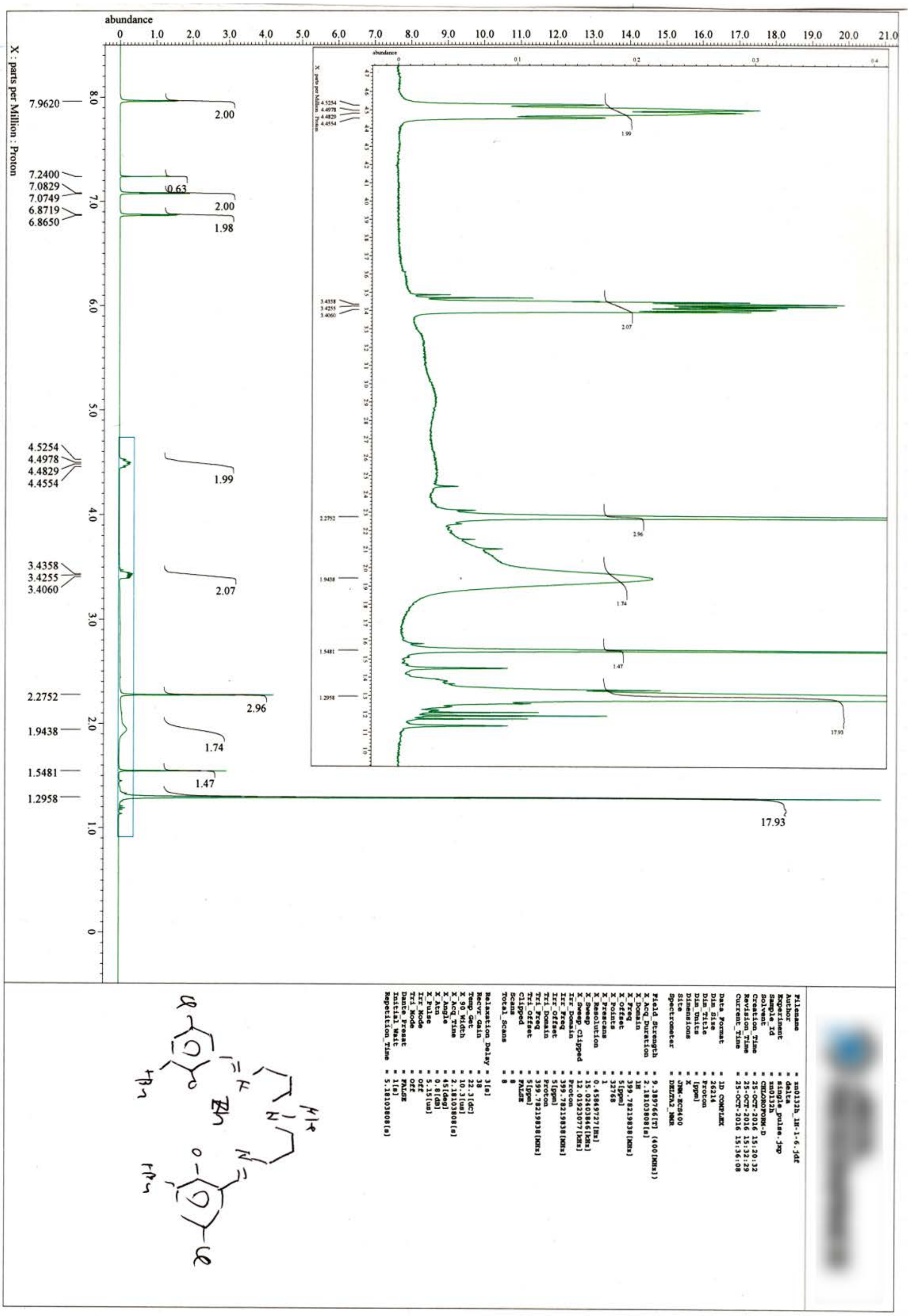

Figure S26. ^{1}H NMR spectrum of Zn($L^{3\text{-}t\text{-Bu},5\text{-Cl}}$) in $CDCl_3$.

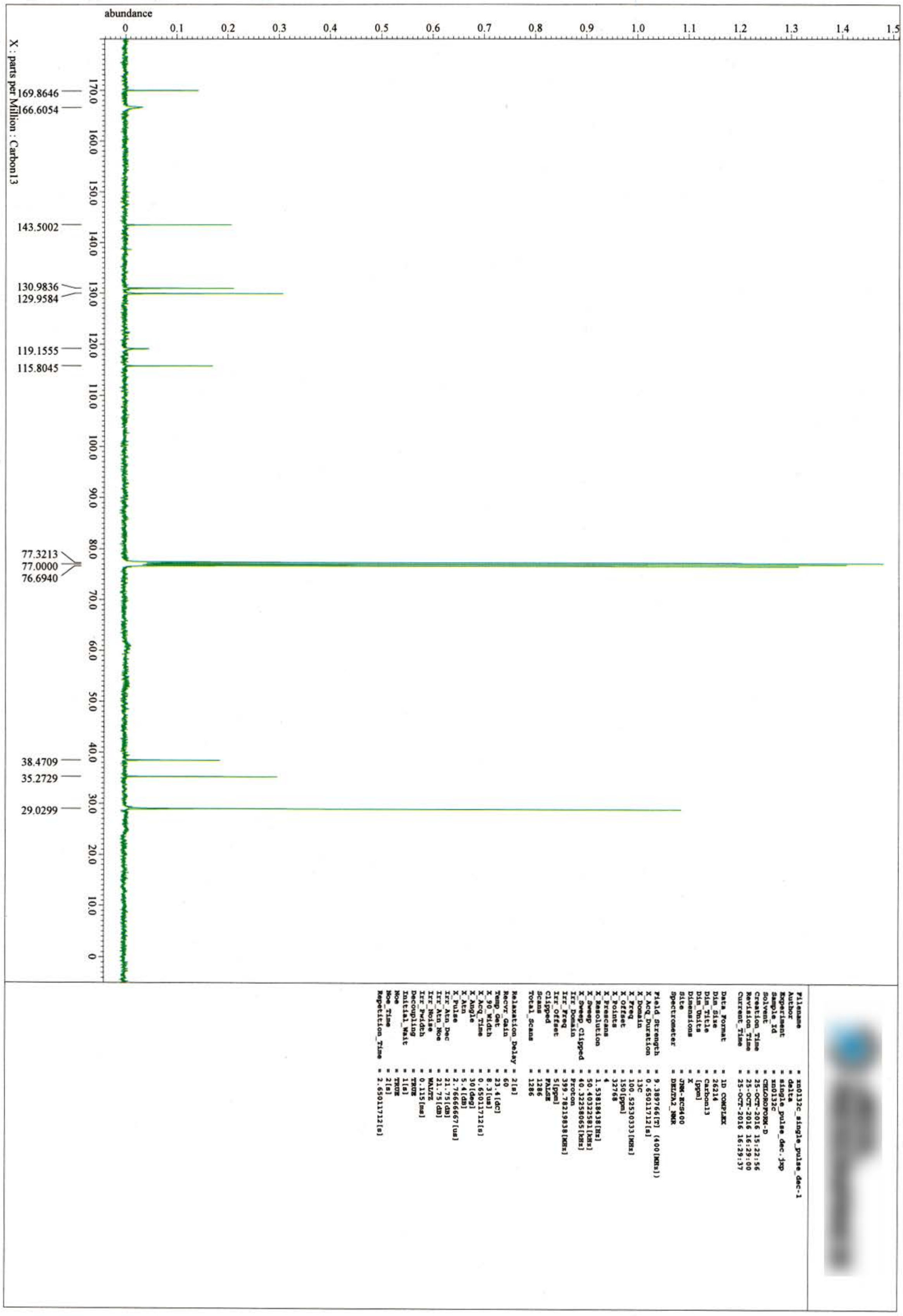

Figure S27. ^{13}C NMR spectrum of Zn($L^{3\text{-}t\text{-Bu},5\text{-Cl}}$) in $CDCl_3$.

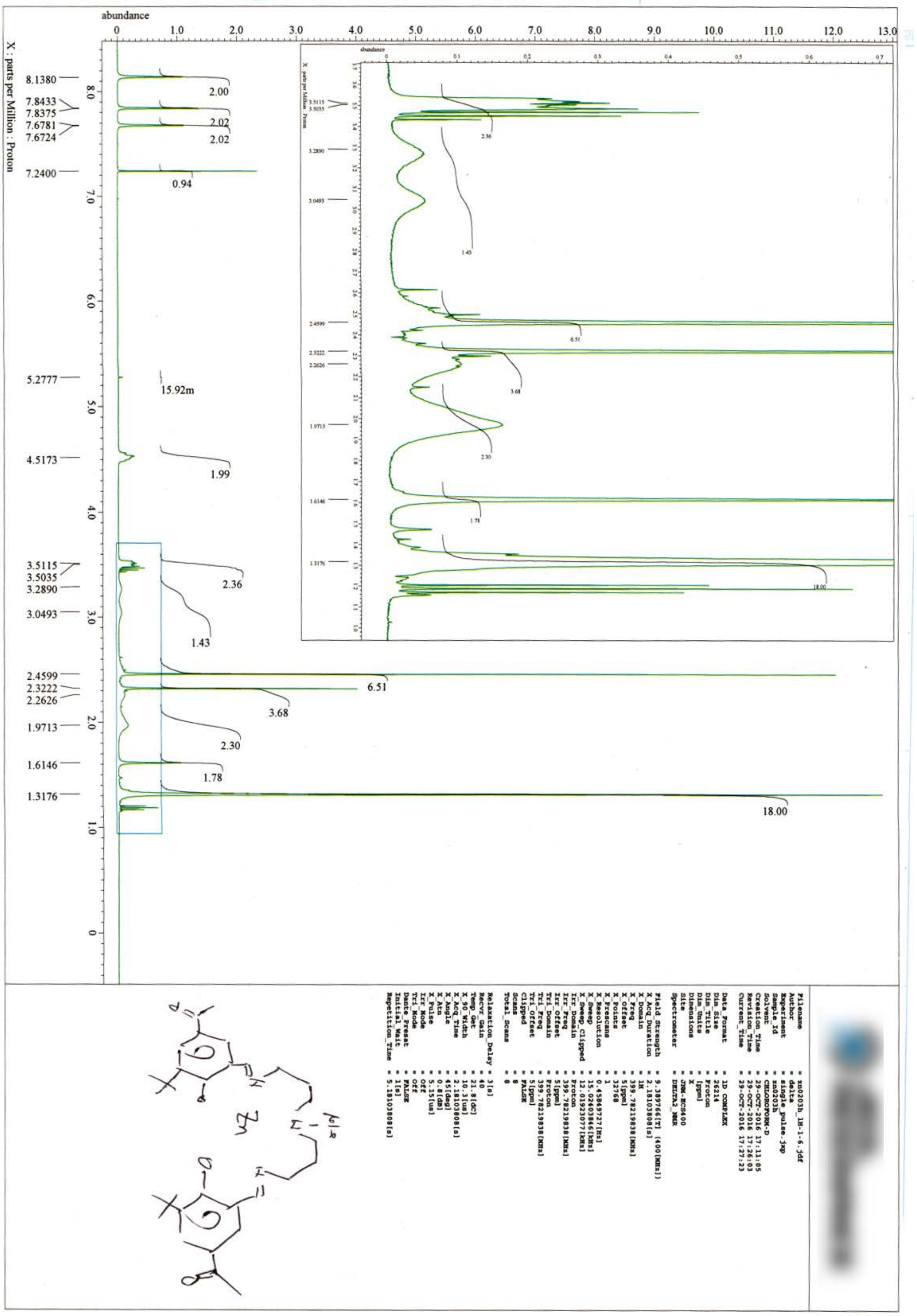

Figure S28. ^{1}H NMR spectrum of $Zn(L^{3\text{-}t\text{-}Bu,5\text{-}MeCO})$ in $CDCl_3$.

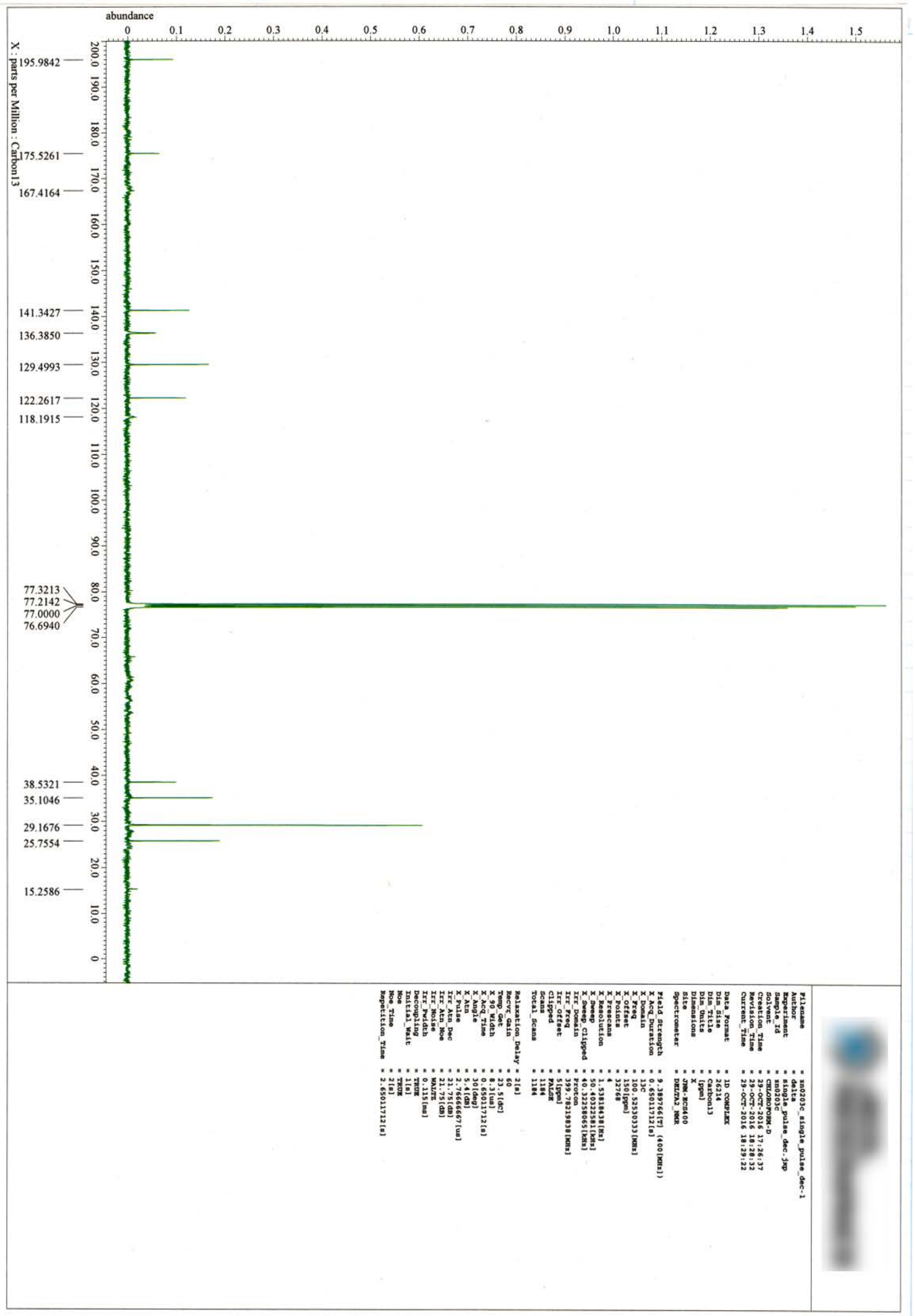

Figure S29. ^{13}C NMR spectrum of Zn($L^{3\text{-}t\text{-Bu},5\text{-MeCO}}$) in $CDCl_3$.

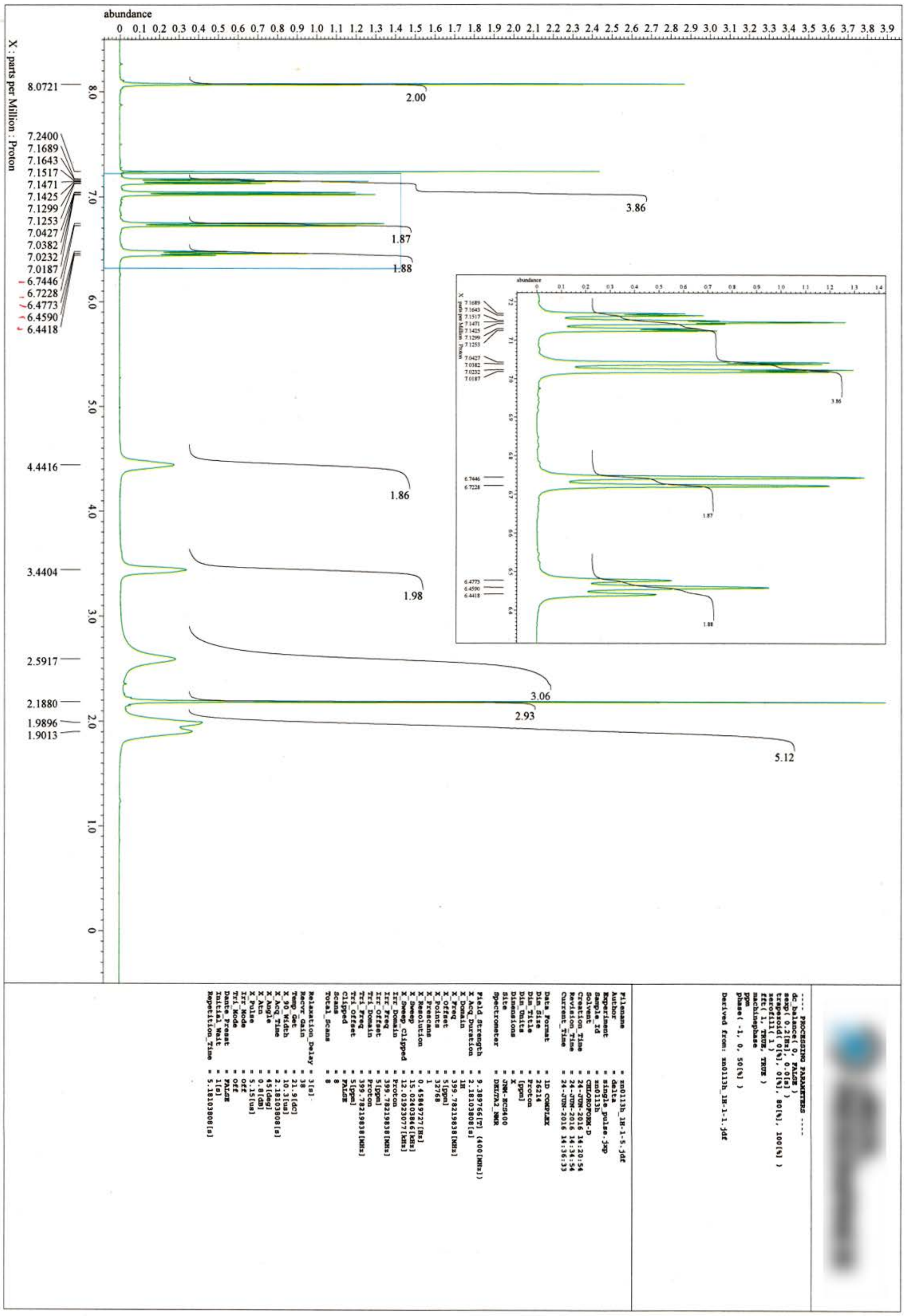

Figure S30. ^{1}H NMR spectrum of Zn(L) in $CDCl_3$.

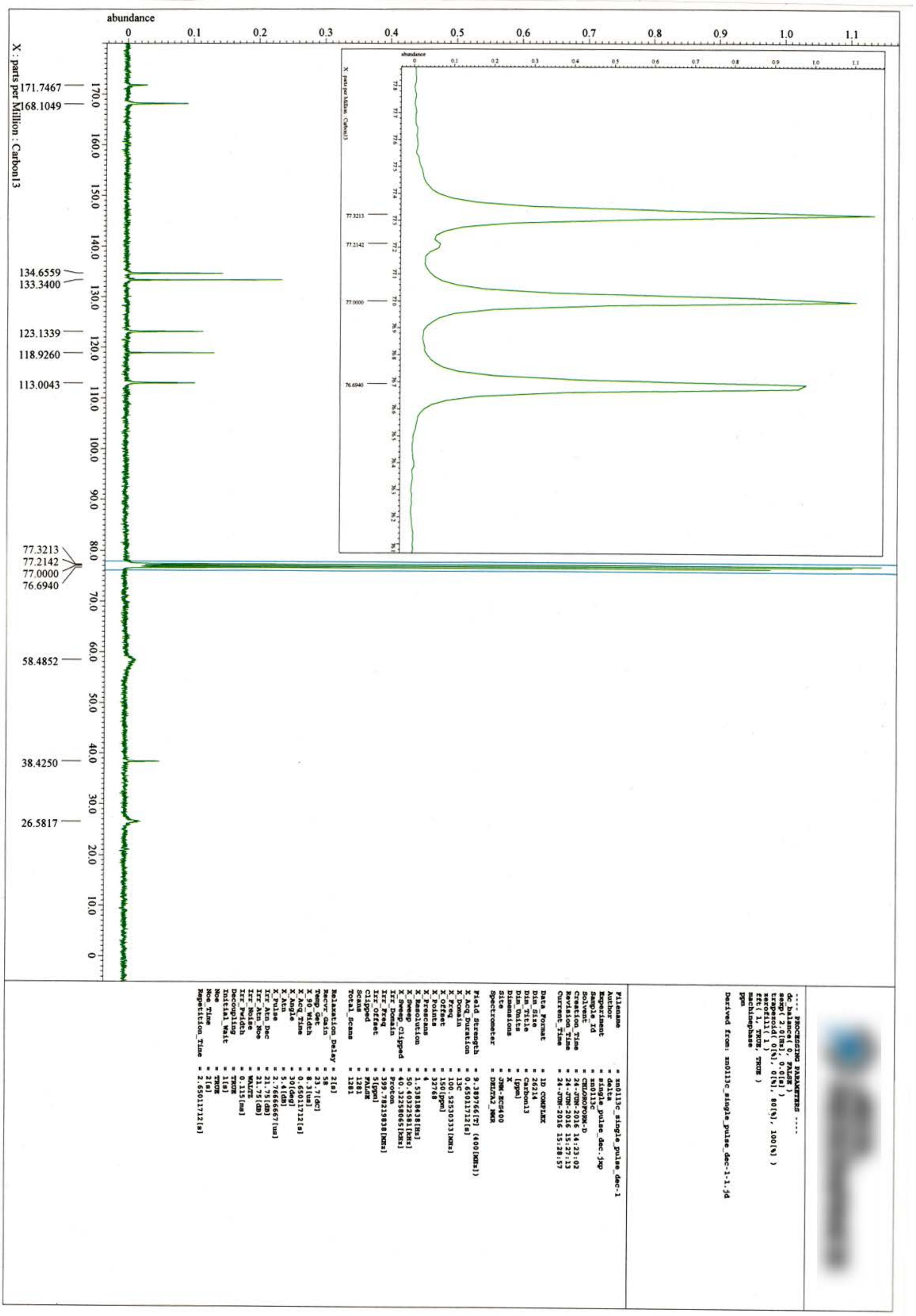

Figure S31. ^{13}C NMR spectrum of Zn(L) in $CDCl_3$.

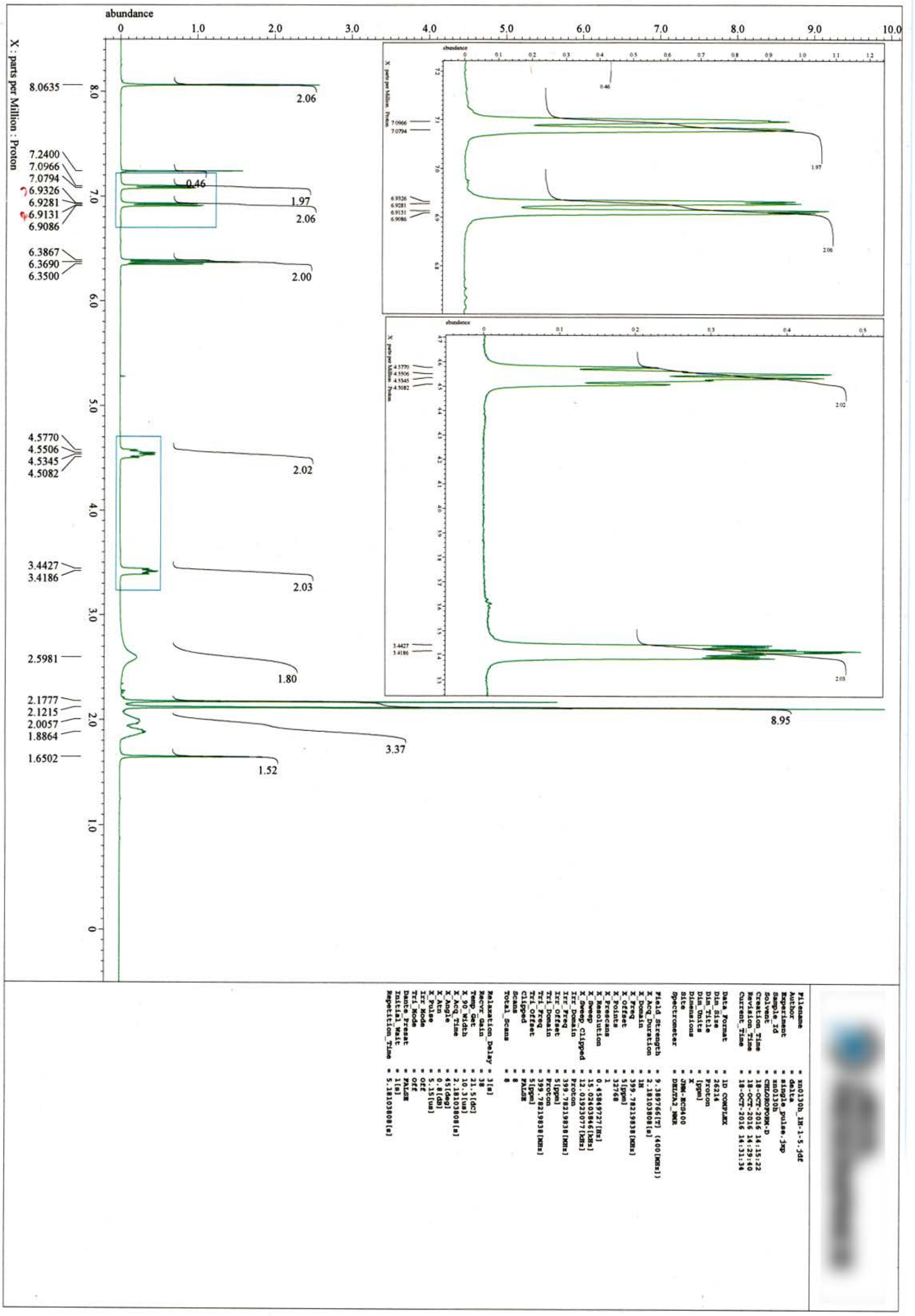

Figure S32. ^{1}H NMR spectrum of Zn($L^{3\text{-}Me}$) in $CDCl_3$.

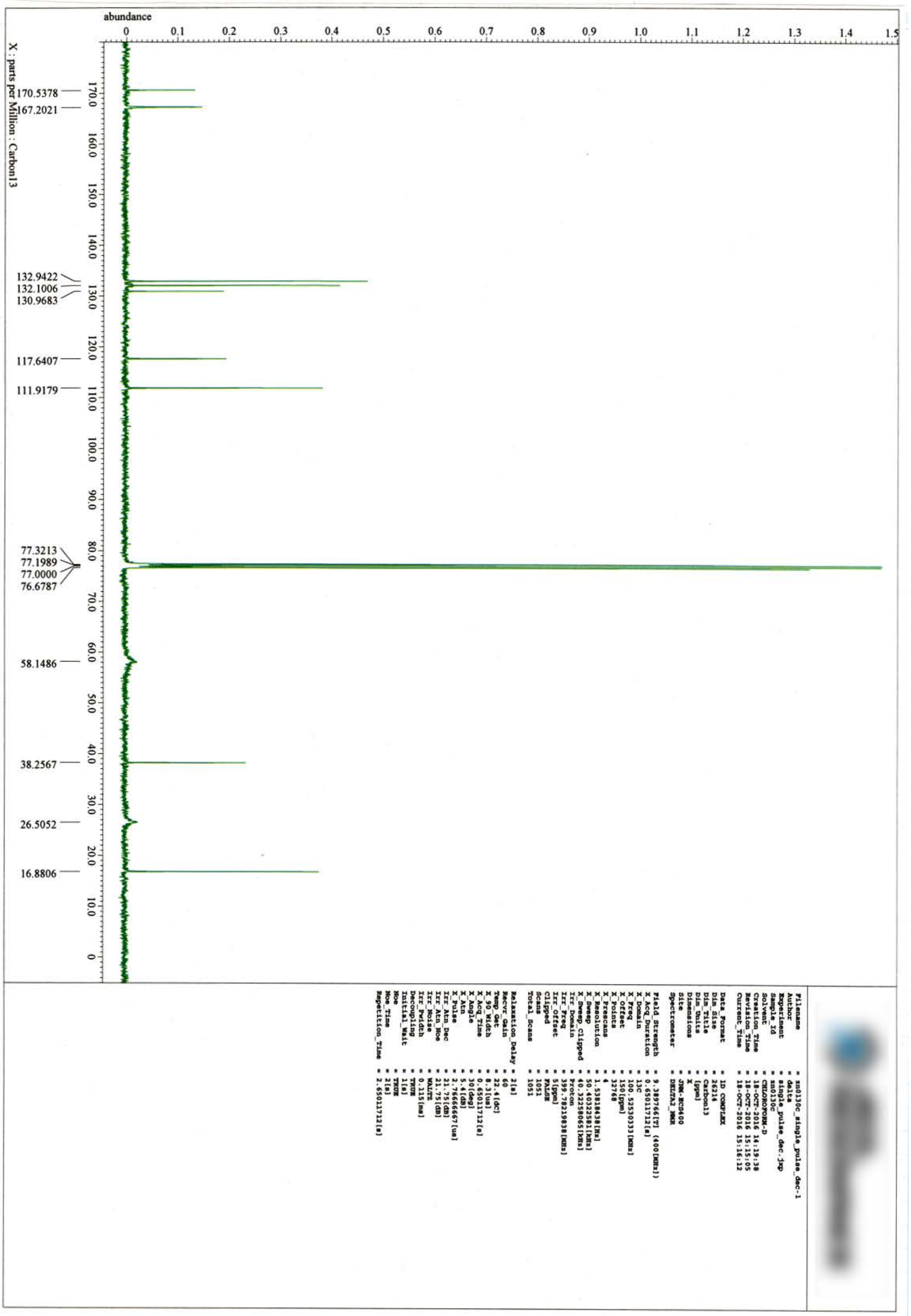

Figure S33. ^{13}C NMR spectrum of Zn($L^{3\text{-}Me}$) in $CDCl_3$.

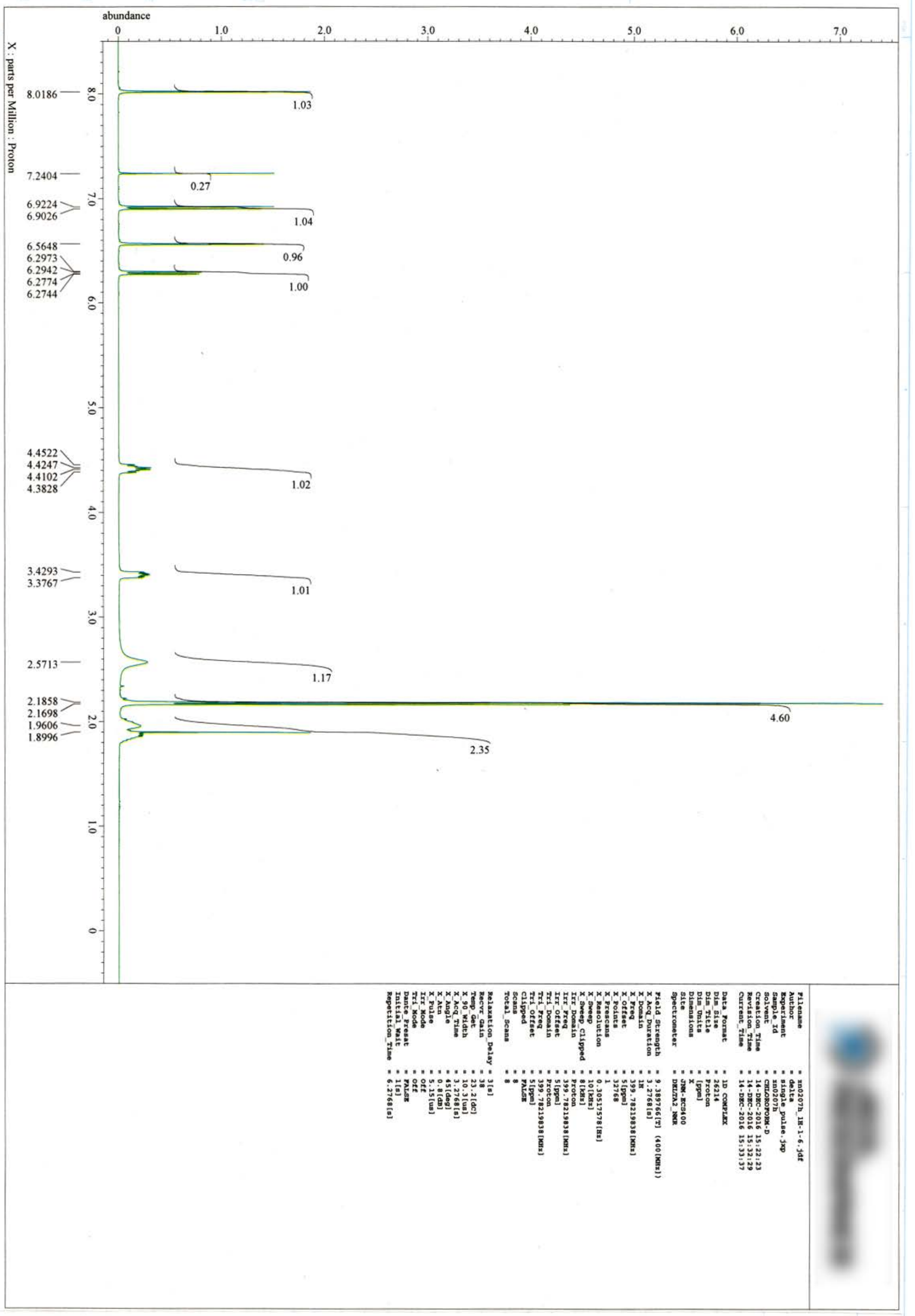

Figure S34. ^{1}H NMR spectrum of Zn($L^{4\text{-}Me}$) in $CDCl_3$.

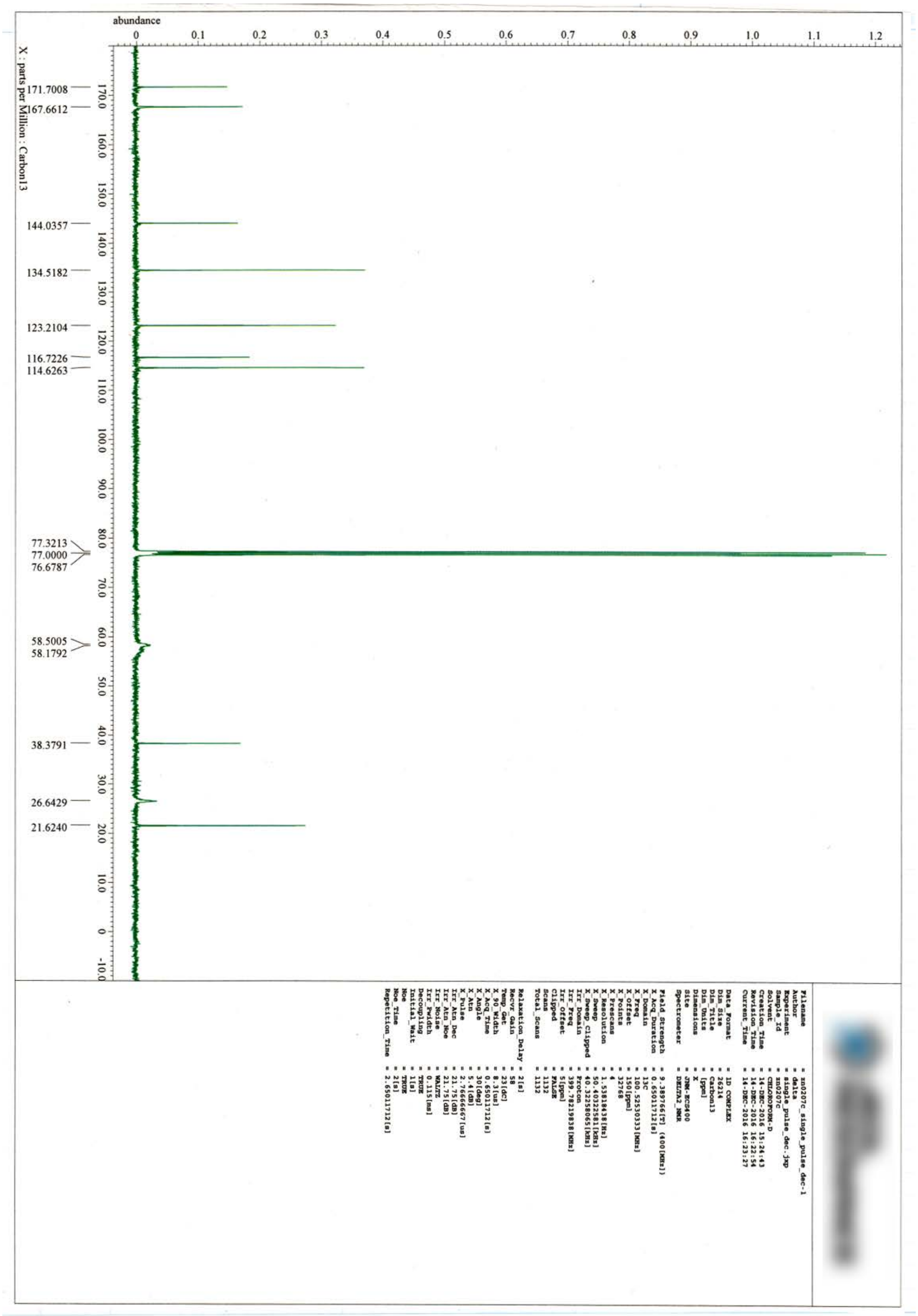

Figure S35. ^{13}C NMR spectrum of $Zn(L^{4\text{-}Me})$ in $CDCl_3$.

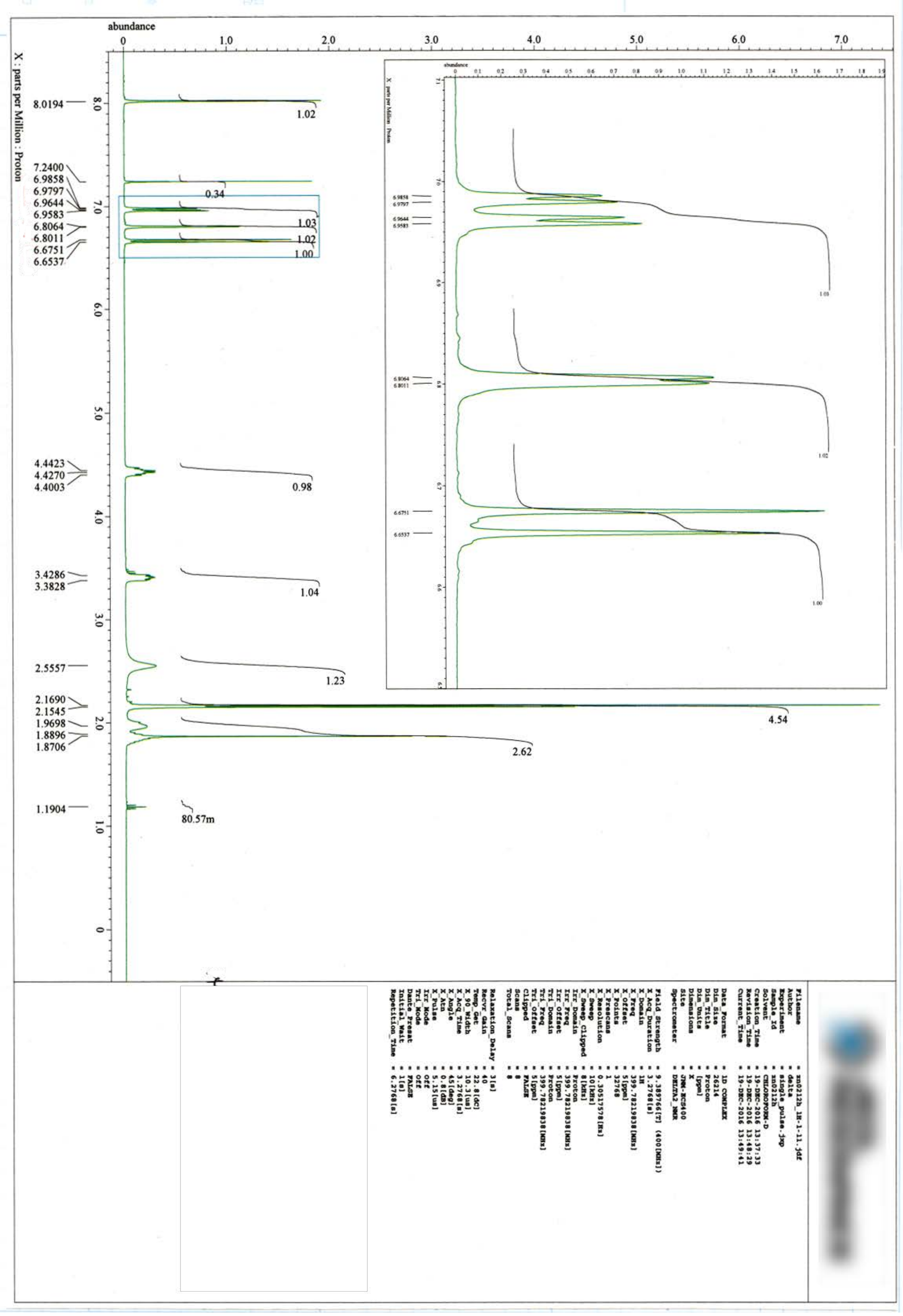

Figure S36. ^{1}H NMR spectrum of Zn($L^{5\text{-}Me}$) in $CDCl_3$.

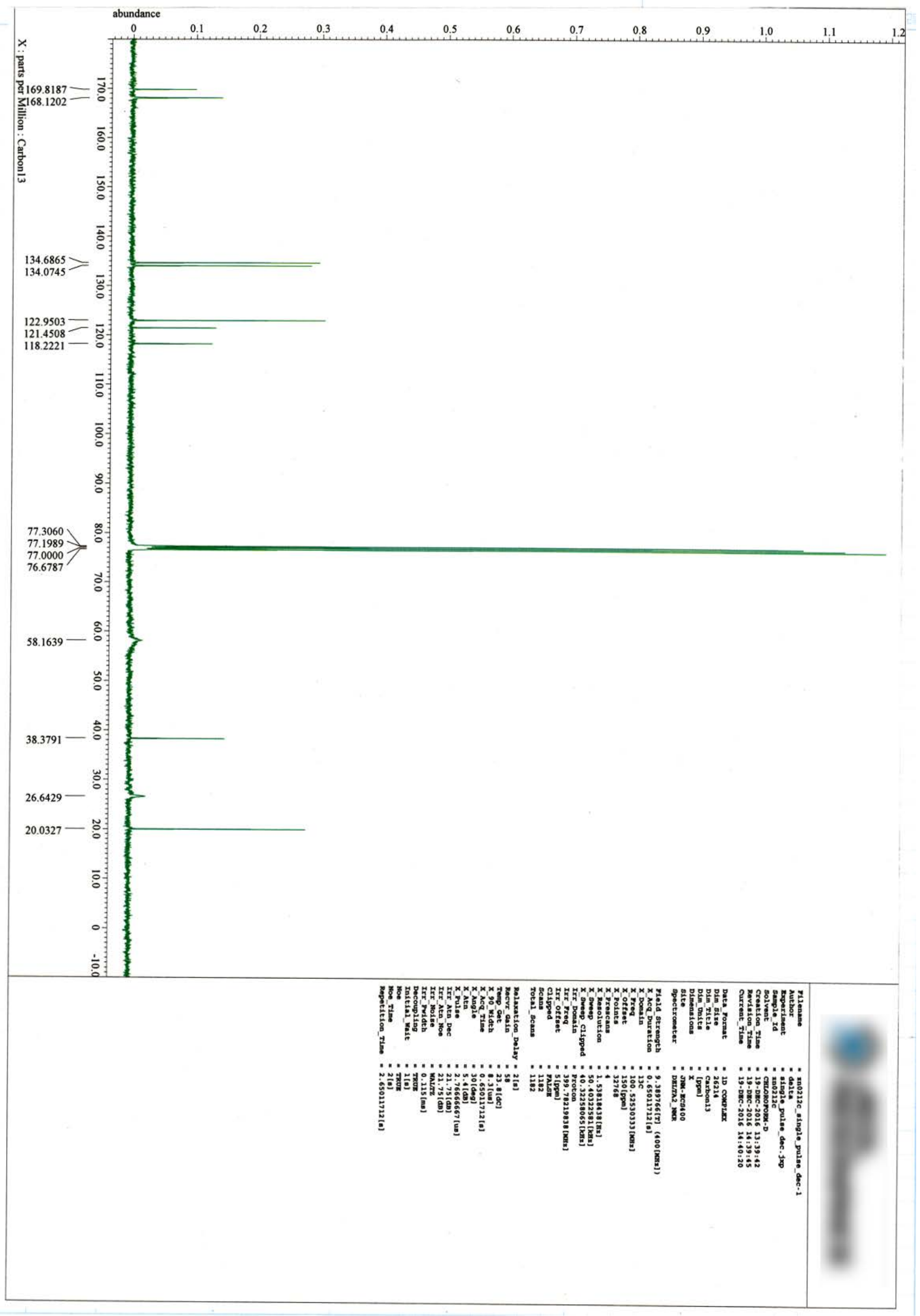

Figure S37. ^{13}C NMR spectrum of Zn(L$^{5\text{-}Me}$) in $CDCl_3$.

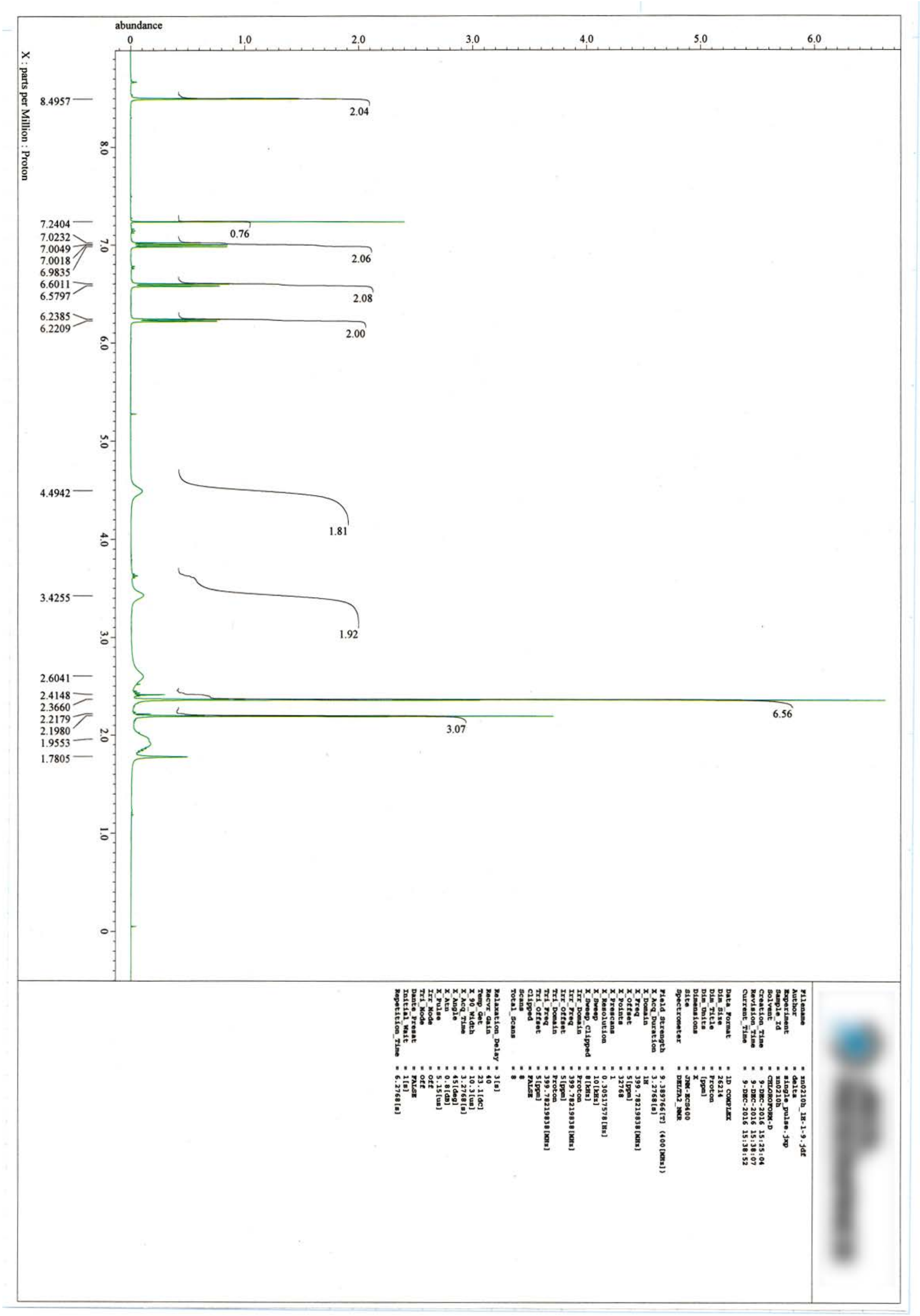

Figure S38. ^1H NMR spectrum of $Zn(L^{6\text{-}Me})$ in $CDCl_3$.

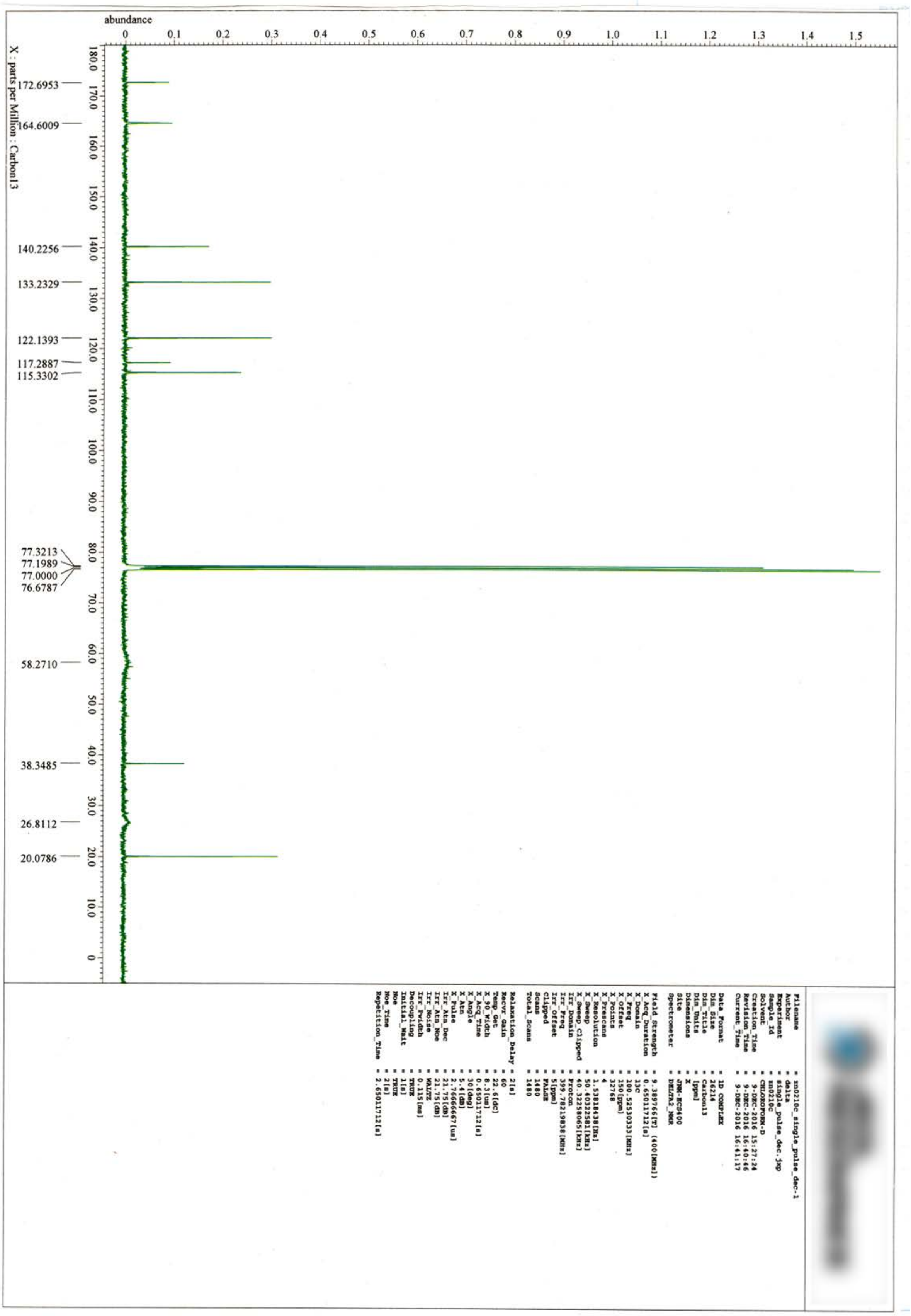

Figure S39. ^{13}C NMR spectrum of $Zn(L^{6-Me})$ in $CDCl_3$.

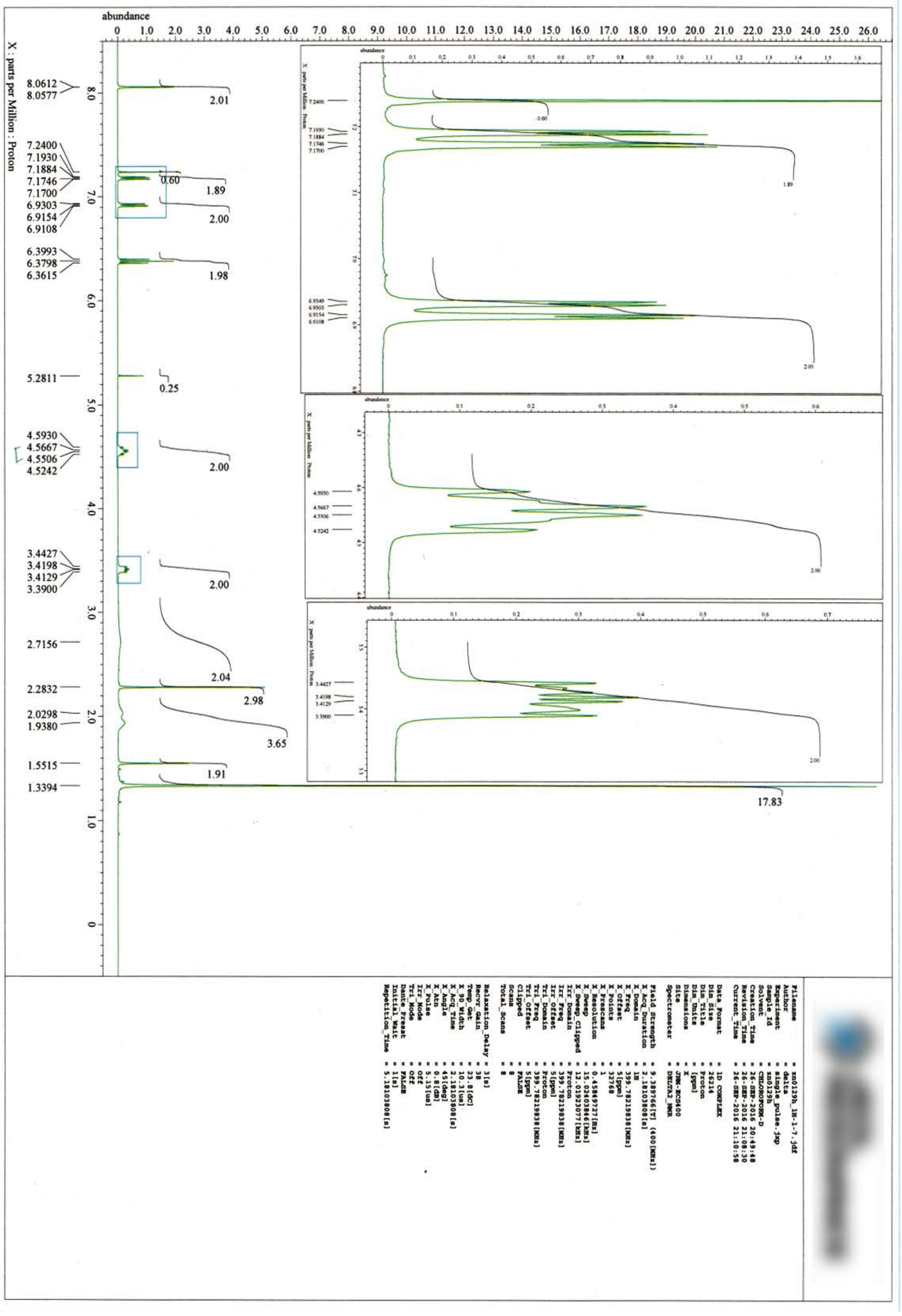

Figure S40. ^{1}H NMR spectrum of Zn($L^{3\text{-}t\text{-}Bu}$) in $CDCl_3$.

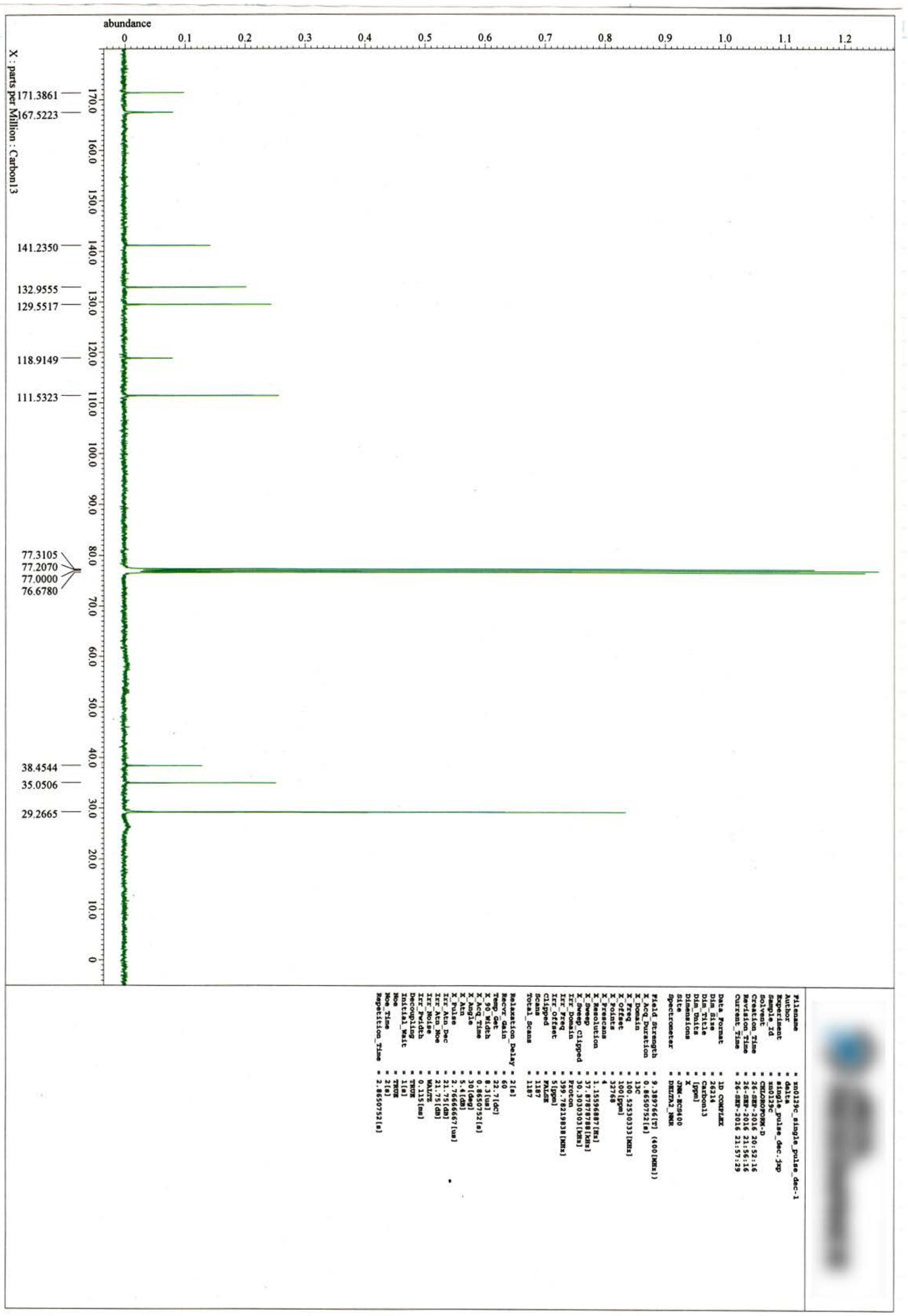

Figure S41. ^{13}C NMR spectrum of $Zn(L^{3\text{-}t\text{-}Bu})$ in $CDCl_3$.

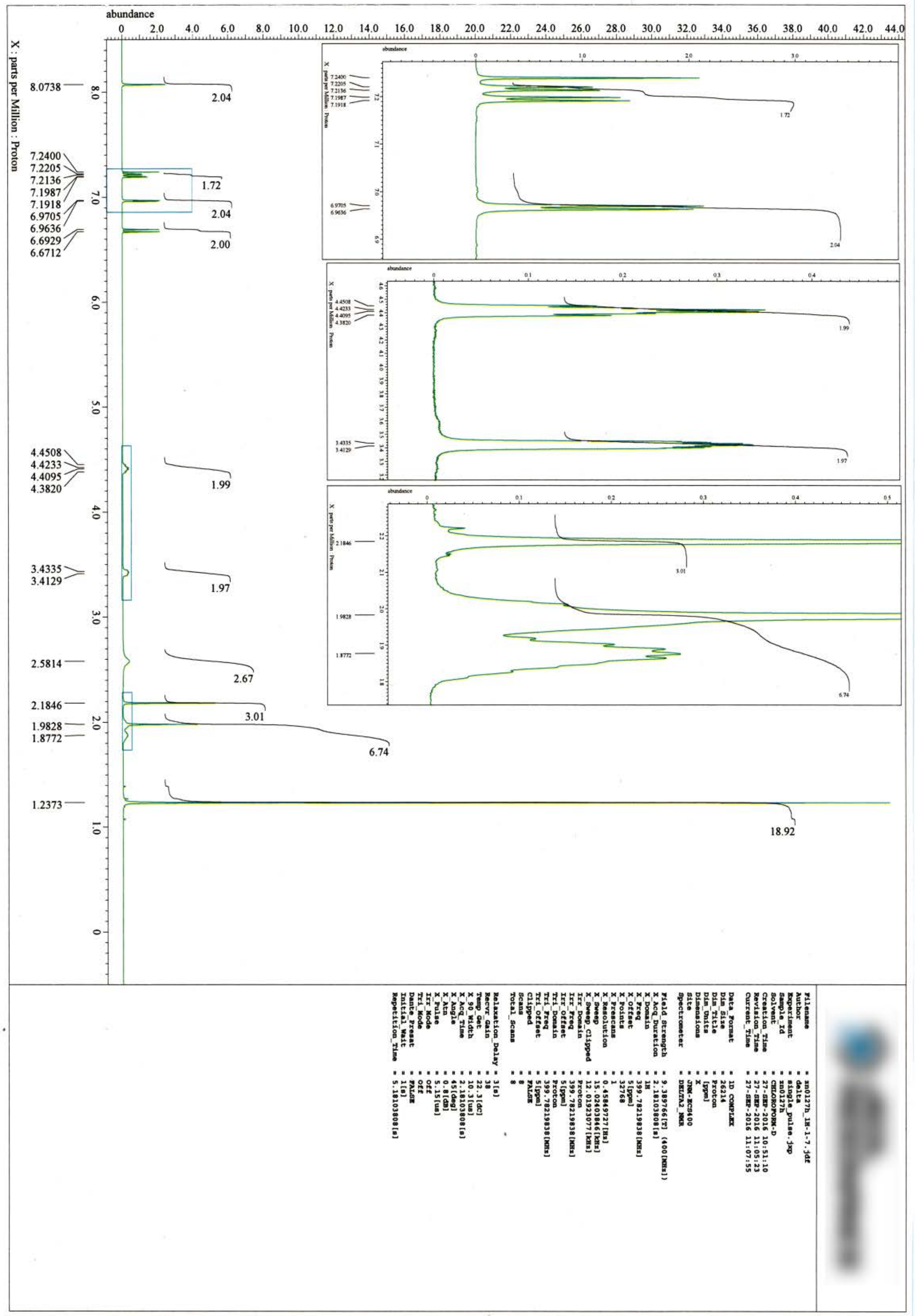

Figure S42. ¹H NMR spectrum of Zn($L^{5\text{-}t\text{-}Bu}$) in $CDCl_3$.

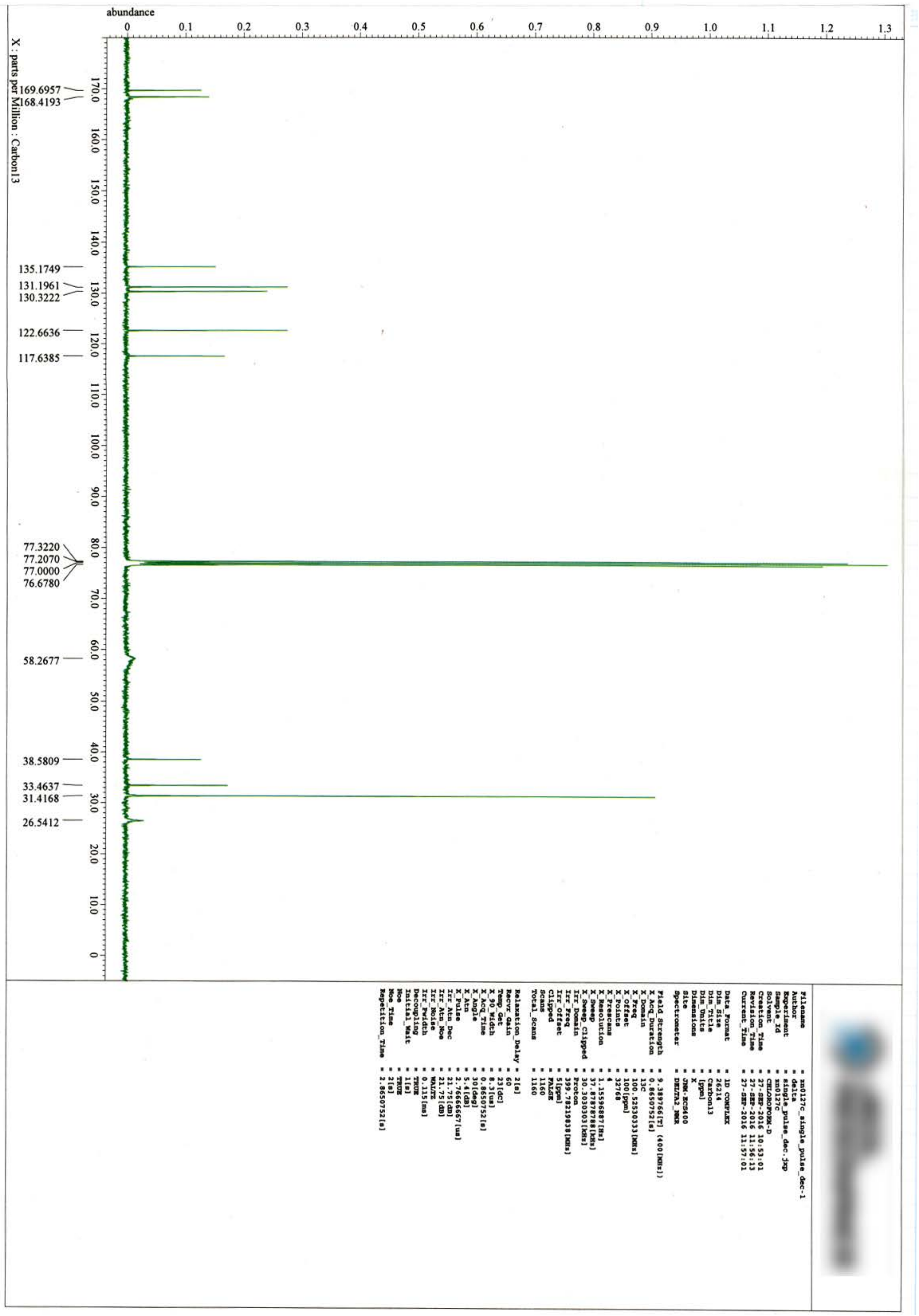

Figure S43. ^{13}C NMR spectrum of Zn($L^{5\text{-}t\text{-}Bu}$) in $CDCl_3$.
